# Self-assembly of dynamic orthoester cryptates

**DOI:** 10.1038/ncomms8129

**Published:** 2015-05-22

**Authors:** René-Chris Brachvogel, Frank Hampel, Max von Delius

**Affiliations:** 1 Department of Chemistry and Pharmacy, Friedrich-Alexander-University Erlangen-Nürnberg (FAU), Henkestrasse 42, 91054 Erlangen, Germany

## Abstract

The discovery of coronands and cryptands, organic compounds that can accommodate metal ions in a preorganized two- or three-dimensional environment, was a milestone in supramolecular chemistry, leading to countless applications from organic synthesis to metallurgy and medicine. These compounds are typically prepared via multistep organic synthesis and one of their characteristic features is the high stability of their covalent framework. Here we report the use of a dynamic covalent exchange reaction for the one-pot template synthesis of a new class of coronates and cryptates, in which acid-labile *O*,*O*,*O*-orthoesters serve as bridgeheads. In contrast to their classic analogues, the compounds described herein are constitutionally dynamic in the presence of acid and can be induced to release their guest via irreversible deconstruction of the cage. These properties open up a wide range of application opportunities, from systems chemistry to molecular sensing and drug delivery.

## Abstract (web summary)

Cryptands and related molecules are macrocyclic polyethers capable of strongly binding cations. Here, the authors use orthoester exchange for the dynamic one-pot synthesis of crypates, which can bind cations and, given their constitutionally dynamic nature, can also be decomposed to release their guest.

## Graphical abstract

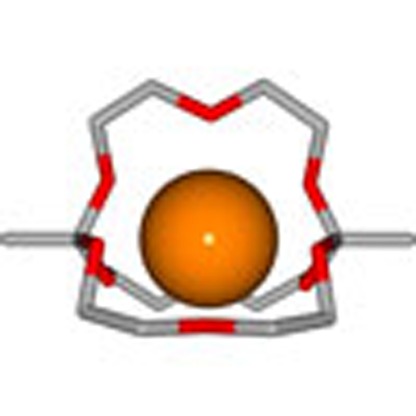

Past progress in supramolecular chemistry has been driven chiefly by the development of new macrocyclic molecules^1^. Pedersen's crown ethers (also called coronands)^2^ and Lehn's cryptands^3^,^4^ (Fig. 1) are excellent examples of compounds that initially served as platforms for studying non-covalent interactions, but have ultimately found widespread application in industry and medicine^5^.

In the last decade, rationally designed three-dimensional cage compounds^6^,^7^,^8^ have become larger and larger^9^,^10^,^11^,^12^,^13^, enabling in one extreme case even the accommodation of a small protein^14^. To achieve the self-assembly of such large structures, the method of choice is dynamic constitutional/covalent chemistry (DCC)^15^,^16^,^17^, which offers the essential feature of error correction that is needed to avoid significant side-product formation. Besides providing high-yielding syntheses, DCC generally gives rise to target structures that are dynamic and responsive to external stimuli under the conditions of their preparation. In the context of the emerging field of systems chemistry^18^,^19^, dynamic macrocycles and cages have served as a valuable testing ground for the investigation and manipulation of complex molecular networks^20^,^21^,^22^,^23^,^24^,^25^. Constitutionally dynamic cryptates^26^,^27^ would represent the smallest and simplest conceivable three-dimensional platform for studying molecular complexity; however, to the best of our knowledge, there are no reports of monometallic cryptates yet, which can be prepared and manipulated based on a dynamic covalent exchange reaction^28^,^29^,^30^,^31^.

Here we describe how orthoester exchange, a previously ignored dynamic covalent reaction^32^, can be used for the one-pot synthesis of monometallic cryptates (for example, see Fig. 1) from strikingly simple starting materials. We provide comprehensive characterization data (including an X-ray structure) for this new class of compounds and report on their dynamic properties, as well as on the formation of orthoester crown ethers as reaction intermediates and the unexpected finding that 4 Å molecular sieves (MS) can act as a source of sodium guest.

## Results

### Template synthesis of a dynamic orthoester cryptate

Inspired by reports on dynamic ‘scaffolding ligands' (*O*,*N*,*P*-orthoesters)^33^,^34^, we have recently investigated the exchange reaction of carboxylic *O*,*O*,*O*-orthoesters with simple alcohols from a DCC perspective^32^. We realized during the course of these studies that the tripodal architecture and dynamic chemistry of orthoesters^35^ could be well suited for establishing two bridgeheads in macrobicyclic compounds (Fig. 1). The synthesis of such orthoester cryptands could be carried out in one step and under thermodynamic control, while a suitable metal ion could serve as a template.

To test these hypotheses, we treated a chloroform solution of two bulk chemicals, trimethyl orthoacetate **(1)** and diethylene glycol (**2**), with catalyst trifluoroacetic acid (TFA) and a stoichiometric metal template (Fig. 2). Analysis of these initial experiments by ^1^H nuclear magnetic resonance (NMR) spectroscopy and electrospray ionization mass spectrometry revealed that complex mixtures, containing the desired cryptate among other exchange products, had formed. Careful optimization of the reaction conditions (use of MS as a thermodynamic sink for water and methanol; use of the ‘non-coordinating' counteranion tetrakis[3,5-bis(trifluoromethyl)phenyl]borate (BArF^−^))^36^,^37^,^38^,^39^,^40^ eventually led to the formation of cryptate [**Na**^**+**^**⊂*****o*****-Me**_**2**_**-1.1.1**]**BArF**^−^ (named in loose analogy to Lehn's classic cryptates; ‘*o*' stands for orthoester) as the predominant reaction product (isolated yields typically 60%–70%).

As shown in the ^1^H NMR spectra presented in Fig. 2, the reversible reaction between **1** and **2** initially generates a remarkable diversity of different exchange products (various degrees of replacement of MeOH by **2**, as well as formation of macrocyclic and oligomeric products (Fig. 2b)) and it is only on slow removal of MeOH by MS (4 Å) that the system converges to the final reaction product (Fig. 2c).

It should be noted that during the exchange process, water has to be excluded from the reaction mixture, which is not trivial to achieve, because even rigorously dried MS tend to slowly release residual water. As a consequence, hydrolysis of **1** can lead to the slow formation of methyl acetate (MeOAc) as a side product (Fig. 2c). From a preparative standpoint, the formation of MeOAc is not a problem, because it can easily be removed under reduced pressure (Fig. 2d). In addition, its formation as the sole side product provides two valuable pieces of information regarding the dynamic system under study. First, it is remarkable that we find the cryptate as the exclusive reaction product, even though the partial decomposition of orthoester **1** leads to a non-ideal ratio of orthoester to diol (ideal value 2:3). This result indicates that there is a thermodynamic bias for the formation of the final cryptate in the presence of sodium template. Second, the fact that we observe only one type of ester (MeOAc) suggests that exchange products incorporating one or more diethylene glycol chains (generated much more rapidly than MeOAc) are kinetically stabilized against hydrolysis, presumably due to (chelate) binding of sodium.

This kinetic stabilization due to metal binding^41^ is most pronounced in pristine cryptate [**Na**^**+**^**⊂*****o*****-Me**_**2**_**-1.1.1**]**BArF**^−^. For example, when we mixed [**Na**^**+**^**⊂*****o*****-Me**_**2**_**-1.1.1**]**BArF**^−^ with trimethyl orthoacetate (**1**) in water-saturated chloroform, only the simple orthoester **1** was found to hydrolyse, whereas cryptate [**Na**^**+**^**⊂*****o*****-Me**_**2**_**-1.1.1**]**BArF**^**−**^ remained stable for 7 days (Supplementary Fig. 2). In the absence of acid, the cage is in fact stable in dimethyl sulfoxide/water mixtures and can be purified by silica gel chromatography (Fig. 2e). These observations are highly unusual for *O*,*O*,*O*-orthoesters that are not stabilized by the presence of five- or six-membered rings (as in Corey's OBO protecting group)^42^. These properties imply that orthoester-based hosts could have a unique advantage over existing coronands and cryptands: charged guests could be transported across lipophilic membranes^43^ and subsequent hydrolysis would trigger the release of the guest ([**Na**^**+**^**⊂*****o*****-Me**_**2**_**-1.1.1**]**BArF**^**−**^ hydrolyses readily in the presence of excess water and acid; Supplementary Fig. 3). The high potential for such a supramolecular approach for drug formulation and delivery is underscored by a recent patent publication, which describes related hydrolysis-prone crown ether compounds (scheduled for phase 1 clinical trials in 2015)^44^.

### Solid-state structure

Following the comprehensive characterization of [**Na**^**+**^**⊂*****o*****-Me**_**2**_**-1.1.1**]**BArF**^**−**^ by NMR spectroscopy and mass spectrometry (Supplementary Figs 4–13), we turned our attention towards obtaining further structural and dynamic insights on this compound. To our delight, single crystals of [**Na**^**+**^**⊂*****o*****-Me**_**2**_**-1.1.1**]**BArF**^**−**^ suitable for X-ray crystallography could be obtained by slow diffusion of cyclopentane into dilute solutions in chloroform or dichloromethane. The solid-state structure (Fig. 3a, Supplementary Fig. 14, Supplementary Tables 1 and 2, and Supplementary Data 1) shows that the sodium ion is bound to all nine surrounding oxygen atoms with a mean Na–O bond length of 2.56** **Å, which is very close to the mean Na–O distance of 2.57 Å found in the solid-state structure of Lehn's classic cryptate [Na^+^⊂2.2.2]I^**−**^ (ref. 45). An interesting question arises from the relatively small distances between the three diethylene glycol chains (O–O distance between two chains: 4.5 Å; see space-filling model in Fig. 3a) and the relatively rigid architecture of the cage (in contrast to classic cryptates, no inversion is possible at the terminus of the cage): can the metal ion exit from the ***o*****-Me**_**2**_**-1.1.1** cage under ambient conditions? Or in other words, is **[Na**^**+**^**⊂*****o*****-Me**_**2**_**-1.1.1**]**BArF**^**−**^ in fact a carceplex, not a cryptate?

### Thermodynamics and kinetics of guest exchange

To answer this question, we carried out competition experiments in which complexation agents such as Lehn's cryptate 2.2.1 (Na^+^ binding constant *K*_A_=10^13^ M^**−**1^ in D_2_O-saturated CDCl_3_)^46^ were titrated to a freshly deacidified solution of [**Na**^**+**^**⊂*****o*****-Me**_**2**_**-1.1.1**]**BArF**^**−**^ in chloroform. As shown in Fig. 3b (top), addition of classic cryptand 2.2.1 to our orthoester cryptate led to quantitative formation of cryptate [Na^+^⊂2.2.1]BArF^**−**^ and orthoester cryptand ***o*****-Me**_**2**_**-1.1.1**, indicating that 2.2.1 has a significantly higher binding constant under these conditions. The reaction outcome also confirms that sodium can exit from the orthoester cage and the observed ^1^H NMR spectra demonstrate that in this experiment sodium ion exchange is slow between the two competing cryptands. Following such a titration, we treated a 1:1 mixture of [Na^+^⊂2.2.1]BArF^**−**^ and ***o*****-Me**_**2**_**-1.1.1** with catalytic TFA, resulting in the complete conversion of ***o*****-Me**_**2**_**-1.1.1** into orthoester products featuring eight-membered rings (Supplementary Fig. 19). This experiment demonstrates that ***o*****-Me**_**2**_**-1.1.1**, unlike [**Na**^**+**^**⊂*****o*****-Me**_**2**_**-1.1.1**]**BArF**^**−**^, does not represent a thermodynamic minimum and thus cannot be prepared without template from compounds **1** and **2** via reversible orthoester exchange.

In a second competition experiment, we titrated weaker complexation agent 15-crown-5 (Na^+^ binding constant *K*_A_=10^5^ M^**−**1^ in acetonitrile)^46^ to cryptate [**Na**^**+**^**⊂*****o*****-Me**_**2**_**-1.1.1**]**BArF**^**−**^. As evident from the series of ^1^H NMR spectra (Fig. 3b, bottom), at equimolar addition of 15-crown-5 the equilibrium lies on the side of the orthoester cryptate, although broadening of the peaks indicates that exchange of sodium is fast in this case. Titration with up to 20 equivalents of 15-crown-5 gave rise to binding isotherms, from which we could deduce that the binding constant of ***o*****-Me**_**2**_**-1.1.1** is about one order of magnitude higher than that of 15-crown-5 (see Supplementary Figs 20–22 for further thermodynamic data). In a pristine mixture of cryptate [**Na**^**+**^**⊂*****o*****-Me**_**2**_**-1.1.1**]**BArF**^**−**^ and cryptand ***o*****-Me**_**2**_**-1.1.1** (*vide infra* for preparation method), we were able to determine an exchange rate of 0.6 s^**−**1^ for sodium exchange between degenerate orthoester cryptands (NMR exchange spectroscopy (EXSY); Supplementary Fig. 23 and Supplementary Note 1).

### Other metal templates and orthoester crown ethers

To confirm that a metal template effect is responsible for the remarkably clean formation of [**Na**^**+**^**⊂*****o*****-Me**_**2**_**-1.1.1**]**BArF**^**−**^ (Fig. 1), we studied the exchange reaction between **1** and **2** in the absence of template and in the presence of different metal templates under otherwise identical reaction conditions. As shown in Fig. 4 (top), the exchange reaction without template mainly gave rise to exchange product **3**, a monomeric orthoester featuring an eight-membered ring that results from one molecule of diethyleneglycol (**2**) having displaced two molecules of methanol (Supplementary Fig. 24). Theoretically, it should be possible to remove the last equivalent of methanol by increasing the time during which the mixture is exposed to MS, but we found that the system has a strong tendency to remain at this particular state (that is, product **3**). However, when such a dynamic mixture was treated with one equivalent of Sodium tetrakis[3,5-bis(trifluoromethyl)phenyl]borate (NaBArF), the dynamic system responded by forming cryptate [**Na**^**+**^**⊂*****o*****-Me**_**2**_**-1.1.1**]**BArF**^**−**^ quantitatively and in a relatively short time (Fig. 4, right-hand side, and Supplementary Fig. 25).

When we used metal salts LiTPFPB, NaBArF or KBArF for the self-assembly reaction, we observed that after 2–3 days reaction time two distinct reaction products had formed in a 1:1 ratio. Careful analysis of one- and two-dimensional NMR spectra, as well as high-resolution mass spectrometry, indicated that unprecedented orthoester crown ethers^1^ ***o*****-Me**_**2**_**-(OMe)**_**2**_**-16-crown-6** had formed as a mixture of *syn* and *anti* diastereomers (Fig. 4, centre). These crown ethers are the main products at a stage of the reaction where two equivalents of methanol have been removed through the effect of MS (2–3 days reaction time). The dynamic chemical system is thus not only responsive to the described metal template effect, but also to the precise quantity of available methanol.

### Molecular sieves (4 Å) as an unexpected sodium source

To our initial surprise, the crown ethers originating from the lithium and potassium salts eventually transformed into the corresponding sodium cryptates [**Na**^**+**^**⊂*****o*****-Me**_**2**_**-1.1.1**]**X**^**−**^ (Fig. 4, X^**−**^=tetraarylborate anion). Using mass spectrometry, ^23^Na and ^7^Li NMR spectroscopy and, most notably, atom absorption and emission spectroscopy, we were able to confirm that these cage compounds were indeed the sodium cryptates [**Na**^**+**^**⊂*****o*****-Me**_**2**_**-1.1.1**]**X**^**−**^ (Supplementary Figs 26 and 27), and that the sodium source is type A zeolite (4 Å MS)^47^, a porous framework material that contains accessible sodium ions. A search of the literature revealed several reports on this type of ion exchange in aqueous^47^,^48^ and one report in organic medium^49^. When we used metal salt KBArF in conjunction with MS 3 Å, which contain potassium instead of sodium ions, we did not observe self-assembly of a potassium cryptate from starting materials **1** and **2**. Collectively, our experiments with different metal ions point towards a pronounced preference for sodium cryptate [**Na**^**+**^**⊂*****o*****-Me**_**2**_**-1.1.1**] over the potential lithium or potassium cryptates, which could be explained by the differences in effective ionic radii between lithium (0.9 Å), sodium (1.2 Å) and potassium (1.6 Å)^50^. Only sodium appears to have the right size for forming nine efficient metal–oxygen bonds, while not inducing energetically costly conformations within the organic host.

### Preliminary experiments on scope and limitations

We conducted preliminary studies on the scope of the described orthoester cryptates and coronates. A cryptate derived from orthoester trimethyl orthopropanoate, [**Na**^**+**^**⊂*****o*****-Et**_**2**_**-1.1.1**]**BArF**^**−**^ (terminal substituent: Et), could be prepared without difficulty, following a similar procedure to that used for cryptate [**Na**^**+**^**⊂*****o*****-Me**_**2**_**-1.1.1**]**BArF**^**−**^. Using trimethyl orthoformate as the starting material (terminal substituent: H) led to the formation of remarkably stable and pure crown ether complexes [**Na**^**+**^**⊂*****o*****-H**_**2**_**-(OMe)**_**2**_**-16-crown-6**], which did not further react to the corresponding cryptates under our standard conditions. A notable limitation of the self-assembly reaction concerns the counteranion of the sodium template. Thus far, we were able to prepare cryptate [**Na**^**+**^**⊂*****o*****-Me**_**2**_**-1.1.1**]**X**^**−**^ with three different tetraarylborate anions (X^**−**^=BArF^**−**^, TPFPB^**−**^ and tetrakis(4-chlorophenyl)borate), while simpler anions such as PF_6_^**−**^ or BF_4_^**−**^ have failed, suggesting that a truly ‘non-coordinating'^37^ anion needs to be present during self-assembly.

In an attempt to exchange the counteranion in pristine [**Na**^**+**^**⊂*****o*****-Me**_**2**_**-1.1.1**]**BArF**^**−**^ to chloride, we discovered a convenient method for preparing cryptand ***o*****-Me**_**2**_**-1.1.1** (Fig. 4, bottom right). When a solution of [**Na**^**+**^**⊂*****o*****-Me**_**2**_**-1.1.1**]**BArF**^**−**^ in CDCl_3_ was treated with anion exchange resin Lewatit MP-64 (Cl form), we observed the clean formation of ***o*****-Me**_**2**_**-1.1.1**, for which the precipitation of NaCl is presumably the driving force. With pristine ***o*****-Me**_**2**_**-1.1.1** in our hands, we were able to prepare cryptate [**Li**^**+**^**⊂*****o*****-Me**_**2**_**-1.1.1**]**TPFPB**^**−**^ by exposing the cryptand to a solution of the lithium salt (Fig. 4, bottom right; structure confirmed by ^1^H/^7^Li hetero nuclear overhauser effect NMR spectroscopy; Supplementary Fig. 28). The lithium cryptate could be transformed back into [**Na**^**+**^**⊂*****o*****-Me**_**2**_**-1.1.1**]**BArF**^**−**^ by addition of one equivalent of NaBArF, confirming the preference of this cryptand for Na^+^. Based on preliminary NMR data, K^+^ (salt: KBArF) appears to not enter the cage, but presumably ‘nests' on the crown-ether-type faces of the cryptand. Further experiments to such ends are ongoing in our laboratory.

## Discussion

We have shown that a dynamic system based on two strikingly simple organic starting materials converges to three distinct types of exchange products under the influence of dry MS: (i) in the absence of a template, a simple exchange product featuring an eight-membered ring is formed; (ii) in the presence of a sodium template, an unprecedented dynamic orthoester cryptate is formed, in which nine oxygen donors are bound to the metal ion; (iii) *en route* to the sodium cryptates, novel orthoester coronates can be observed and, in some cases, isolated as a mixture of *syn* and *anti* isomers. Sodium cryptate [**Na**^**+**^**⊂*****o*****-Me**_**2**_**-1.1.1**]**BArF**^**−**^ was found to be surprisingly stable against water in neutral solution, but susceptible to hydrolysis in the presence of water and acid. We believe that this property will make orthoester cages useful for the traceless delivery of cations into biological systems^44^. Competition experiments in solution suggest that the encapsulated metal ion is in slow exchange with the bulk (*k*_obs_=0.6 s^**−**1^) and the binding constant for Na^+^ lies between the classic complexation agents 15-crown-5 and 2.2.1. Besides their interesting structural and dynamic properties, orthoester cryptates offer preparative advantages over their classic analogues: their synthesis relies on a one-pot, dynamic covalent ring-closing reaction, and substituted cages ***o*****-R**_**2**_**-1.1.1** are accessible simply by using different orthoesters as starting materials. We are currently working towards further diversifying the target structures, as well as increasing the dynamic system's complexity (for example, self-sorting and response to external stimuli).

## Methods

### Preparation of stock solutions

NaBArF (0.14 mmol, 127.8 mg) and diethylene glycol (0.42 mmol, 39.9 μl) were dissolved in 14 ml CDCl_3_ and the mixture was dried over MS (4 Å, 1 g) for 3 days. TFA (0.24 mmol, 18.4 μl) was dissolved in CDCl_3_ (total volume 2.00 ml).

### Self-assembly of cryptate [Na^+^⊂*o*-Me_2_-1.1.1]BArF^−^

To 6 ml of stock solution were added fresh MS (4 Å, 1 g) and the reaction mixture was left to stand at room temperature. After 16 h, 1 mol% TFA (10 μl) was added from stock solution, the mixture was shaken and trimethyl orthoacetate (0.12 mmol, 15.4 μl) was added. Every 24 h, 1 mol% TFA was added to keep the exchange reaction active (MS slowly transform the acid catalyst into inactive anhydride and/or esters). The reaction progress was monitored regularly by ^1^H NMR spectroscopy. After 5 days, the solvent was removed under reduced pressure and [**Na**^**+**^**⊂*****o*****-Me**_**2**_**-1.1.1**]**BArF**^**−**^ was obtained as a colourless solid (67% yield). Characterization data: M.p. 124 °C –128 °C. ^1^H NMR (400 MHz, CDCl_3_, 298 K): *δ*=7.68 (*t*, *J*=2.8 Hz, 8H), 7.51 (*s*, 4H), 3.79–3.77 (*m*, 12H), 3.50–3.48 (*m*, 12H), 1.43 p.p.m. (*s*, 6H). ^13^C NMR (100 MHz, CDCl_3_, 298 K): *δ*=162.8, 162.3, 161.8, 161.3, 135.1, 129.7, 129.4, 129.1, 128.9, 128.8, 126.2, 123.5, 120.8, 117.7, 113.0, 69.2, 62.0, 17.7 p.p.m. ^11^B NMR (128 MHz, CDCl_3_, 298 K): *δ*=−6.7 p.p.m. ^19^F NMR (282 MHz, CDCl_3_, 298 K): *δ*=−62.1 p.p.m. ^23^Na NMR (132 MHz, CDCl_3_, 298 K): *δ*=−5.9 p.p.m. HRMS (ESI^+^): *m*/*z*=389.1794 [M+Na]^+^ (calcd. 389.1782 for C_16_H_30_O_9_Na). For further experimental details and characterization data, see Supplementary Methods and Supplementary Figs 29–47.

### Exclusion of moisture

Molecular sieves were dried by heating for 3 days at 150 °C under reduced pressure (10^**−**2^ mbar). All solvents were dried over MS for at least 24 h. All orthoester exchange reactions (catalysed by TFA) were carried out under nitrogen. After the acid was quenched (for example, by addition of triethylamine or basic aluminum oxide), most orthoester complexes described herein were found to be unusually stable against water and could be handled on the benchtop without further precautions (Supplementary Fig. 2).

## Additional information

**Accession codes:** The X-ray crystallographic coordinates for structures reported in this study have been deposited at the Cambridge Crystallographic Data Centre (CCDC), under CCDC number 1038394. These data can be obtained free of charge from The Cambridge Crystallographic Data Centre via www.ccdc.cam.ac.uk/data_request/cif.

**How to cite this article:** Brachvogel, R.-C. *et al*. Self-assembly of dynamic orthoester cryptates. *Nat. Commun.* 6:7129 doi: 10.1038/ncomms8129 (2015).

## Supplementary Material

Supplementary Information Supplementary Figures 1-47, Supplementary Tables 1-2, Supplementary Note 1, Supplementary Methods and Supplementary References

Supplementary Data 1Crystallographic Information File for 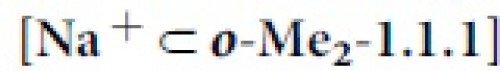

## Figures and Tables

**Figure 1:**
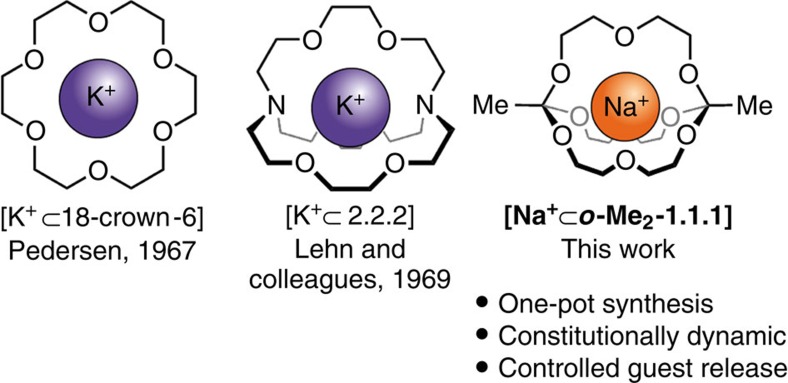
Coronates and cryptates. Comparison of a classic coronate and cryptate with one of the orthoester-based, constitutionally dynamic cryptates described in this work.

**Figure 2:**
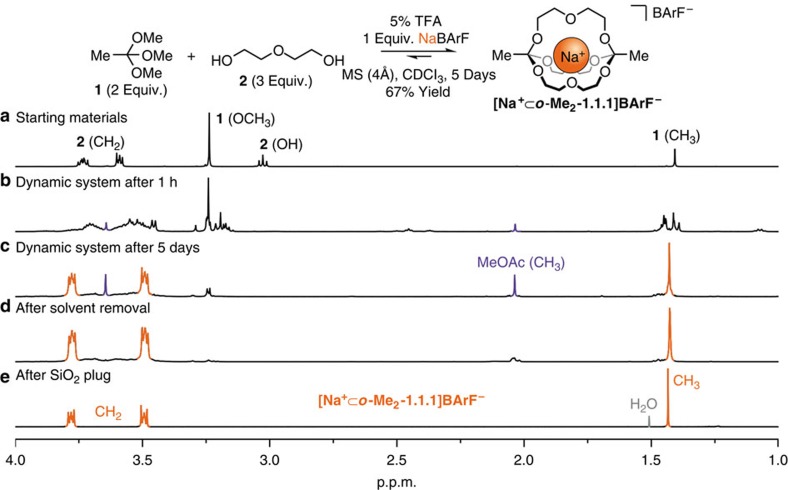
One-pot self-assembly of dynamic cryptate [Na^+^⊂*o*-Me_2_-1.1.1]BArF^−^. Partial ^1^H NMR spectra (400 MHz, CDCl_3_, 298 K) showing the evolution of the dynamic system over time (see Supplementary Fig. 1 for full spectra). (**a**) Starting materials. (**b**) Complex mixture that forms rapidly after addition of acid catalyst (1 h). (**c**) After 5 days [**Na**^**+**^**⊂*****o*****-Me**_**2**_**-1.1.1**]**BArF**^**−**^ is formed as major product, alongside hydrolysis product MeOAc (singlets at 3.6 and 2.0 p.p.m.). (**d**) The crude product is obtained by removal of MeOAc under reduced pressure. (**e**) Further purification is conveniently achieved by passing the crude product through a short plug of silica gel. Reaction conditions: trimethyl orthoacetate (120 μmol), diethylene glycol (180 μmol), NaBArF (60 μmol), TFA (3.0 μmol; added over 5 days), MS (4 Å, 1 g), CDCl_3_ (6.0 ml), 5 days, room temperature.

**Figure 3:**
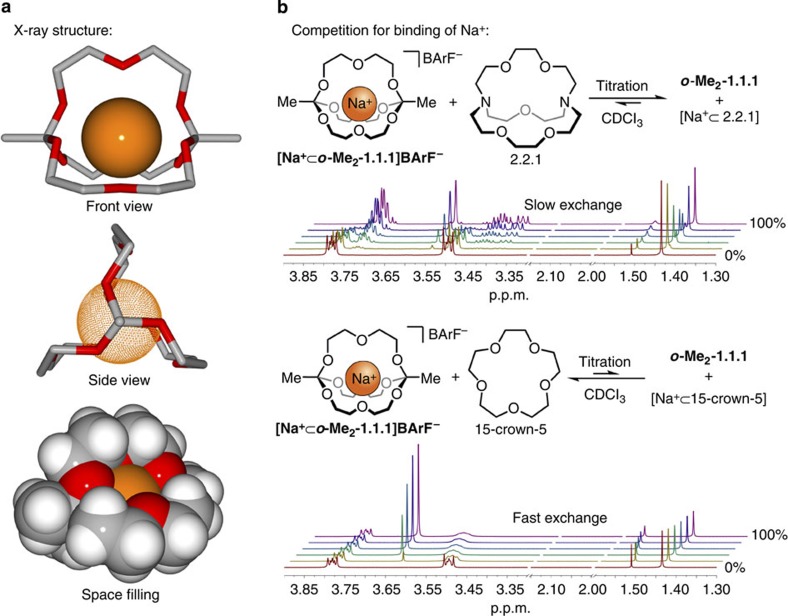
Solid-state structure of [Na^+^⊂*o*-Me_2_-1.1.1]BArF^−^ and competition experiments with classic hosts for Na^+^. (**a**) Solid-state structure determined by single-crystal X-ray diffraction. Crystals were obtained by slow diffusion of cyclopentane into dichloromethane. Counteranion, hydrogen atoms and disorder (along the CH_2_-CH_2_-O-CH_2_-CH_2_ chains, disorder with a population of 1:1 was observed; see Supplementary Fig. 14) are omitted for clarity. Oxygen atoms are shown in red, carbon atoms in grey. Sodium (orange) is shown at 65% of the van der Waals radius in stick model representations. Na–O bond lengths: 2.51–2.58 Å (six orthoester oxygens), 2.55–2.62 Å (three chain oxygens). (**b**) ^1^H NMR titration experiments (400 MHz, CDCl_3_, 298 K) using classic complexation agents 2.2.1 (top) and 15-crown-5 (bottom). Pristine orthoester cryptate is shown at the front, 100% addition of competing host is shown at the back. The region around 2.1 p.p.m. is included to demonstrate that orthoester hydrolysis is not occurring during these experiments (despite the presence of water; singlet at 1.6 p.p.m.). For details, see Supplementary Figs 15–18.

**Figure 4:**
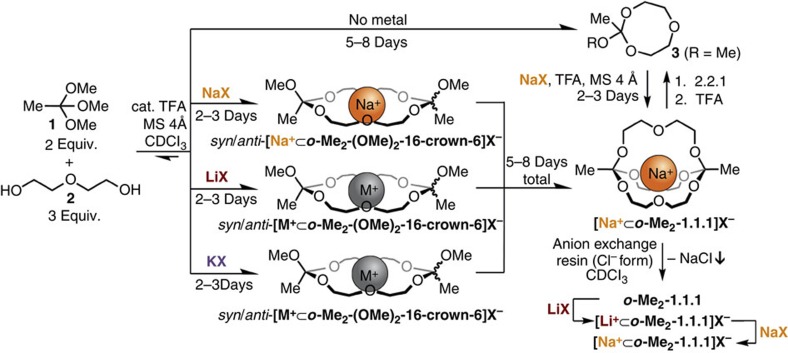
Complex behaviour of a dynamic orthoester system. Structures of predominant products as a function of time, template and other chemical stimuli. Reaction conditions: trimethyl orthoacetate (120 μmol), diethylene glycol (180 μmol), metal salt (60 μmol), TFA (0.6 μmol added per day), MS (4 Å, 1 g), CDCl_3_ (6.0 ml), room temperature. Eight-membered ring products (**3** for R=Me) could be quantitatively converted into cryptate [**Na**^**+**^**⊂*****o*****-Me**_**2**_**-1.1.1**]**X**^**−**^ and vice versa. Pristine cryptand ***o*****-Me**_**2**_**-1.1.1** could be prepared using anion exchange resin Lewatit MP64 (chloride form). Cryptand ***o*****-Me**_**2**_**-1.1.1** could be transformed into lithium cryptate [**Li**^**+**^**⊂*****o*****-Me**_**2**_**-1.1.1**]**X**^**−**^ and from there back into [**Na**^**+**^**⊂*****o*****-Me**_**2**_**-1.1.1**]**X**^**−**^. NaX: NaBArF; LiX: LiTPFPB (lithium tetrakis(pentafluorophenyl)borate); KX: KBArF; M^+^: mixture of Na^+^ with residual Li^+^ or K^+^ due to cation exchange with MS.
