# Supplementary material for: Self-assembly of dynamic orthoester cryptates
Source: Nat Commun. 2015 May 22;6:7129. doi: 10.1038/ncomms8129 (PMC4455094; doi:10.1038/ncomms8129)
Supplement: Supplementary Information — Supplementary Figures 1-47, Supplementary Tables 1-2, Supplementary Note 1, Supplementary Methods and Supplementary References [file ncomms8129-s1.pdf]

Supplementary Figures

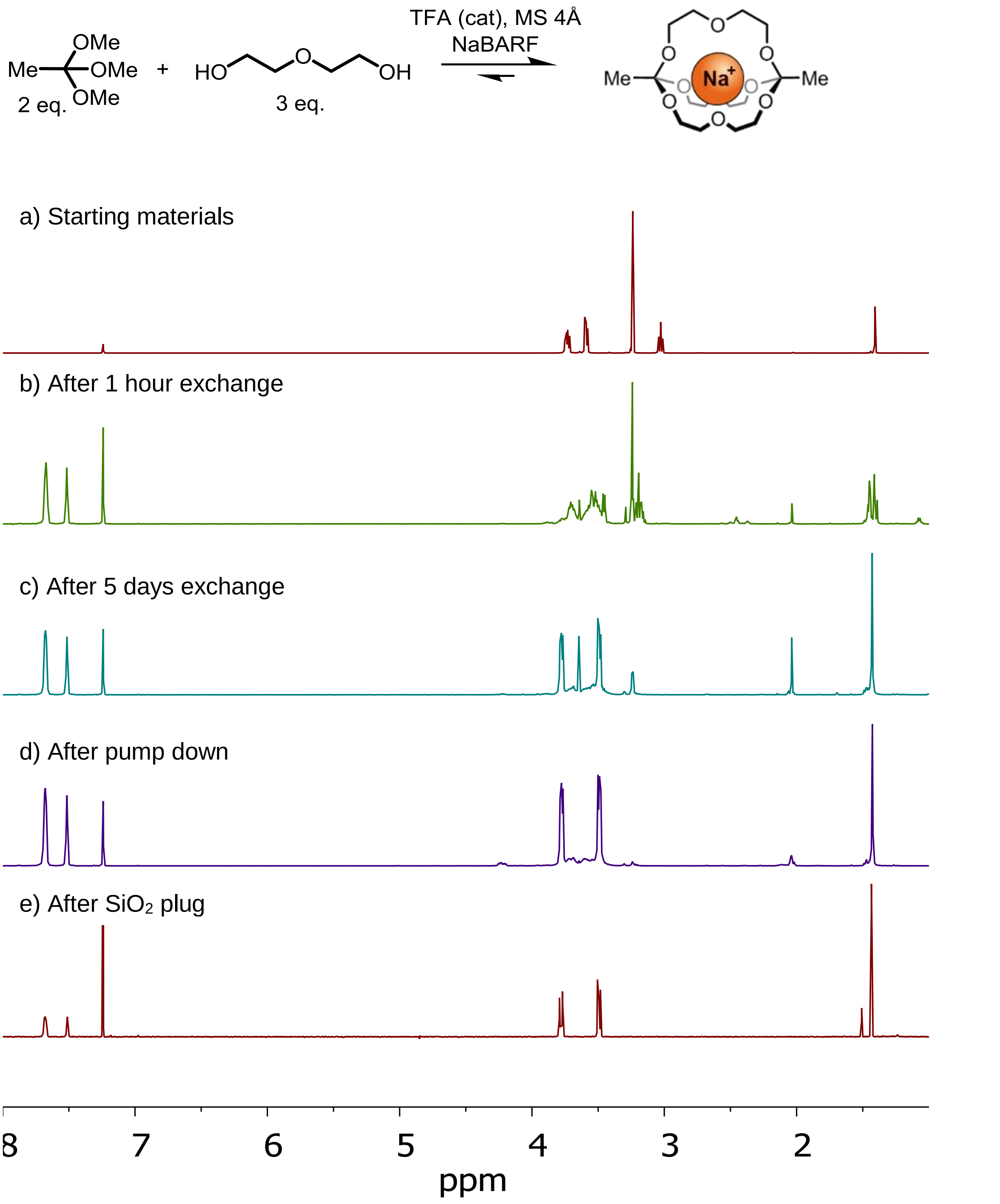

Supplementary Figure 1 | Self-assembly of [Na$^+$⊂*o*-Me$_2$-1.1.1]BArF$^-$. ^{1}H NMR spectra (400 MHz, $CDCl_3$, 298 K).

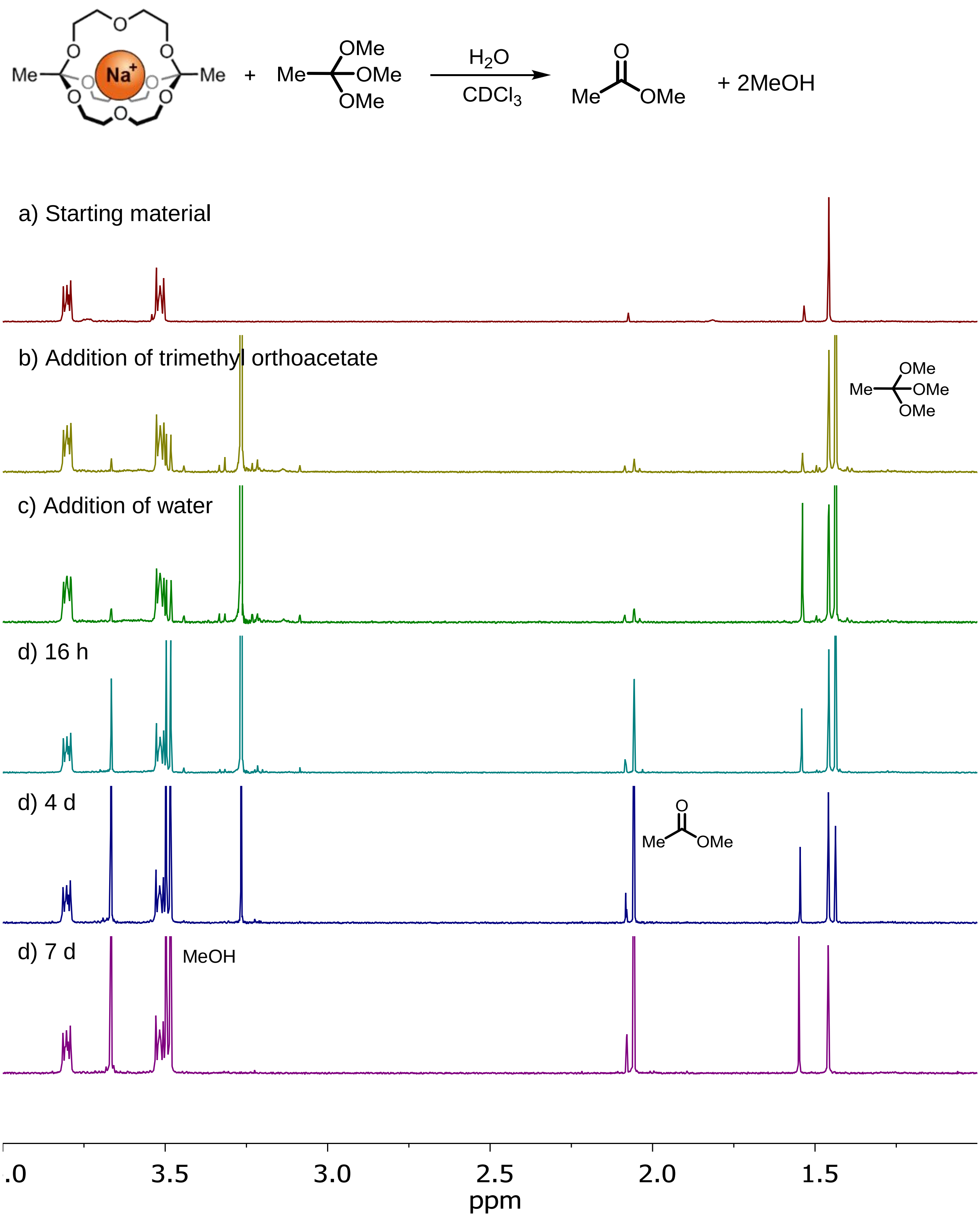

Supplementary Figure 2 | Stability of [Na$^+$⊂*o*-Me$_2$-1.1.1]BArF$^-$ against water. ^{1}H NMR spectra (400 MHz, $CDCl_3$, 298 K). The cryptate remains essentially stable, while trimethyl orthoacetate hydrolyzes completely (hydrolysis is likely catalyzed by trace acid in $CDCl_3$).

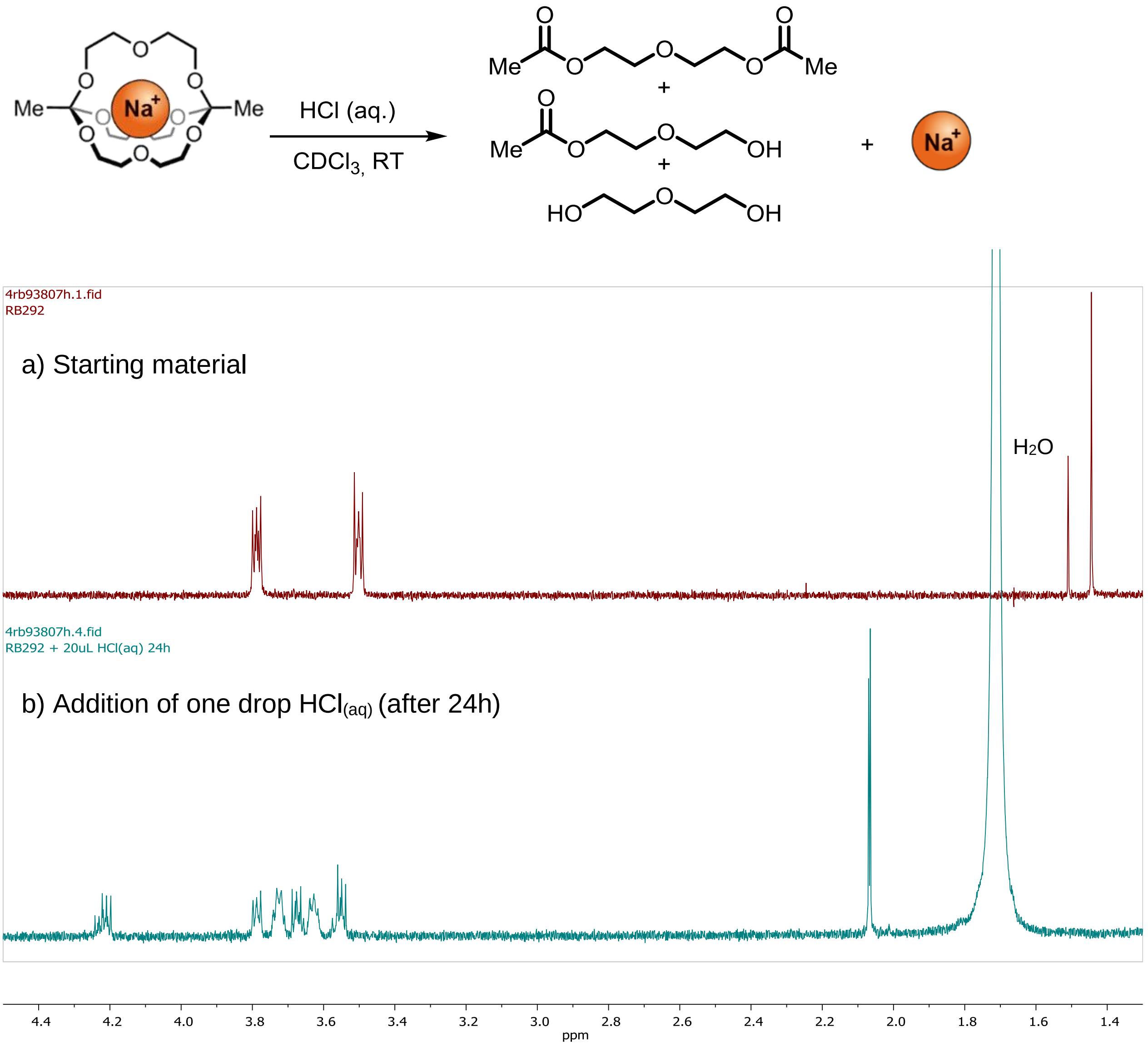

Supplementary Figure 3 | Controlled release of guest via acidic hydrolysis. ^{1}H NMR spectra (400 MHz, $CDCl_3$, 298 K). The cryptate hydrolyzes to diethylene glycol, dimethyl diacetate and 2-(2-hydroxyethoxy)ethyl acetate under the influence of acid and water.

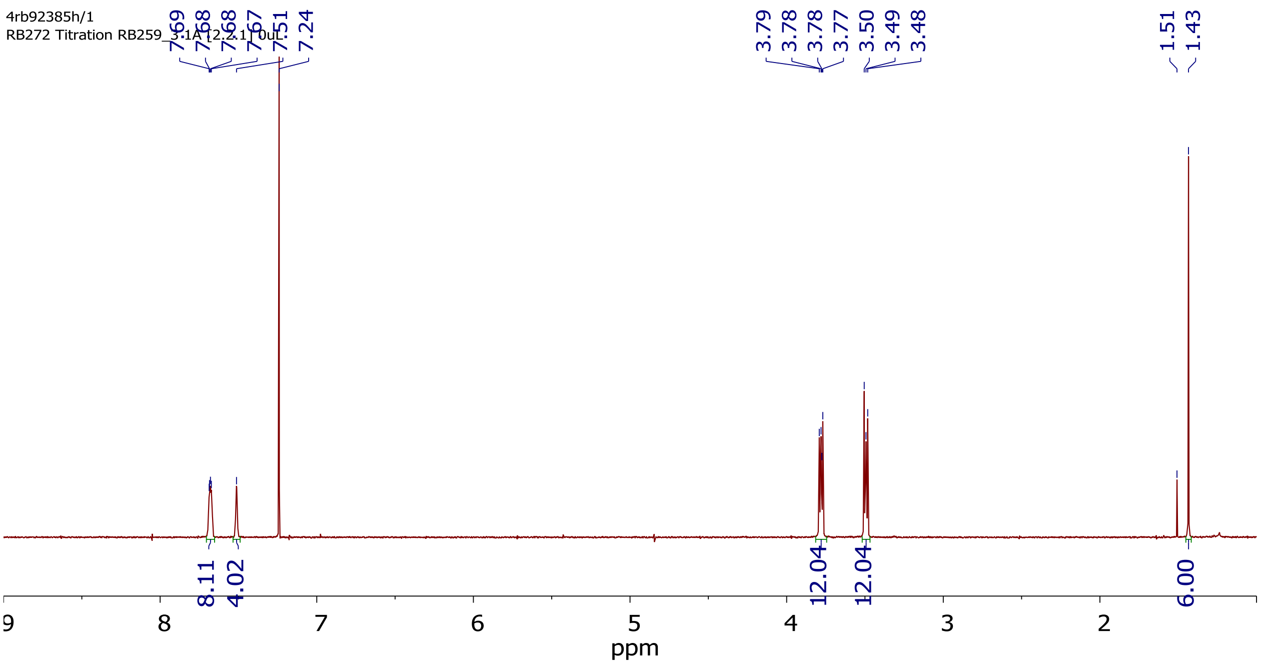

Supplementary Figure 4 | ^{1}H NMR spectrum of [Na^{+}⊂*o*-Me$_2$-1.1.1]BArF^{-}. (400 MHz, $CDCl_3$, 298 K)**.**

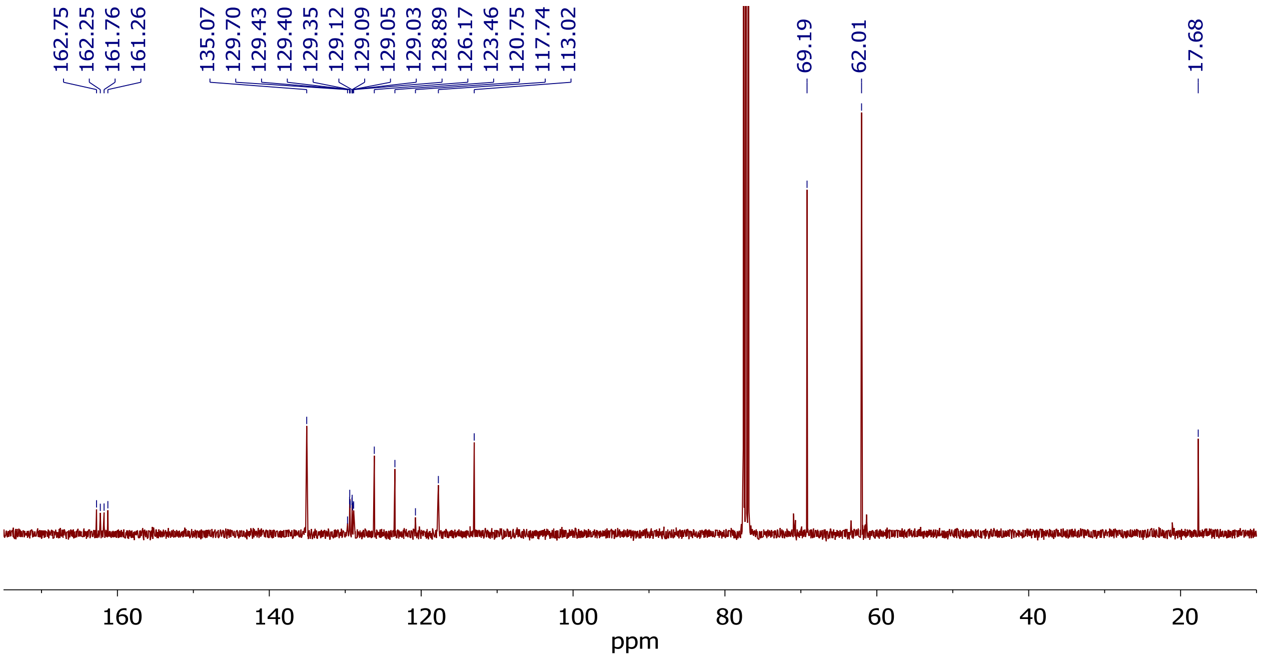

Supplementary Figure 5 | ^{13}C NMR spectrum of [Na^{+}⊂*o*-Me$_2$-1.1.1]BArF^{-}. (100 MHz, $CDCl_3$, 298 K)**.**

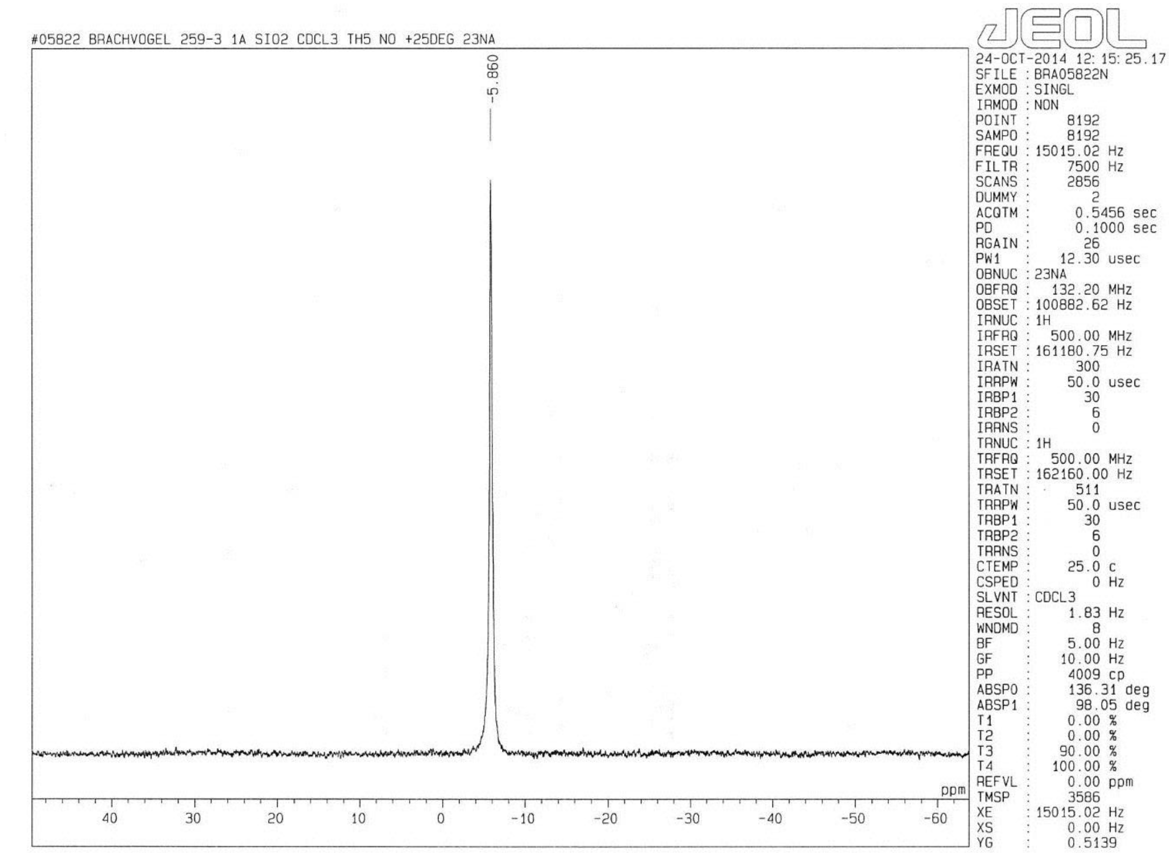

Supplementary Figure 6 | ^{23}Na NMR spectrum of [Na^{+}⊂*o*-Me$_2$-1.1.1]BArF^{-}. (123 MHz, CDCl$_3$, 298 K)**.**

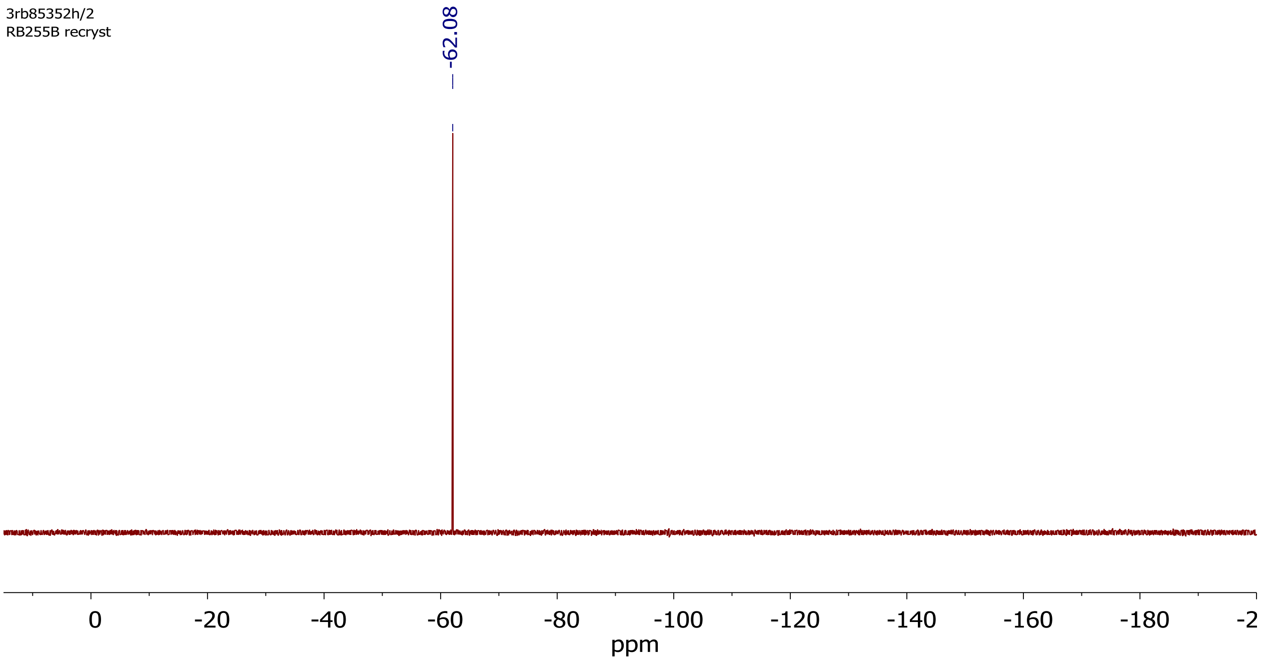

Supplementary Figure 7 | ^{19}F NMR spectrum of [Na^{+}⊂*o*-Me$_2$-1.1.1]BArF^{-}. (282 MHz, CDCl$_3$, 298 K)**.**

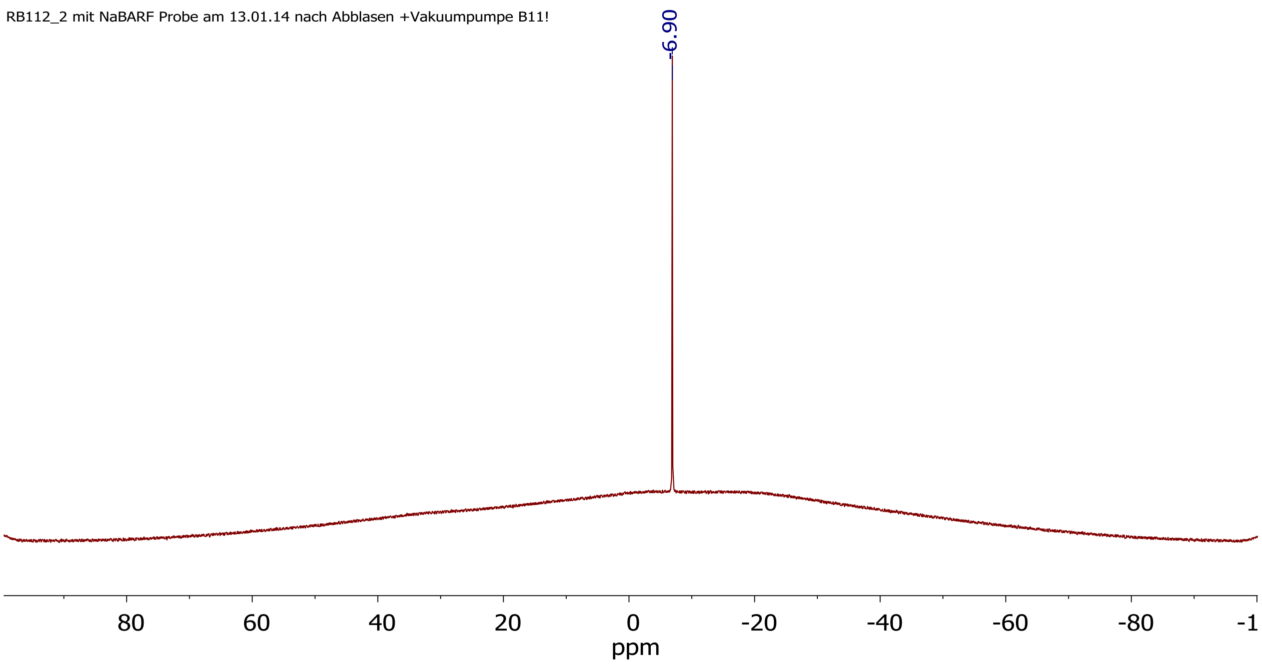

Supplementary Figure 8 | ^{11}B NMR spectrum of [Na^{+}⊂*o*-Me$_2$-1.1.1]BArF^{-}. (128 MHz, $CDCl_3$, 298 K)**.**

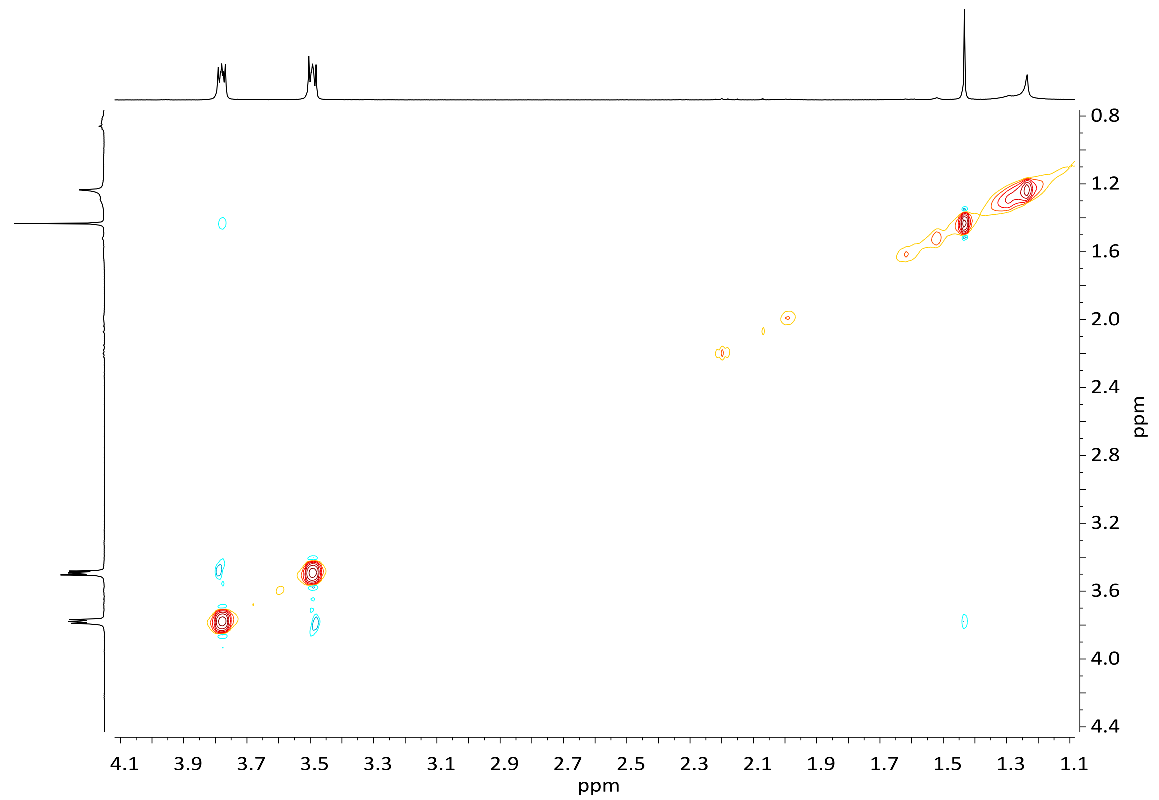

Supplementary Figure 9 | 2D-^{1}H/^{1}H-NOESY-NMR spectrum of [Na$^+\subset$*o*-Me$_2$-1.1.1]BArF$^-$. (400 MHz, $CDCl_3$, 298 K)**.**

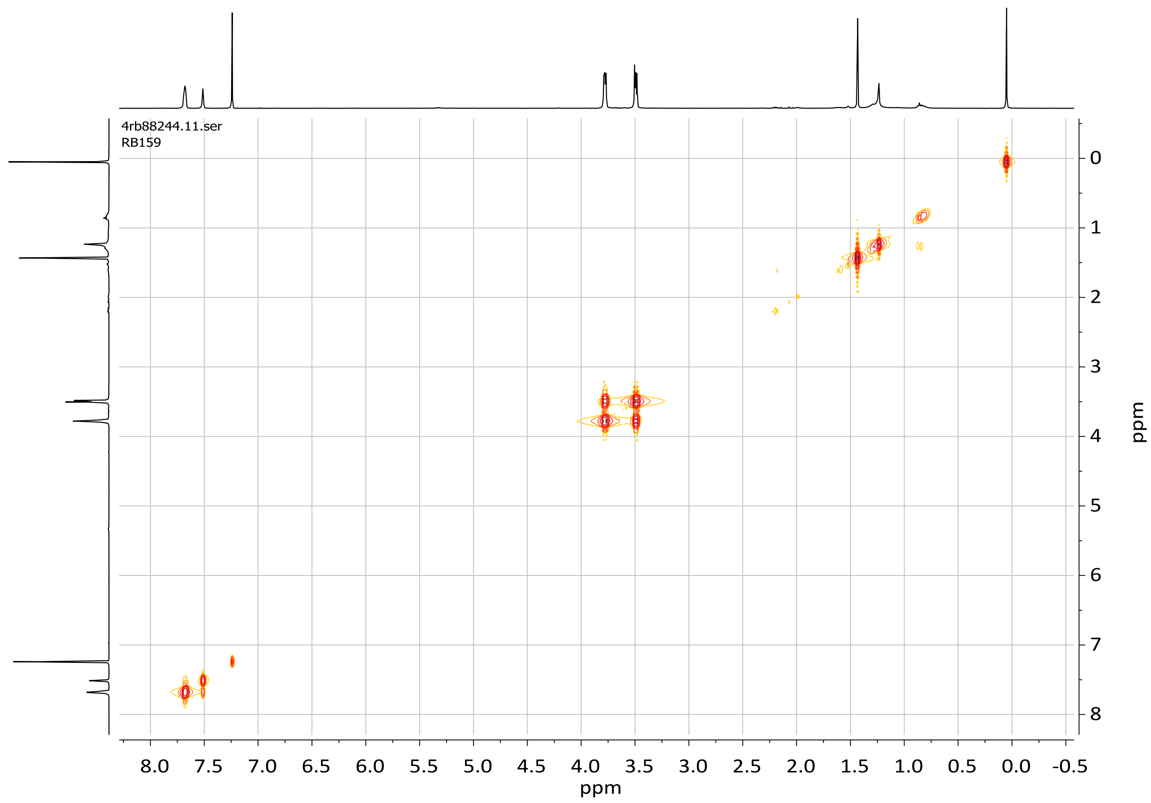

Supplementary Figure 10 | 2D-^{1}H/^{1}H-COSY-NMR spectrum of [Na$^+\subset$*o*-Me$_2$-1.1.1]BArF$^-$. (400 MHz, $CDCl_3$, 298 K)**.**

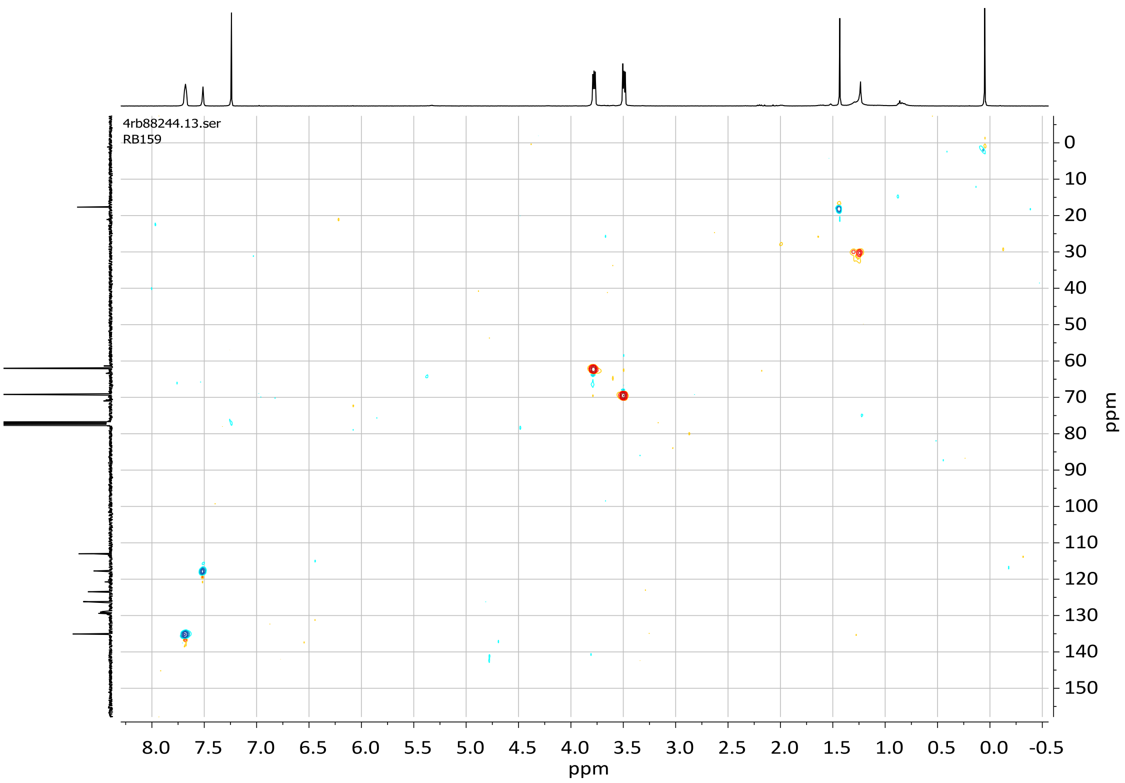

Supplementary Figure 11 | 2D-$^{1}H/^{13}C$-HSQC-NMR spectrum of [Na^{+}⊂*o*-Me_2-1.1.1]$BArF^{-}$. (CDCl$_3$, 298 K).

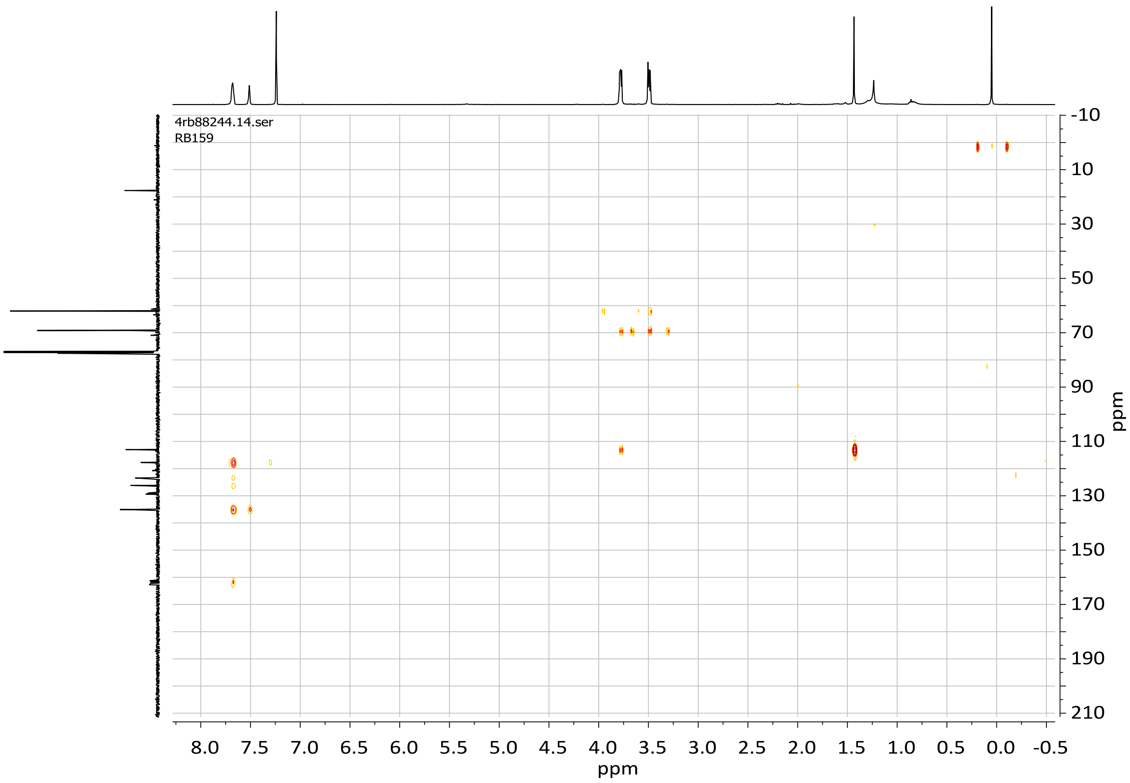

Supplementary Figure 12 | 2D-$^{1}H/^{13}C$-HMBC-NMR spectrum of [Na^{+}⊂*o*-Me_2-1.1.1]$BArF^{-}$. (CDCl$_3$, 298 K).

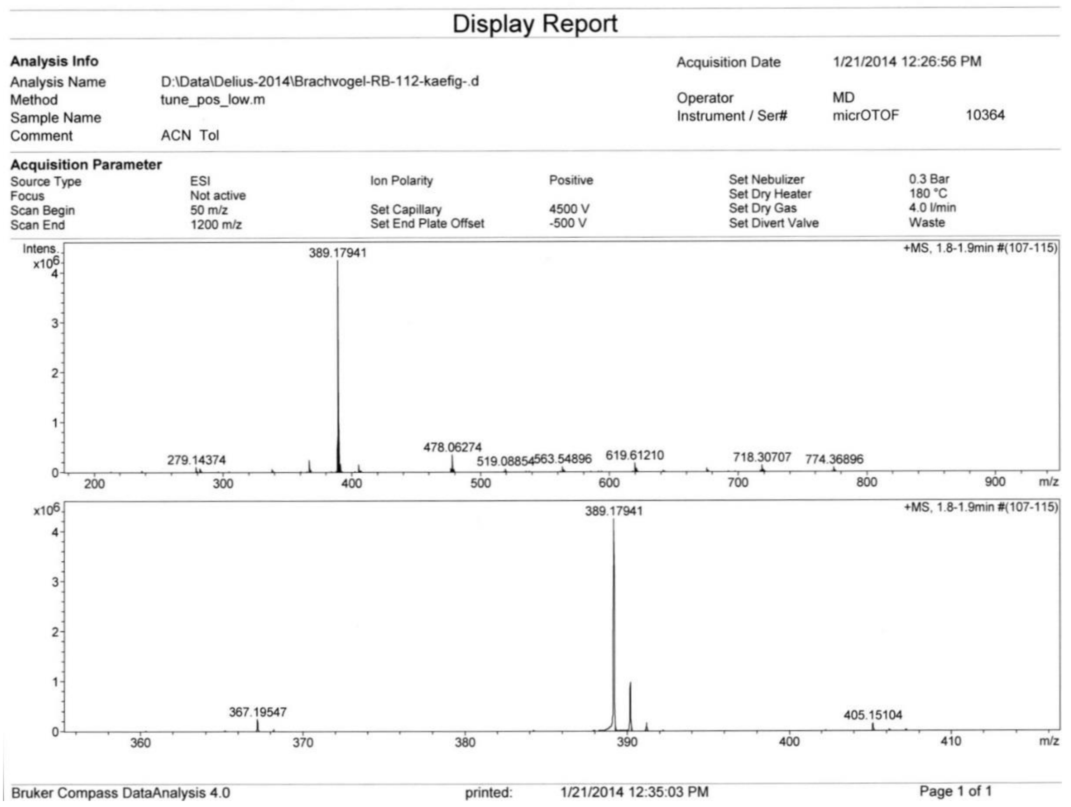

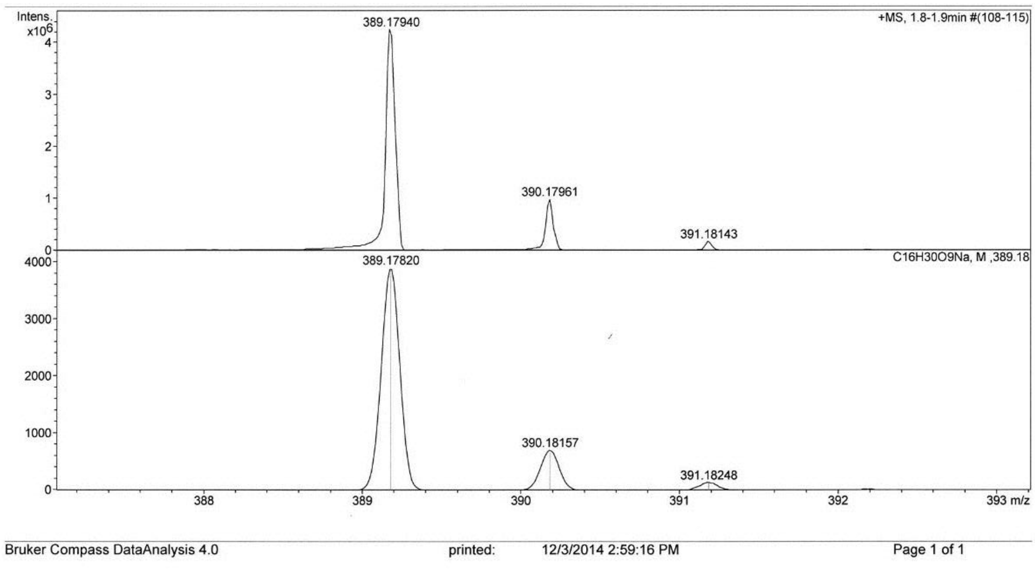

Supplementary Figure 13 | HighRes-MS-Spectrum (ESI$^+$) of [Na$^+$⊂*o*-Me$_2$-1.1.1]BArF$^-$.

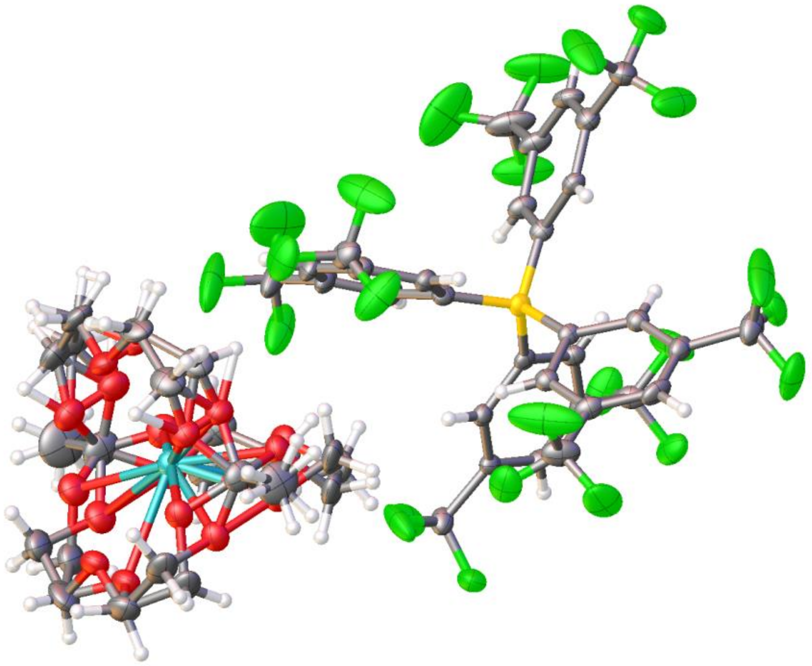

Supplementary Figure 14 | Crystal structure of [Na$^+$⊂*o*-Me$_2$-1.1.1]BArF$^-$. [Na$^+$⊂*o*-Me$_2$-1.1.1]BArF$^-$ was crystallized by slow diffusion of cyclopentane into a solution of **[Na$^+$⊂*o*-Me$_2$-1.1.1]BArF$^-$** into dichloromethane. The cif-file was deposited in the Cambridge structural database under identifier CCDC 1038394.

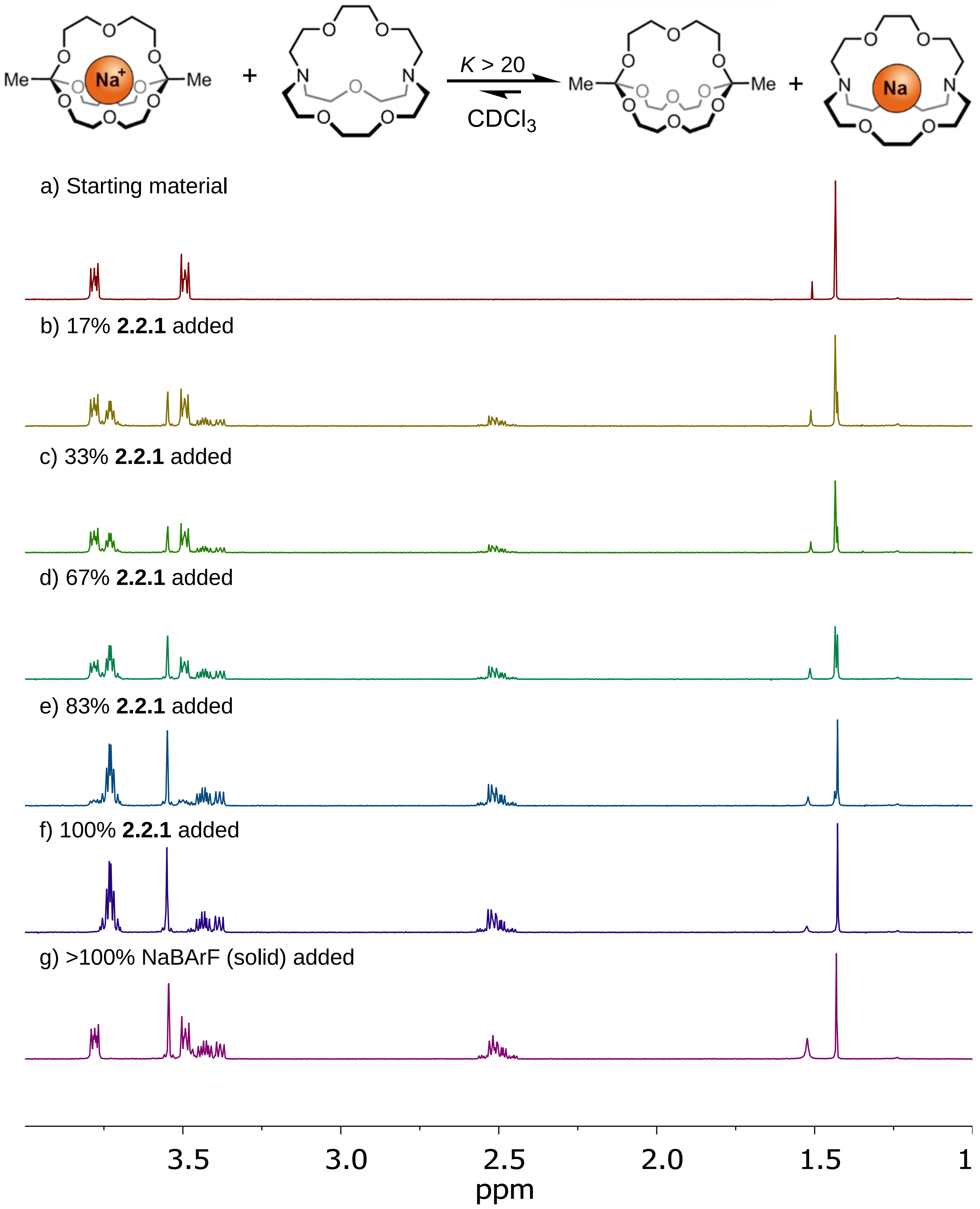

Supplementary Figure 15 | Titration of [Na^+⊂*o*-Me_2-1.1.1]$BArF^-$ with [2.2.1] in $CDCl_3$. ^{1}H NMR spectra (400 MHz, $CDCl_3$, 298 K). Slow exchange on the NMR timescale. $K > 20$ can be deduced for this competition experiment, because at equimolar addition not even traces of [**Na^+⊂*o*-Me_2-1.1.1]$BArF^-$** can be detected.

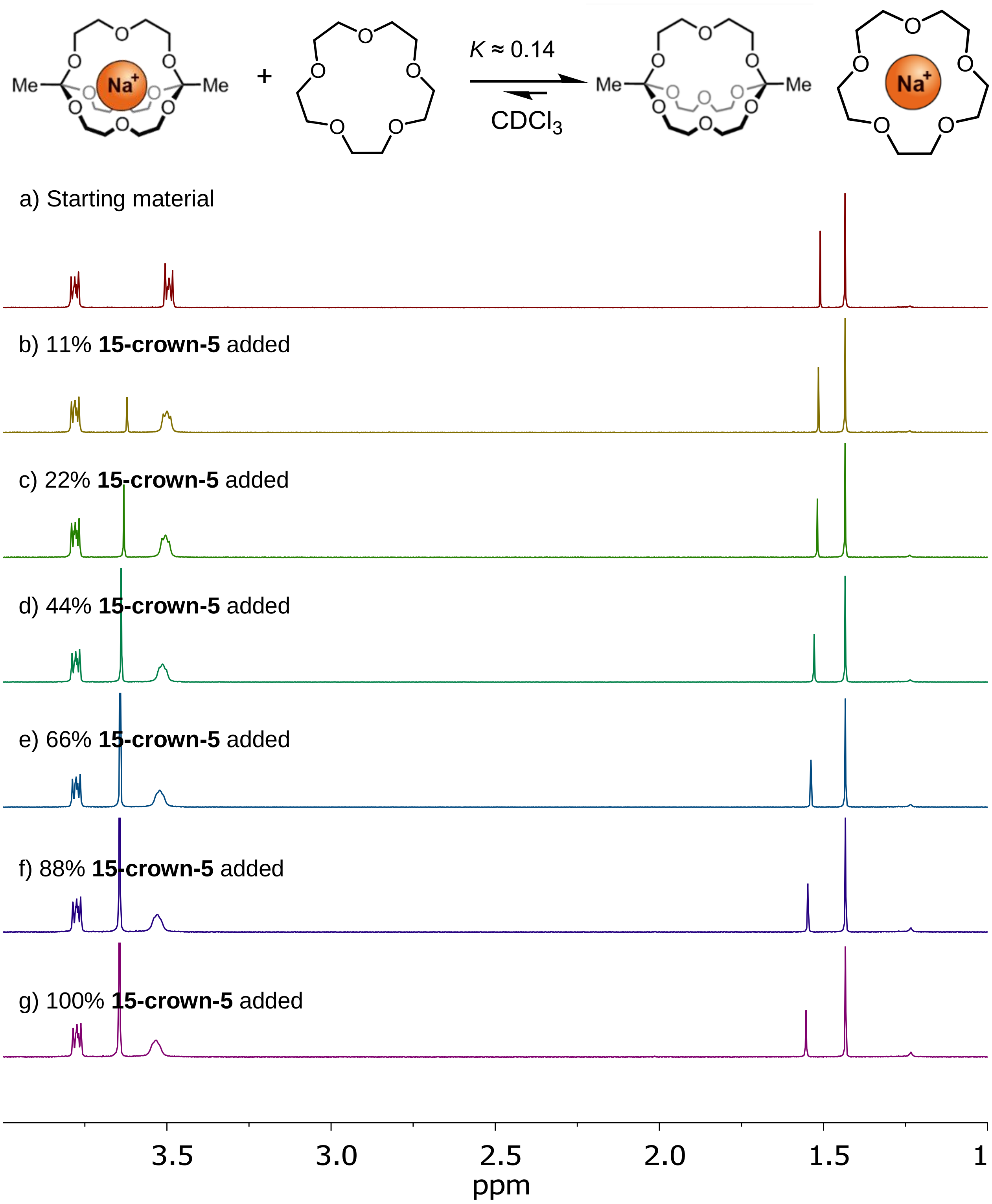

Supplementary Figure 16 | Titration of [Na^+⊂*o*-Me_2-1.1.1]$BArF^-$ with 15-crown-5 in $CDCl_3$. ^1H NMR spectrum (400 MHz, $CDCl_3$, 298 K). Conclusion: Fast exchange on the NMR timescale. The equilibrium constant *K* for this competition experiment is evidently smaller than 1 (see Supplementary Figure 9-10).

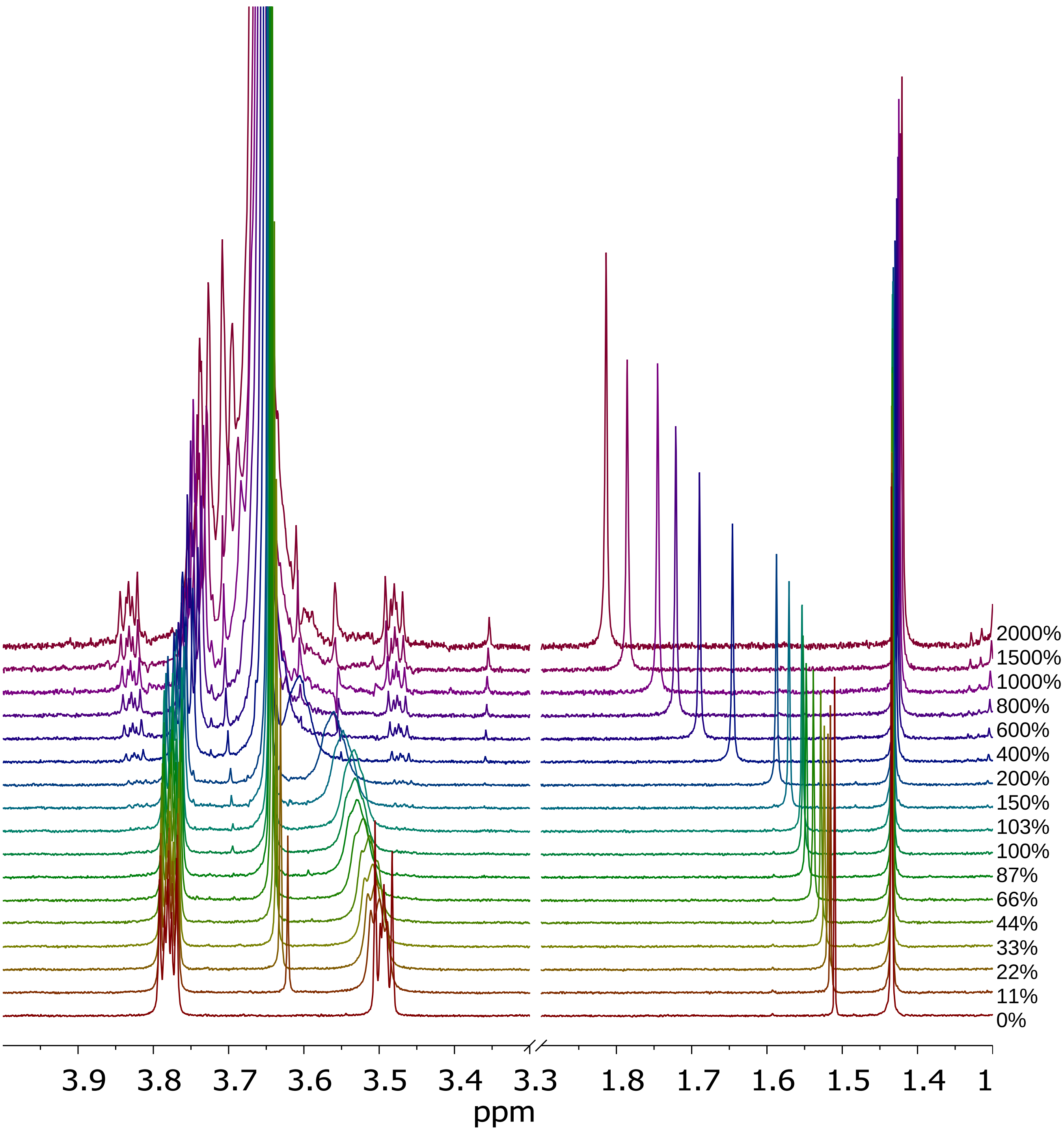

Supplementary Figure 17 | Titration of [Na$^+$⊂*o*-Me$_2$-1.1.1]BArF$^-$ with 15-crown-5 in CDCl$_3$. ^{1}H NMR spectrum (400 MHz, CDCl$_3$, 298 K). NMR spectra resulting from titration with up to 20 equiv. 15-crown-5**.**

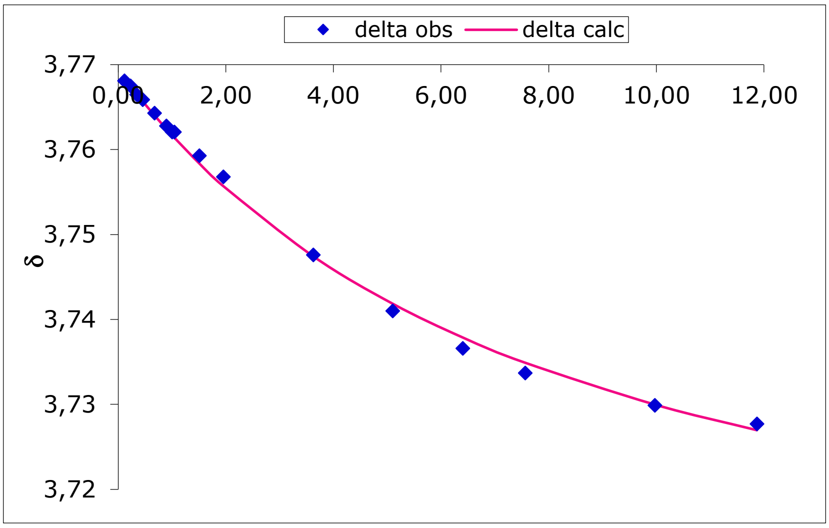

Supplementary Figure 18 | NMR isotherm and fit of titration of [Na$^+$⊂*o*-Me$_2$-1.1.1]BArF$^-$ with 15-crown-5 in CDCl$_3$. K = 0.14 ± 0.01. Fit of NMR data (program NMR_Fit_HG)[1] suggests that the equilibrium constant for this competition experiment (K) is approximately 0.14 (standard deviation ±0.01 for two measurements; we would estimate that the experimental error is at ±0.05, e.g. due to variation of water content), hence it can be assumed that under these conditions ***o*-Me$_2$-1.1.1** binds Na$^+$ with a K_A that is approximately one order of magnitude larger than for **15-crown-5**.

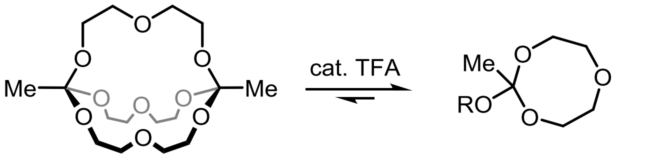

***o*-Me$_2$-1.1.1**
+ [Na$^+$⊂2.2.1]BArF$^-$

+ [Na$^+$⊂2.2.1]BArF$^-$

a)

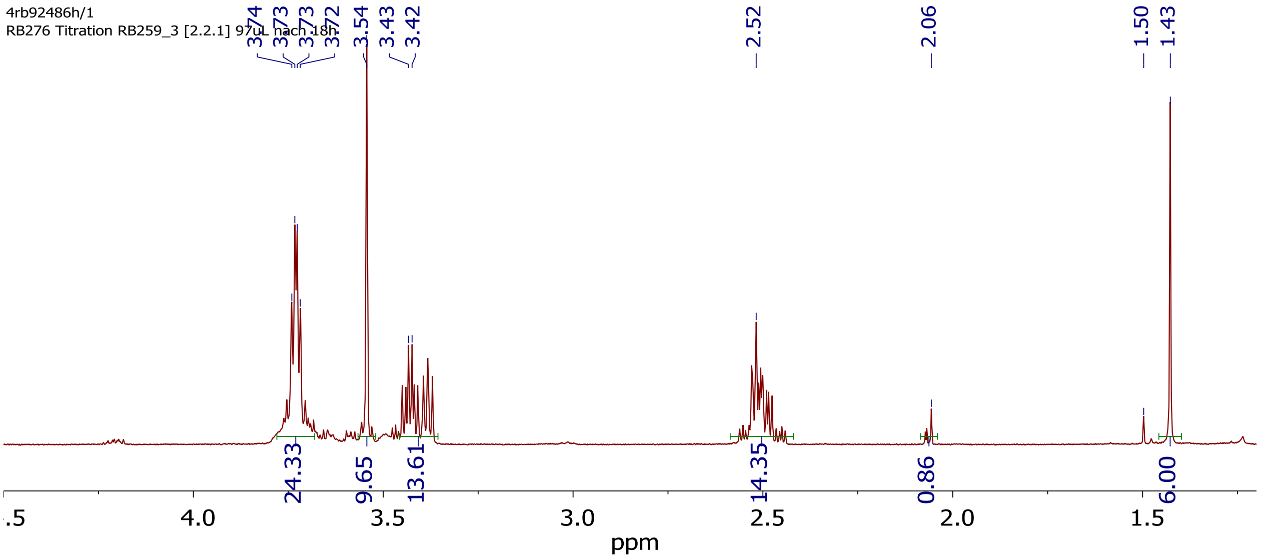

b)

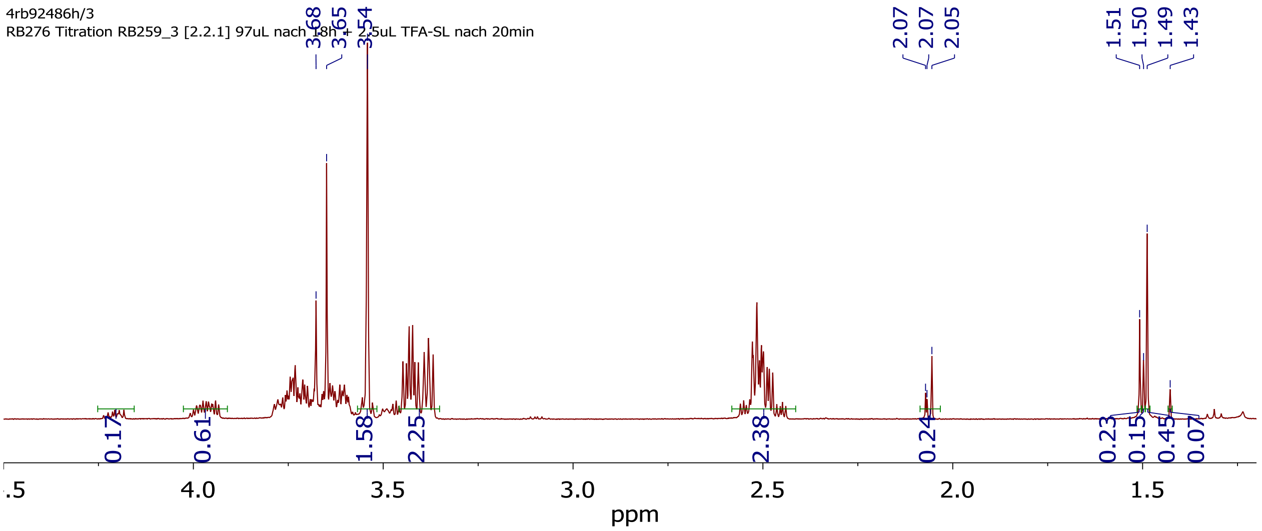

Supplementary Figure 19 | Addition of cat. TFA to 1:1 mixture of *o*-Me$_2$-1.1.1 and [Na$^+$⊂2.2.1]BArF$^-$. a) ^{1}H NMR spectrum (400 MHz, $CDCl_3$, 298 K) **before** TFA addition. **b)** ^{1}H NMR spectrum (400 MHz, $CDCl_3$, 298 K) **after** addition of 1% TFA. Conclusion: ***o*-Me$_2$-1.1.1** is almost completely transformed into other orthoester species with chemical shifts indicative for 8-membered ring products (CH_3 group at >1.48 ppm and CH_2 groups at 4.0 ppm).

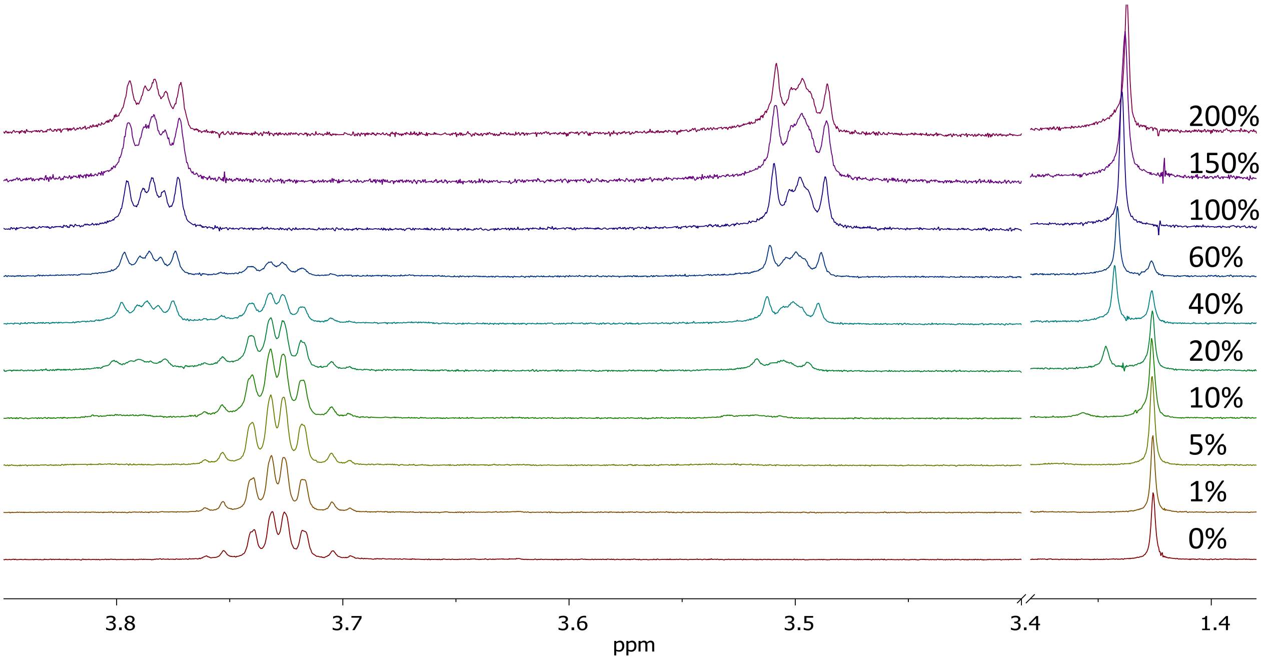

Supplementary Figure 20 | Determination of K_A of [Na^+⊂*o*-Me_2-1.1.1]$BArF^-$ in $CDCl_3$. ^1H NMR spectra (400 MHz, $CDCl_3$, 298 K). NaBArF was added to a 1.0 mM solution of ***o*-Me_2-1.1.1** in $CDCl_3$ (satd. with D_2O). Due to slow exchange on the NMR time scale (generally indicative of high binding constants), the determination of K_A was not possible in this solvent.

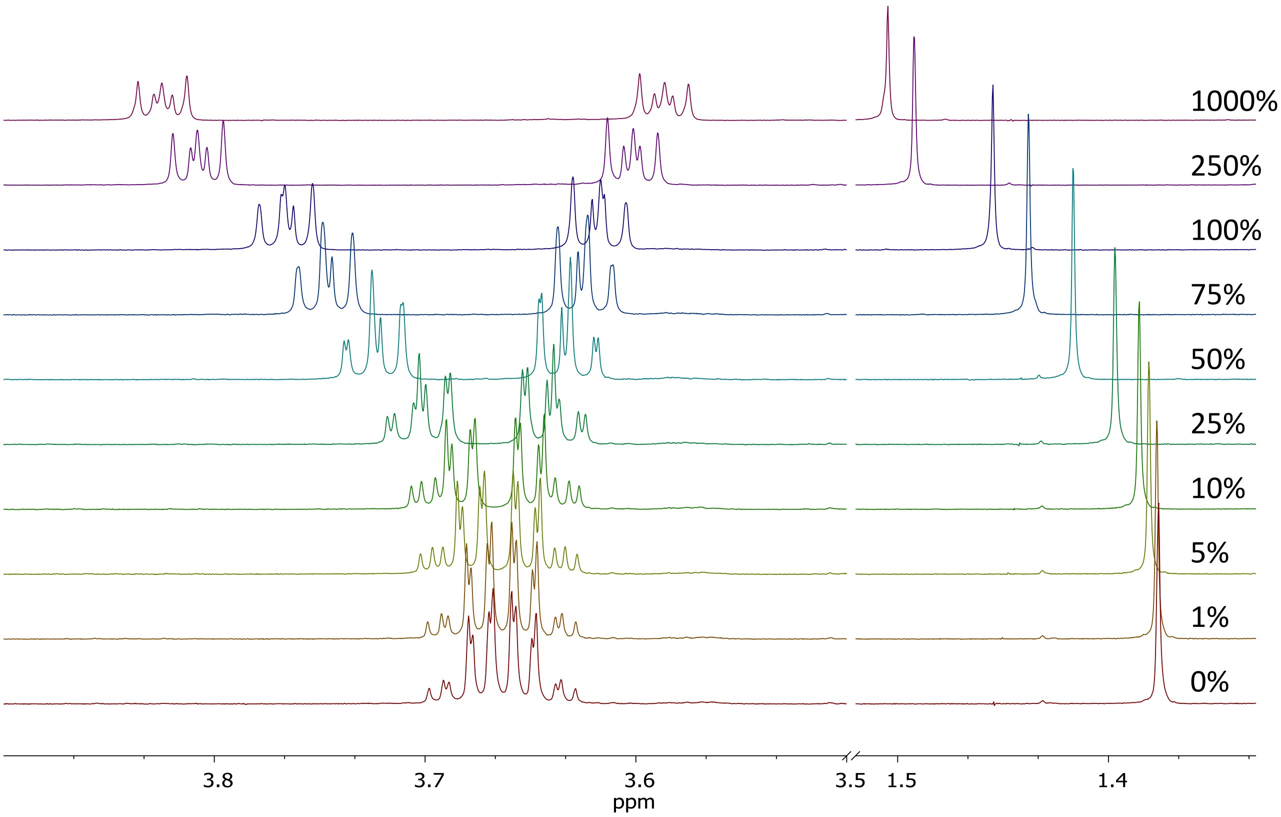

Supplementary Figure 21 | Determination of K_A of $[Na^+ \subset$*o*-Me_2-1.1.1]BArF$^-$ in MeCN-d_3. ^1H NMR spectra (400 MHz, $CDCl_3$, 298 K). A 90 mM solution of NaBArF was added to a 3.0 mM solution of ***o*-Me_2-1.1.1** in MeCN-d_3.

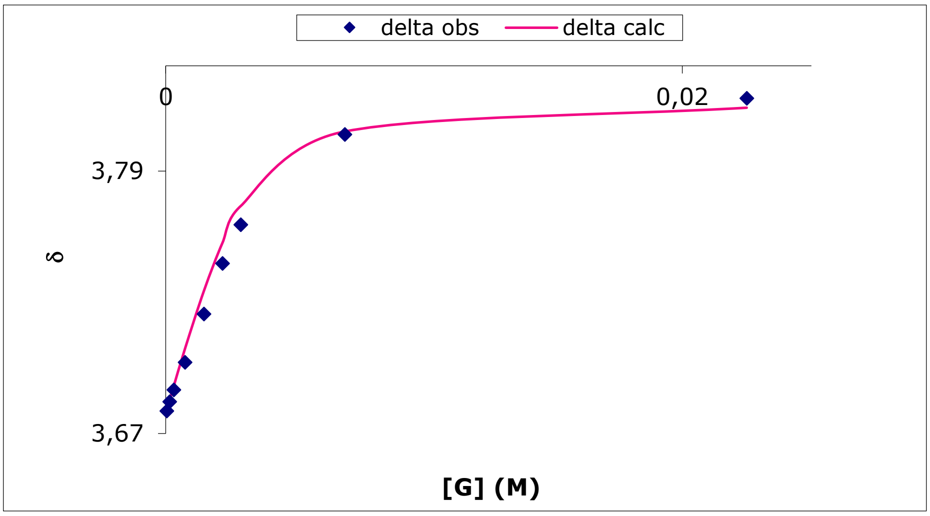

Supplementary Figure 22 | NMR binding isotherm and fit of titration of *o*-Me_2-1.1.1 with NaBArF in MeCN-d_3. (^1H NMR, 400 MHz, $CDCl_3$, 298 K). A 90 mM solution of NaBArF was added to a 3.0 mM solution of ***o*-Me_2-1.1.1** in MeCN-d_3. Fit of NMR data **(program NMR_Fit_HG)[1]** results in a K_A **= 2087 ± 103 M^{-1} (standard devation based on 4 measurement points).**

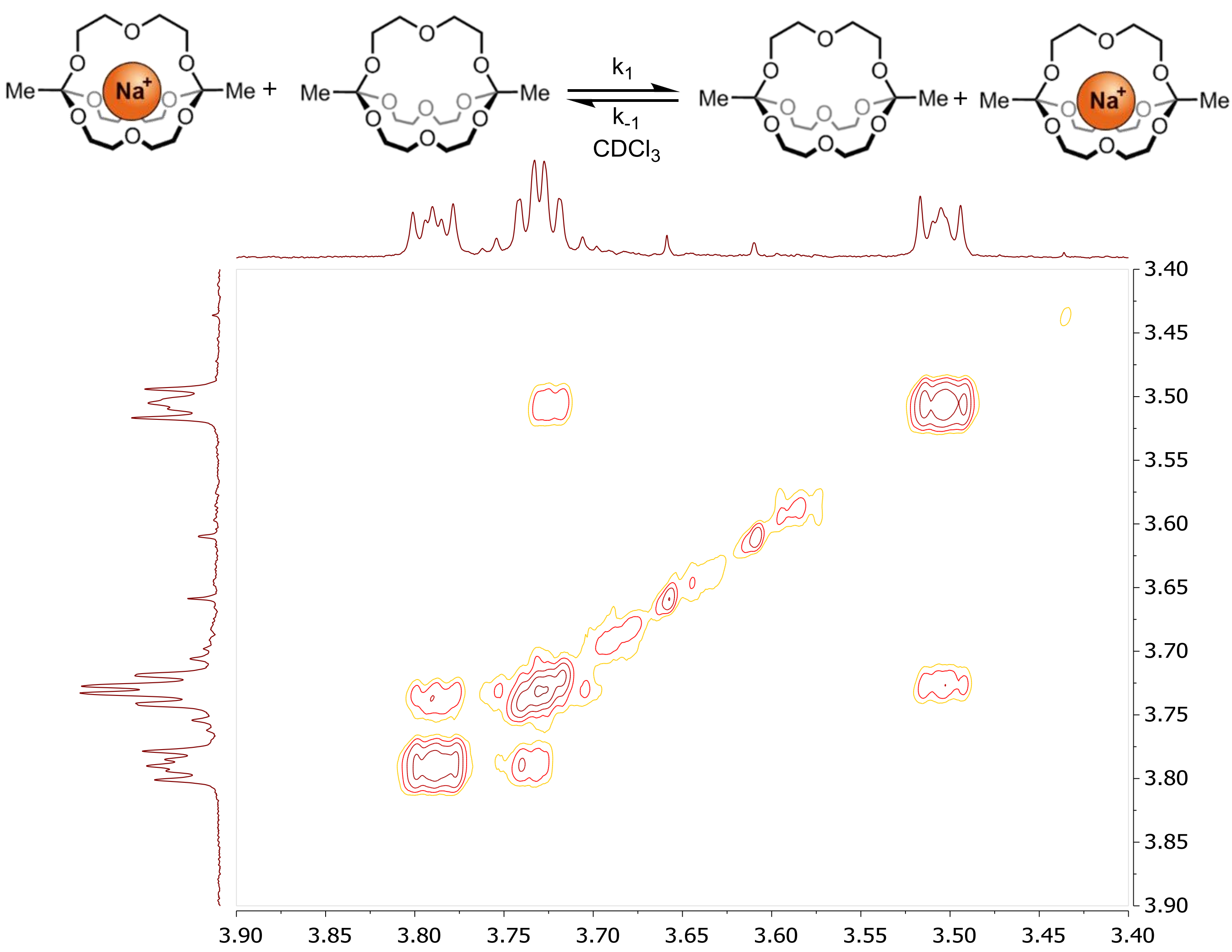

Supplementary Figure 23 | EXSY NMR. Partial 2D EXSY NMR spectrum ($CDCl_3$, 298 K, 400 MHZ, mixing time τ_m = 500 ms). The mixture of [**Na$^+$⊂*o*-Me$_2$-1.1.1]BArF$^-$** and ***o*-Me$_2$-1.1.1** was prepared as described in Supplementary Methods. Due to the observation of slow exchange on the NMR time scale, the kinetics of sodium exchange between two degenerate orthoester cryptands can be studied by means of EXSY NMR spectroscopy (see Supplementary Note 1).

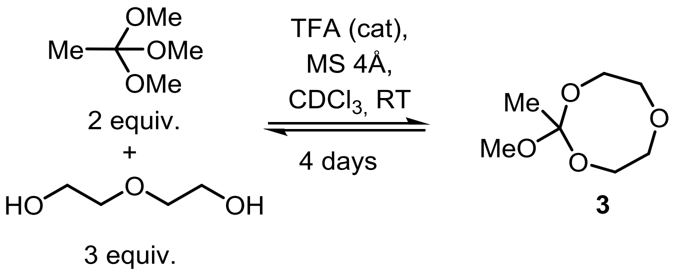

3

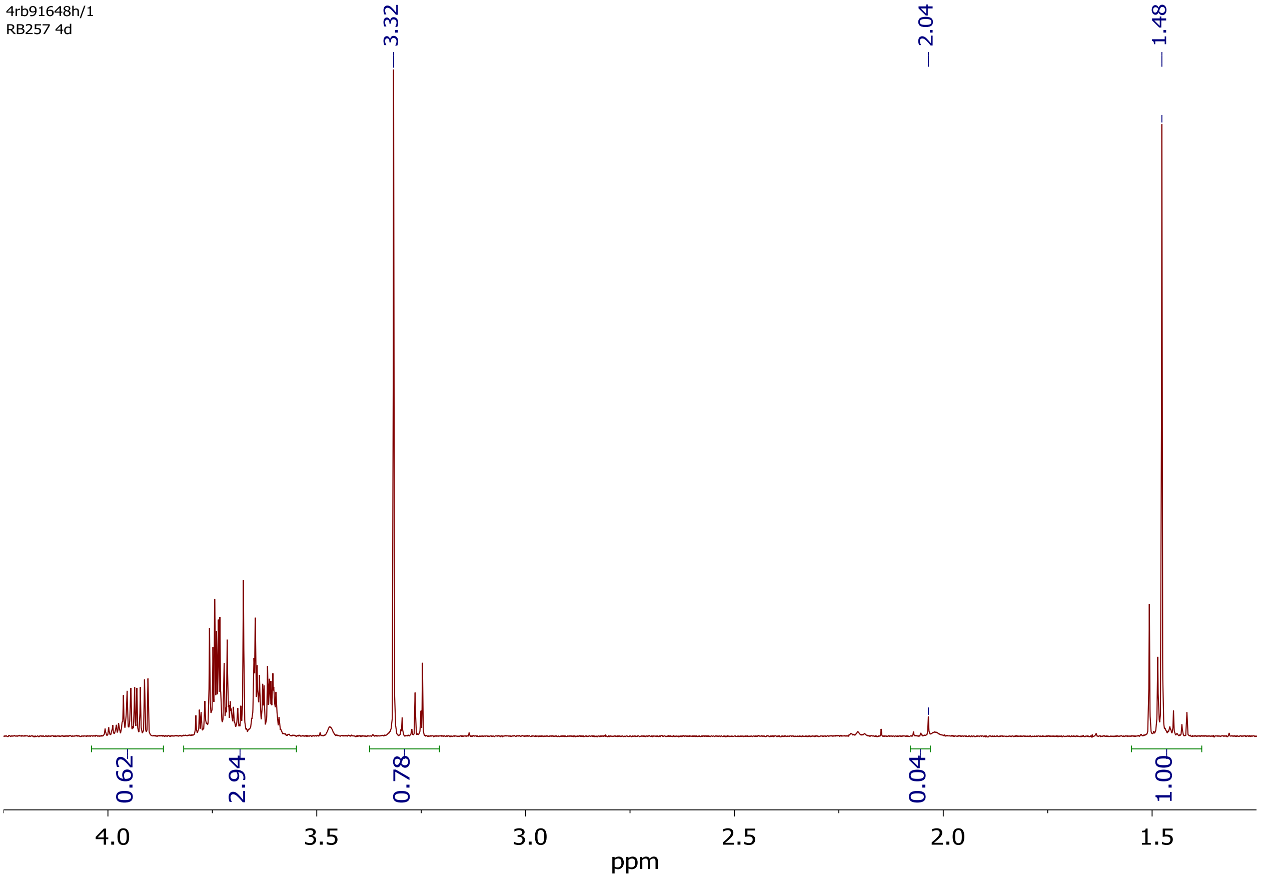

Supplementary Figure 24 | Reaction outcome in the absence of template (NaBArF). ^{1}H NMR spectrum (400 MHz, $CDCl_3$, 298 K) after 4 days (see also Supplementary Methods).

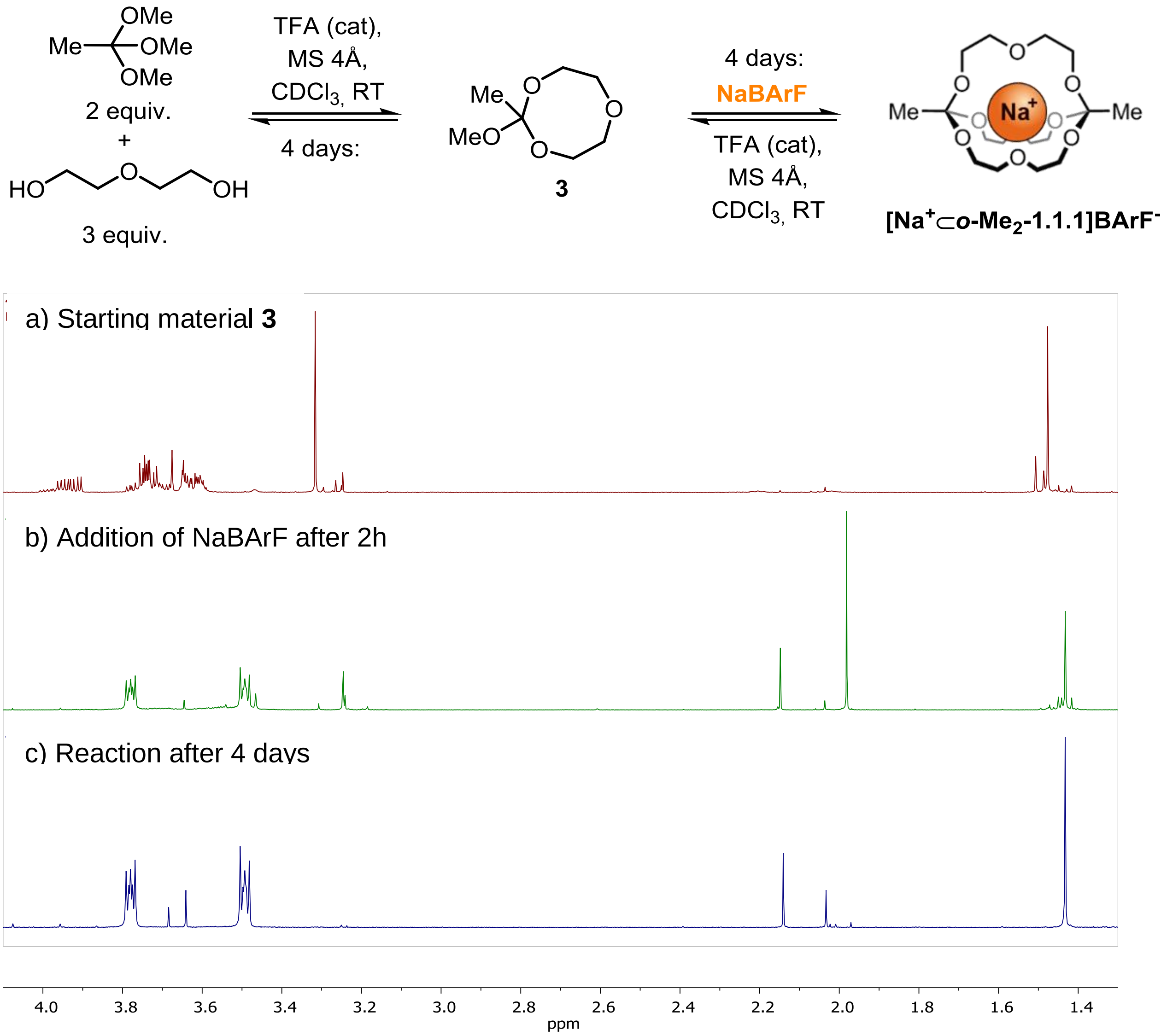

Supplementary Figure 25 | Two-step preparation of [Na^+⊂*o*-Me_2-1.1.1]$BArF^-$ via compound 3 (see also Supplementary Methods). ^{1}H NMR spectrum (400 MHz, $CDCl_3$, 298 K).

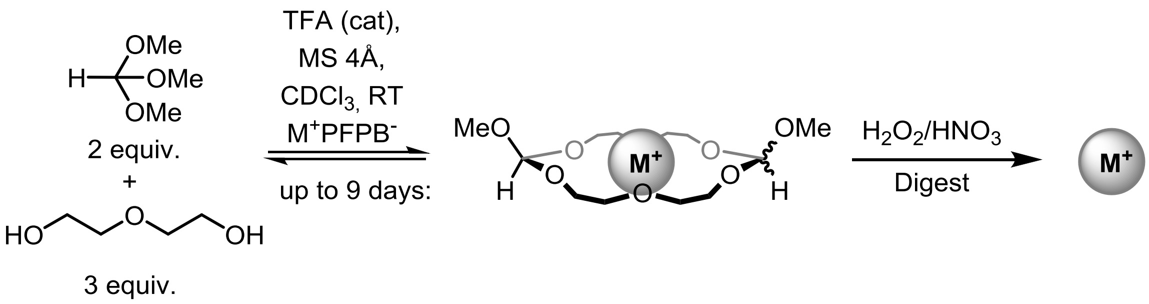

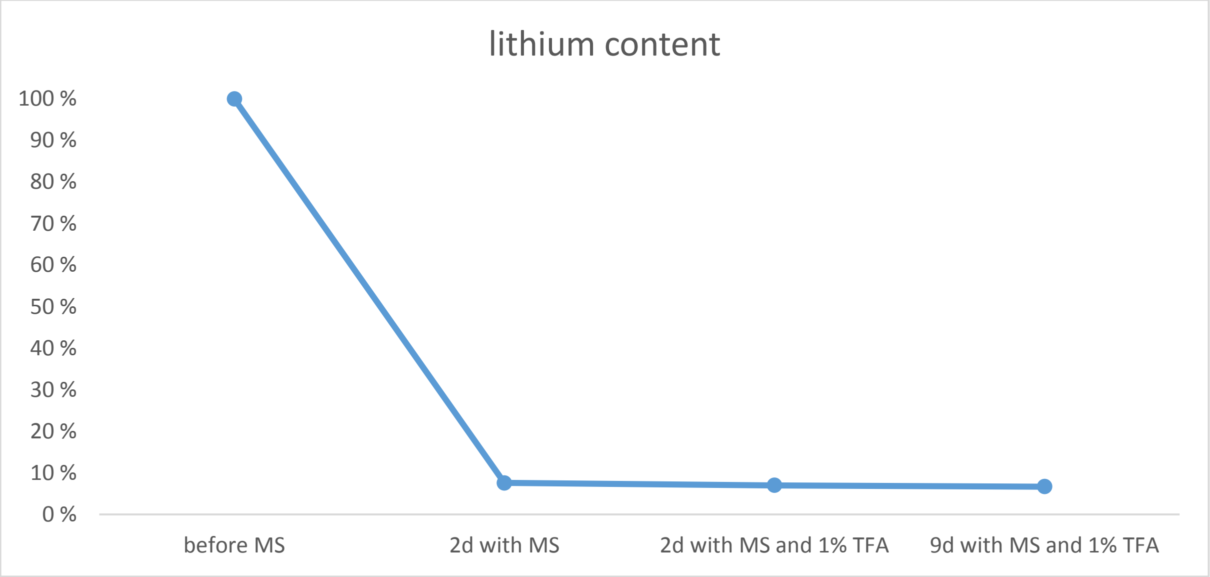

Supplementary Figure 26 | Lithium content measured with Atom Emission Spectroscopy (AES). Zeolite type A (molecular sieves 4Å) is clearly responsible for the exchange of lithium against sodium (which was also observed by ^{1}H, ^{23}Na, ^{7}Li NMR spectroscopy and mass spectrometry)[2]. Acid (TFA) seems to be not necessary for this cation exchange to occur and an equilibrium between Li^{+}/Na^{+} is reached even before orthoester exchange is initiated. The concentration of sodium ions was also measured, but poor quality data was obtained due to the ubiquity of sodium in glassware and reagents. (See also Supporting Table 3 and Supporting Methods).

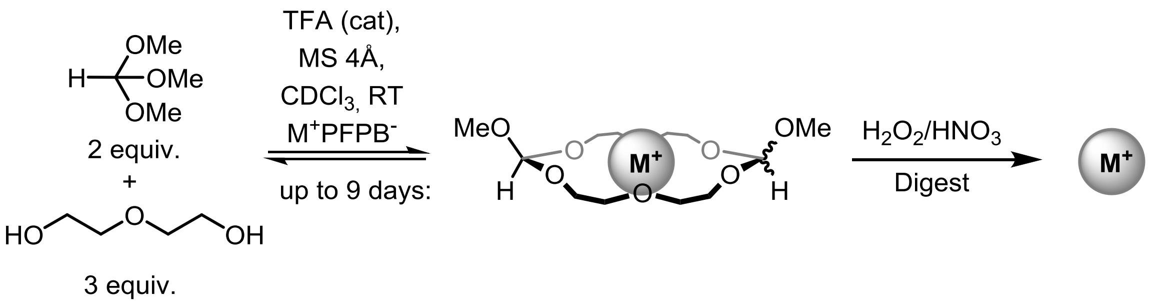

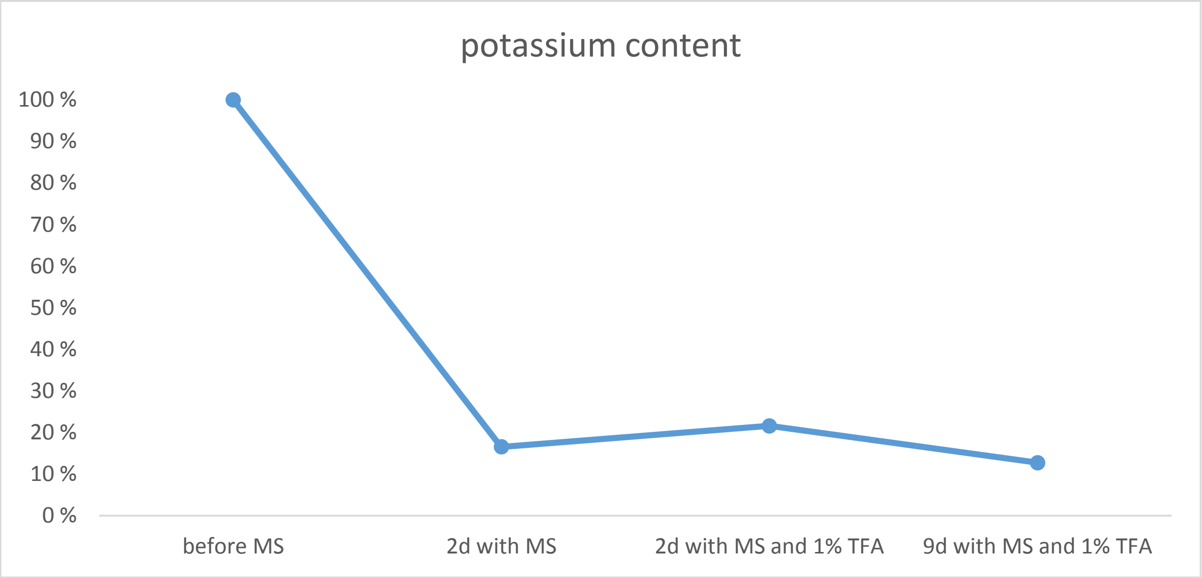

Supplementary Figure 27 | Potassium content measured with Atom Absorption Spectroscopy (AAS). Zeolite type A (molecular sieves 4Å) is clearly responsible for the exchange of potassium against sodium (which was also observed by ^{1}H, ^{23}Na, ^{7}Li NMR spectroscopy and mass spectrometry)[2]. Acid (TFA) seems to be not necessary for this cation exchange to occur and an equilibrium between K^+/Na^+ is reached even before orthoester exchange is initiated. The concentration of sodium ions was also measured, but poor quality data was obtained due to the ubiquity of sodium in glassware and reagents. (See also Supporting Table 3 and Supporting Methods).

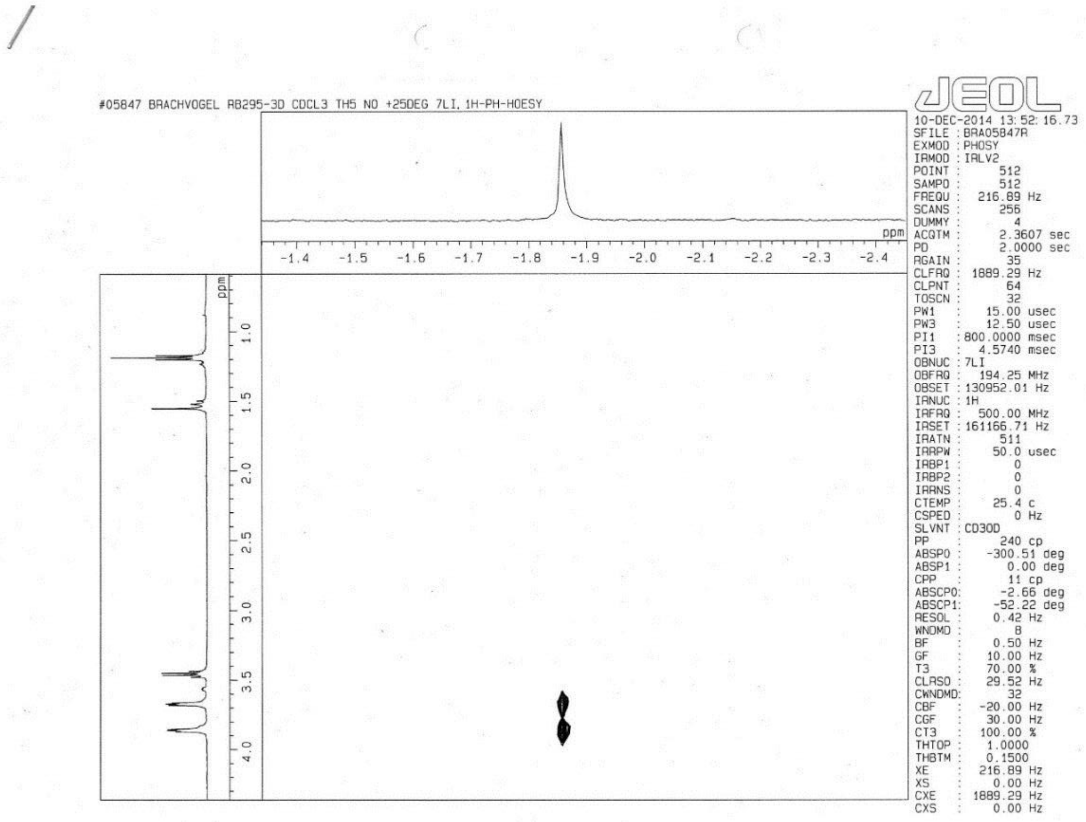

Supplementary Figure 28 | ^{1}H/^{7}Li Hetero-NOE NMR spectrum of [Li$^+$⊂*o*-Me$_2$-1.1.1]TPFPB$^-$. (500 MHz, $CDCl_3$, 298 K). Spectrum confirms presence of Li^+ in cryptand cage (no cross-peaks with co-solvent Et_2O).

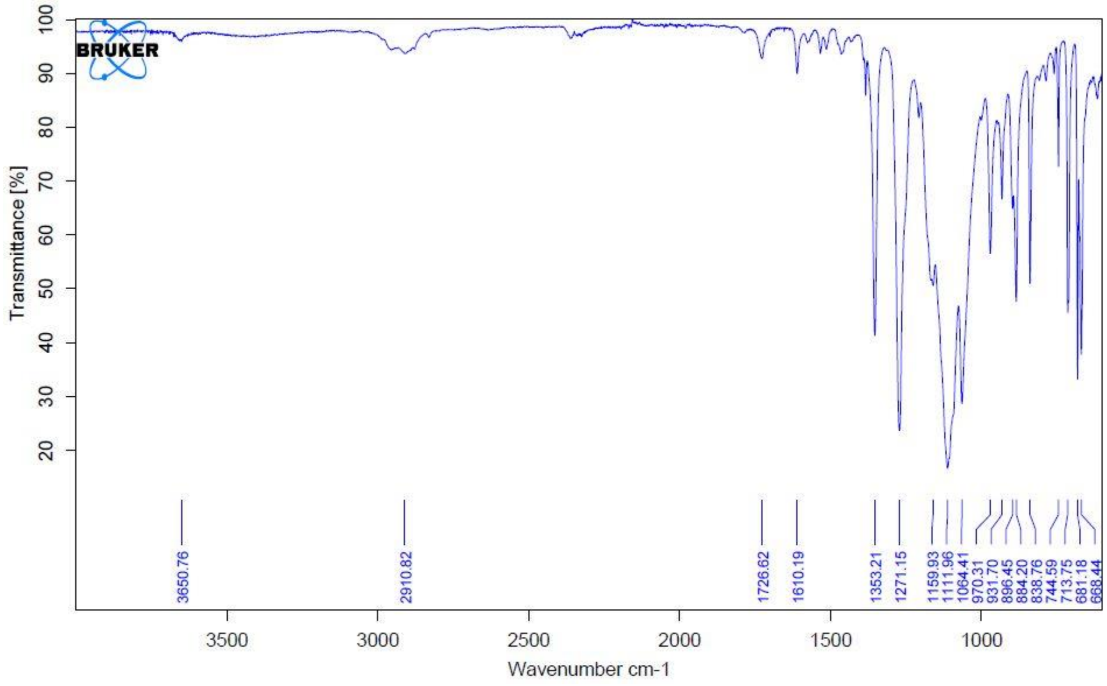

Supplementary Figure 29 | IR spectrum of [Na$^+$⊂*o*-Me$_2$-1.1.1]BArF$^-$. (ATR, RT).

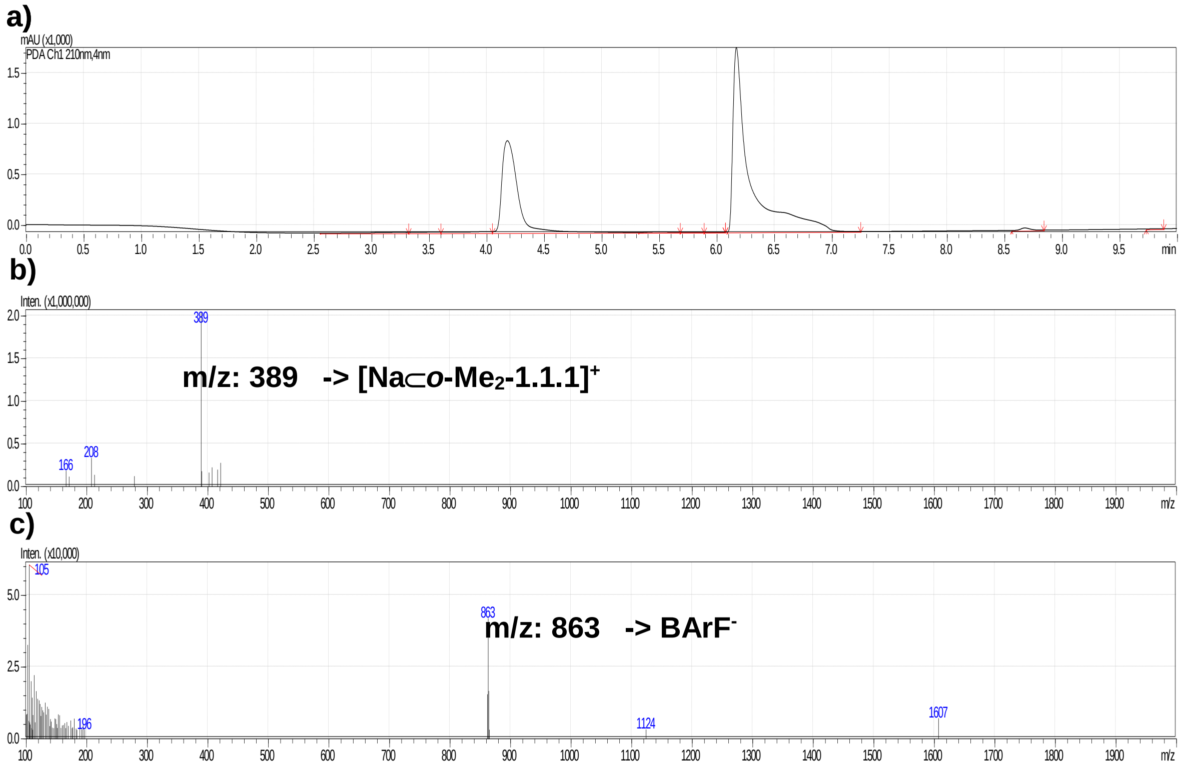

Supplementary Figure 30 | Analysis of [Na$^+$⊂*o*-Me$_2$-1.1.1]BArF$^-$ by reverse-phase HPLC-MS. a) HPLC chromatogram (210 nm). **b)** ESI$^+$ mass spectrum for peak at 4.18 min. **c)** ESI$^-$ mass spectrum for peak at 6.17 min.

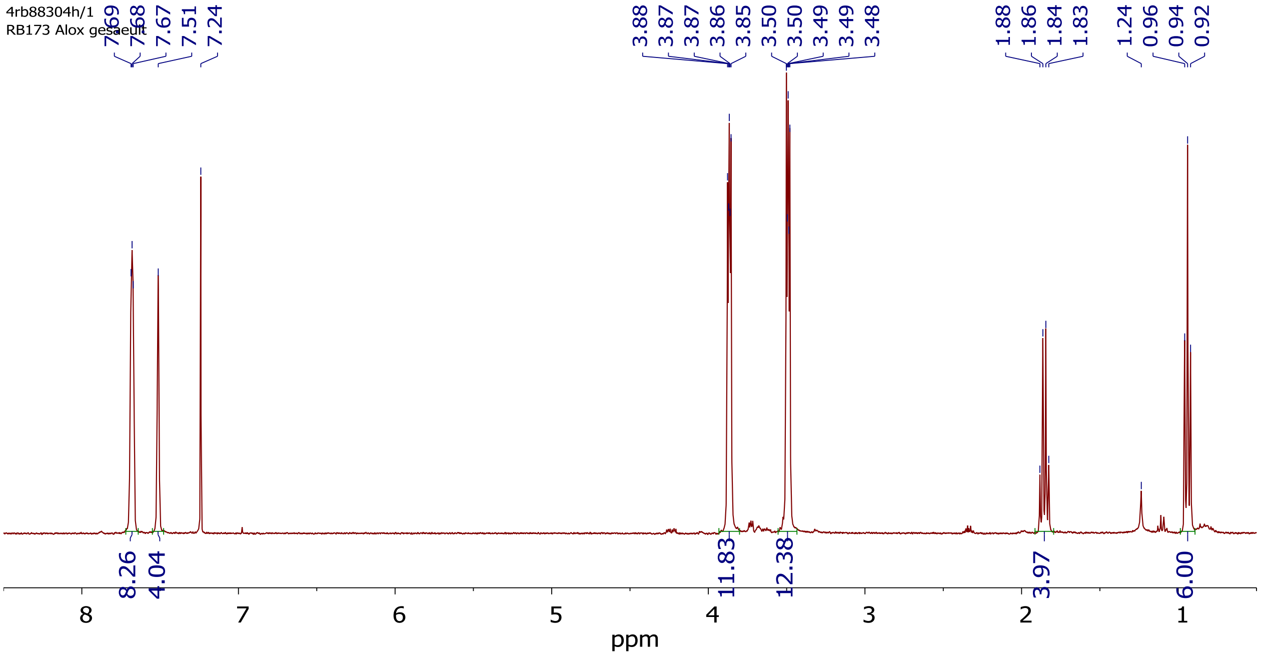

Supplementary Figure 31 | ^{1}H NMR spectrum of [Na^{+}⊂*o*-Et$_2$-1.1.1]BArF^{-}. (400 MHz, CDCl$_3$, 298 K)**.**

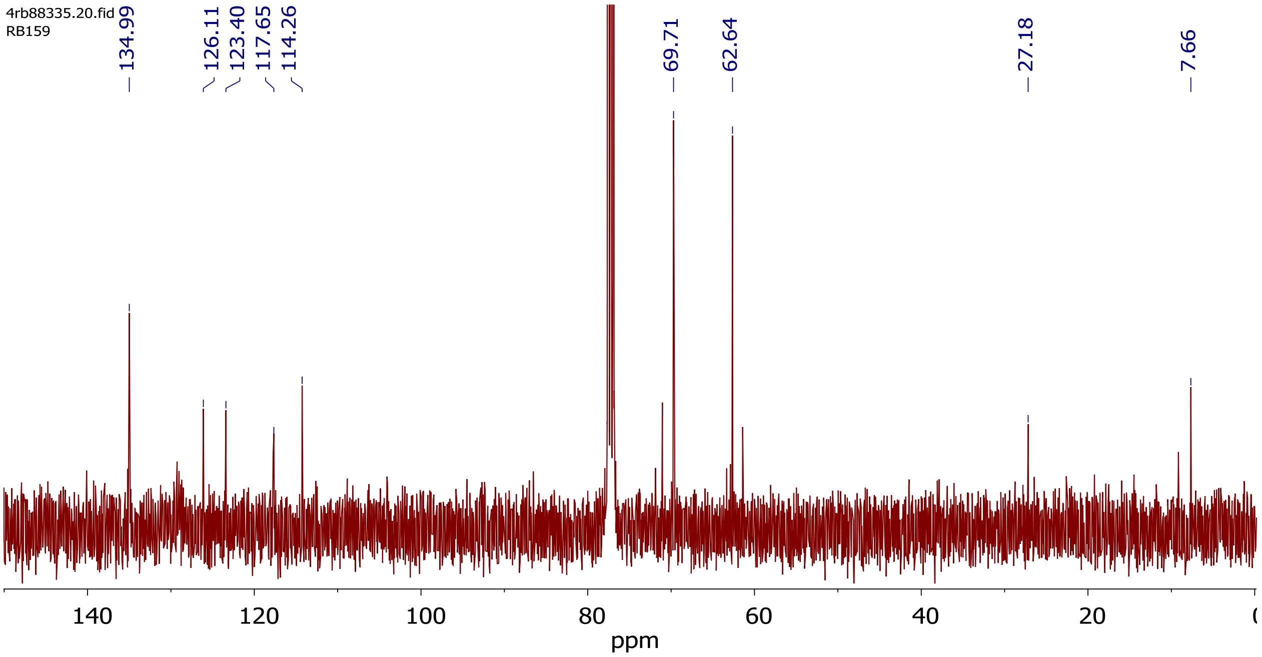

Supplementary Figure 32 | ^{13}C NMR spectrum of [Na^{+}⊂*o*-Et$_2$-1.1.1]BArF^{-}. (100 MHz, CDCl$_3$, 298 K)**.**

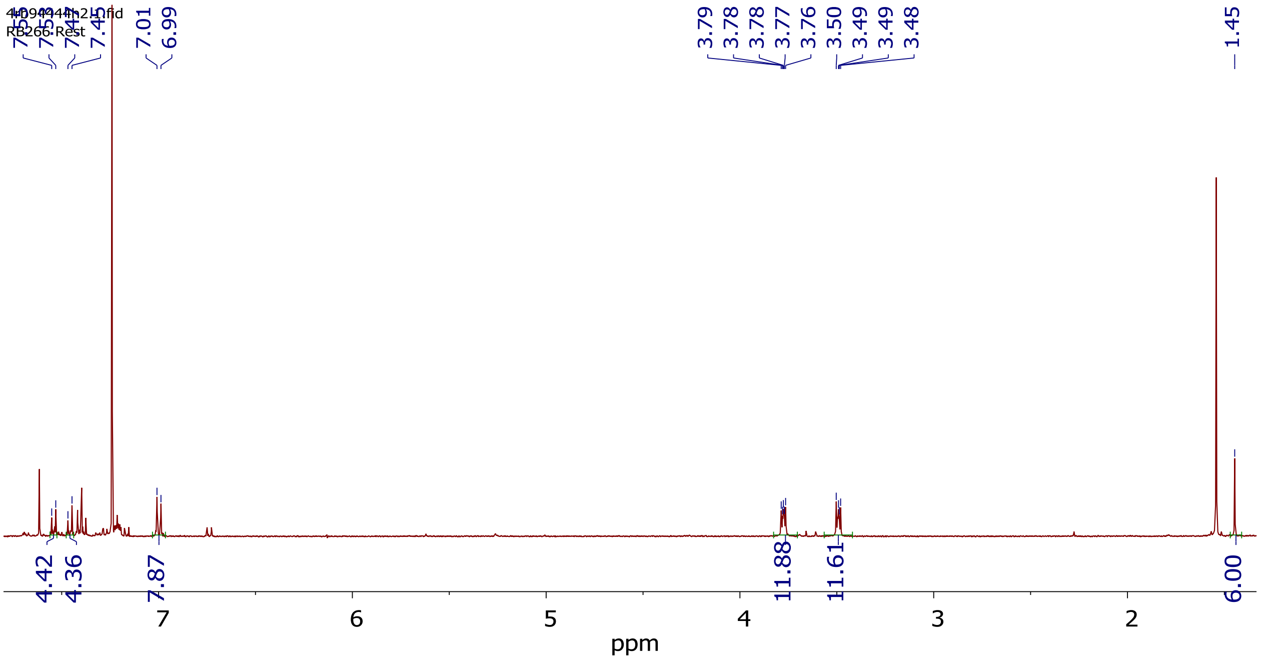

Supplementary Figure 33 | ^{1}H NMR spectrum of [Na^{+}⊂*o*-Me$_2$-1.1.1]TCPB^{-}. (400 MHz, $CDCl_3$, 298 K).

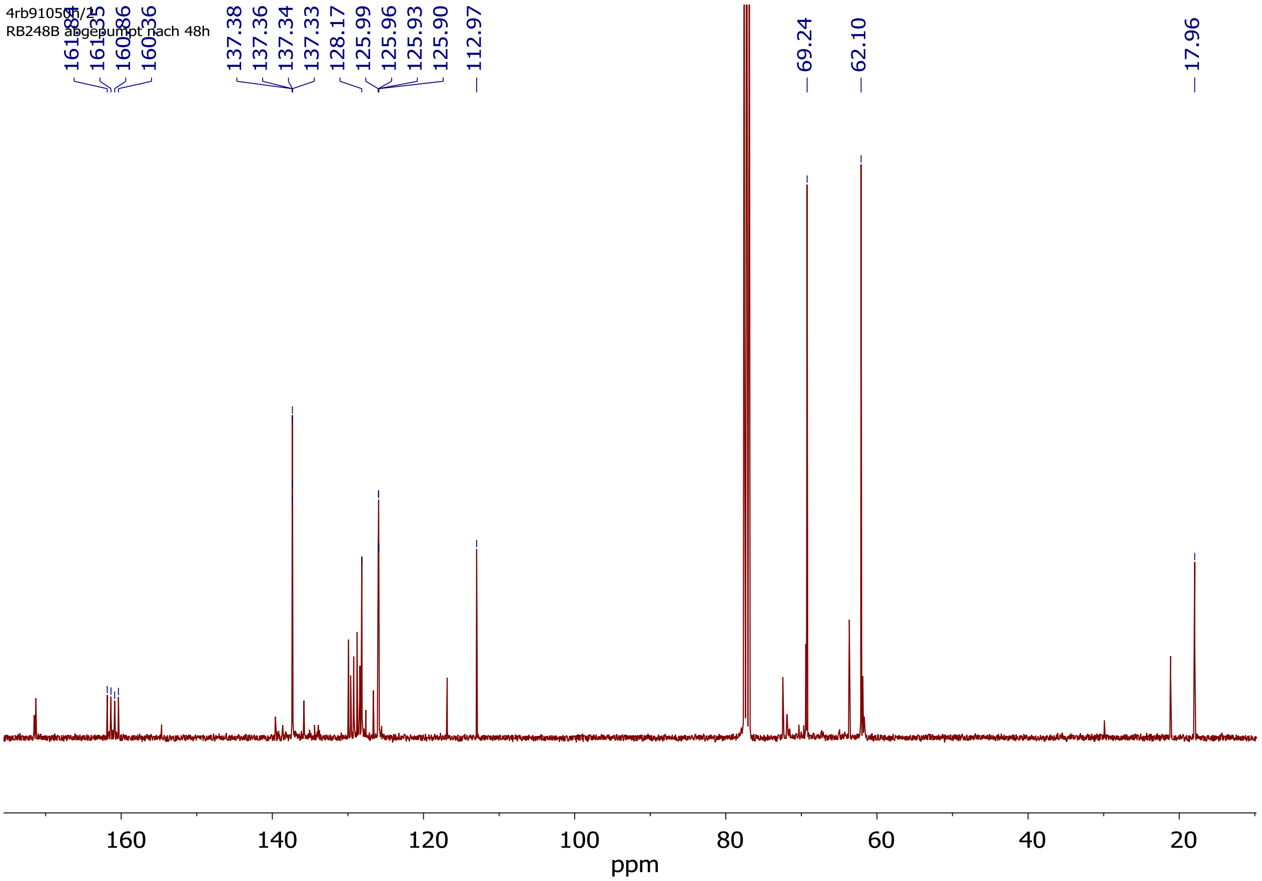

Supplementary Figure 34 | ^{13}C NMR spectrum of [Na^{+}⊂*o*-Me$_2$-1.1.1]TCPB^{-}. (100 MHz, $CDCl_3$, 298 K).

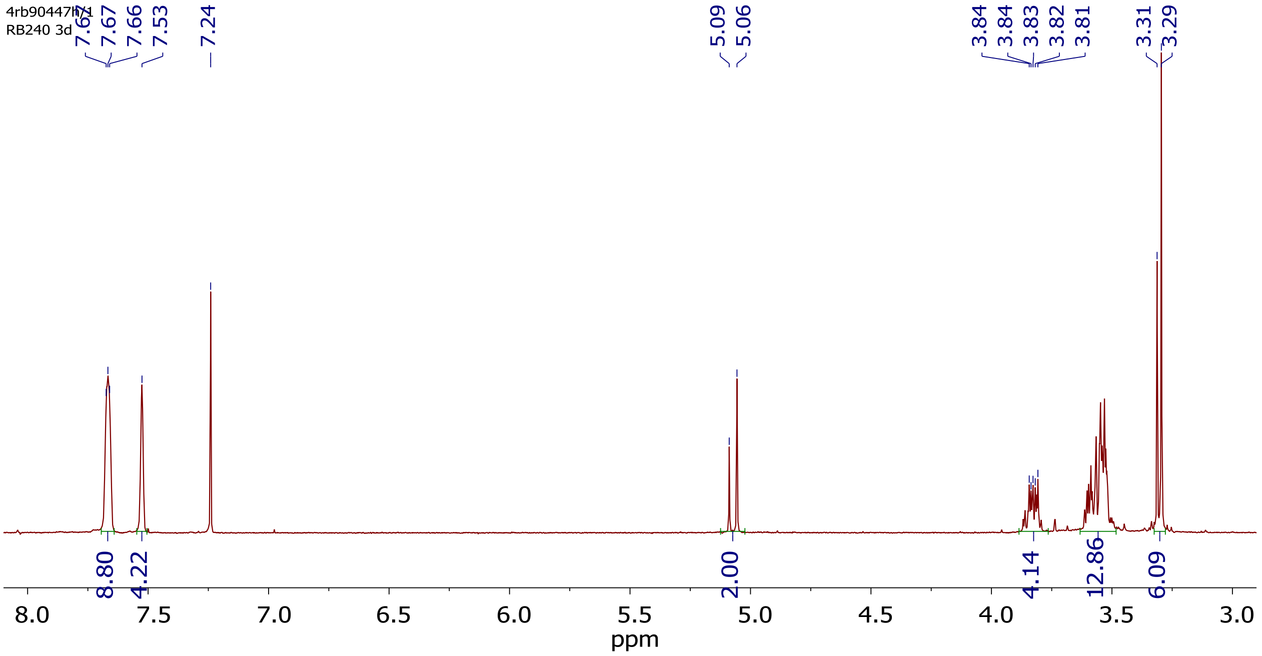

Supplementary Figure 35 | ^{1}H NMR spectrum of [Na^{+}⊂*o*-H$_2$-(OMe)$_2$-16-crown-6]BArF^{-} *syn/anti*). (400 MHz, $CDCl_3$, 298 K).

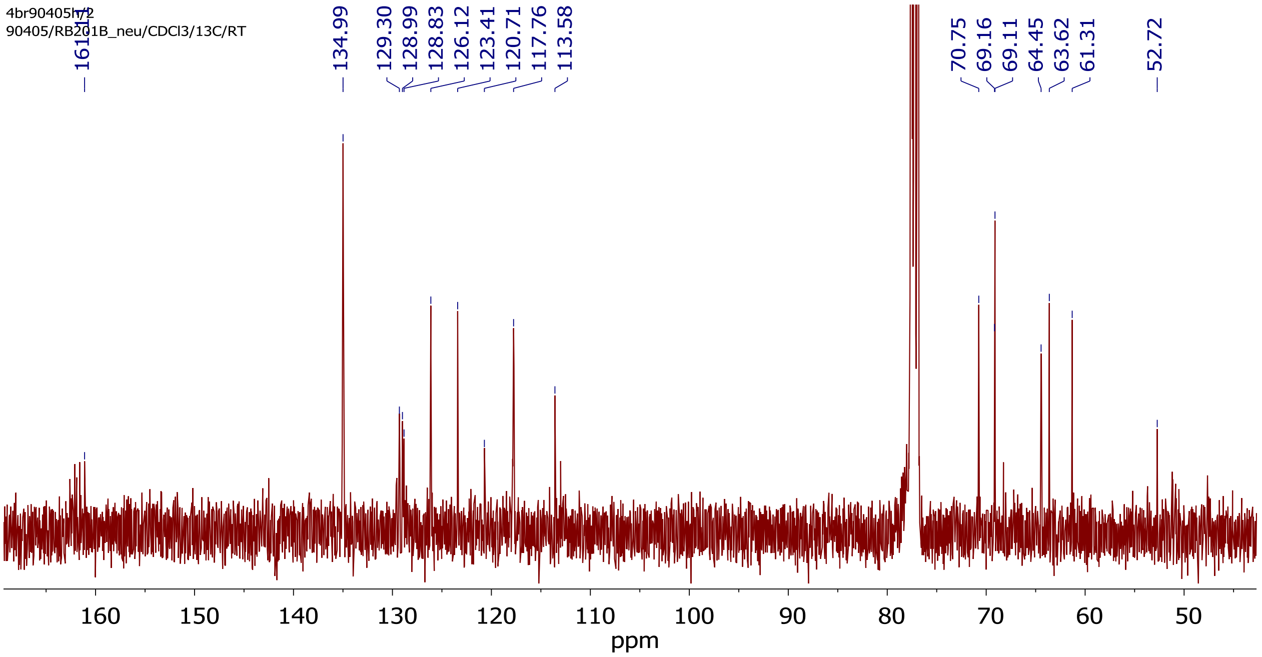

Supplementary Figure 36 | ^{13}C NMR spectrum of [Na^{+}⊂*o*-H$_2$-(OMe)$_2$-16-crown-6]BArF^{-} *syn/anti*). (100 MHz, $CDCl_3$, 298 K).

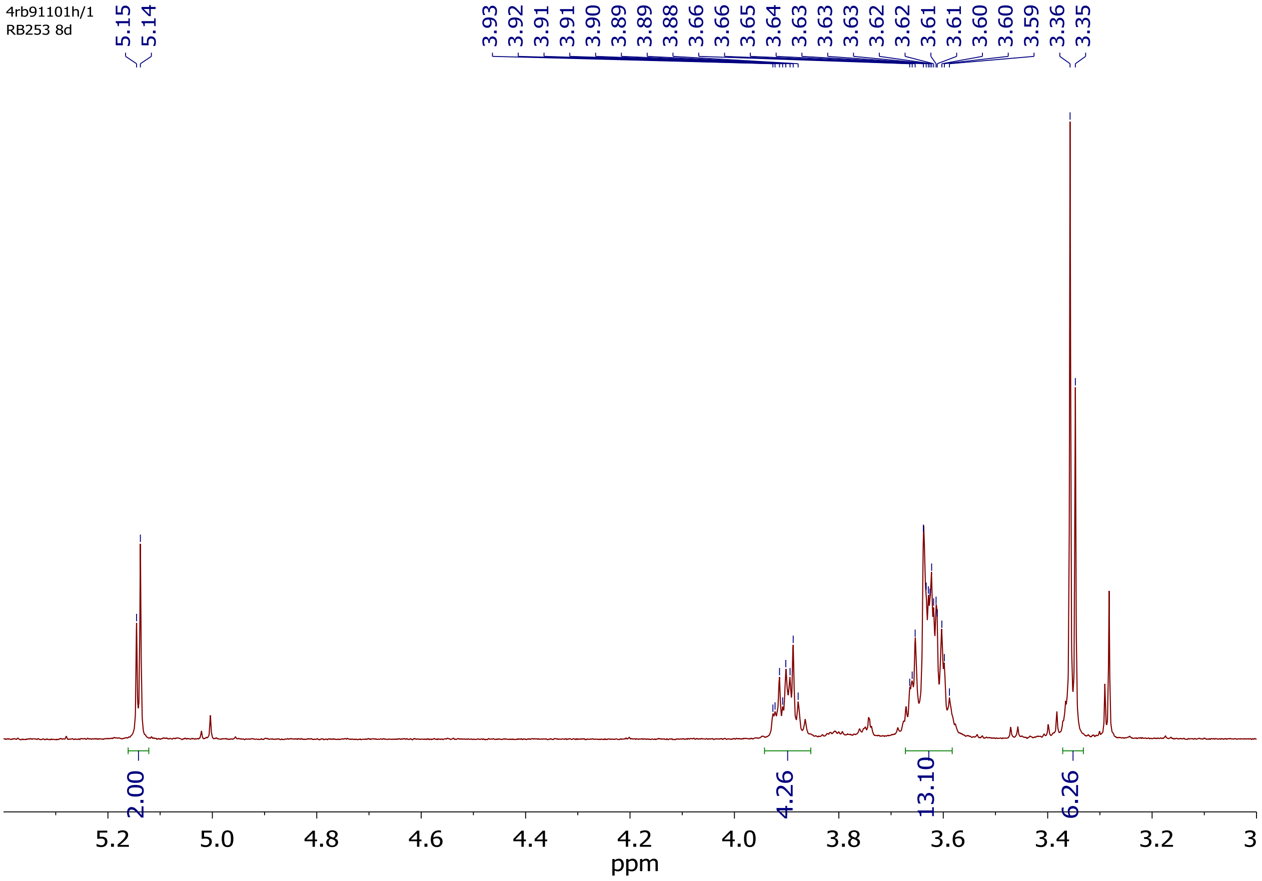

Supplementary Figure 37 | ^{1}H NMR spectrum of [M^{+}⊂*o*-H$_2$-(OMe)$_2$-16-crown-6]TPFPB^{-} (*syn/anti*). (400 MHz, $CDCl_3$, 298 K)**.**

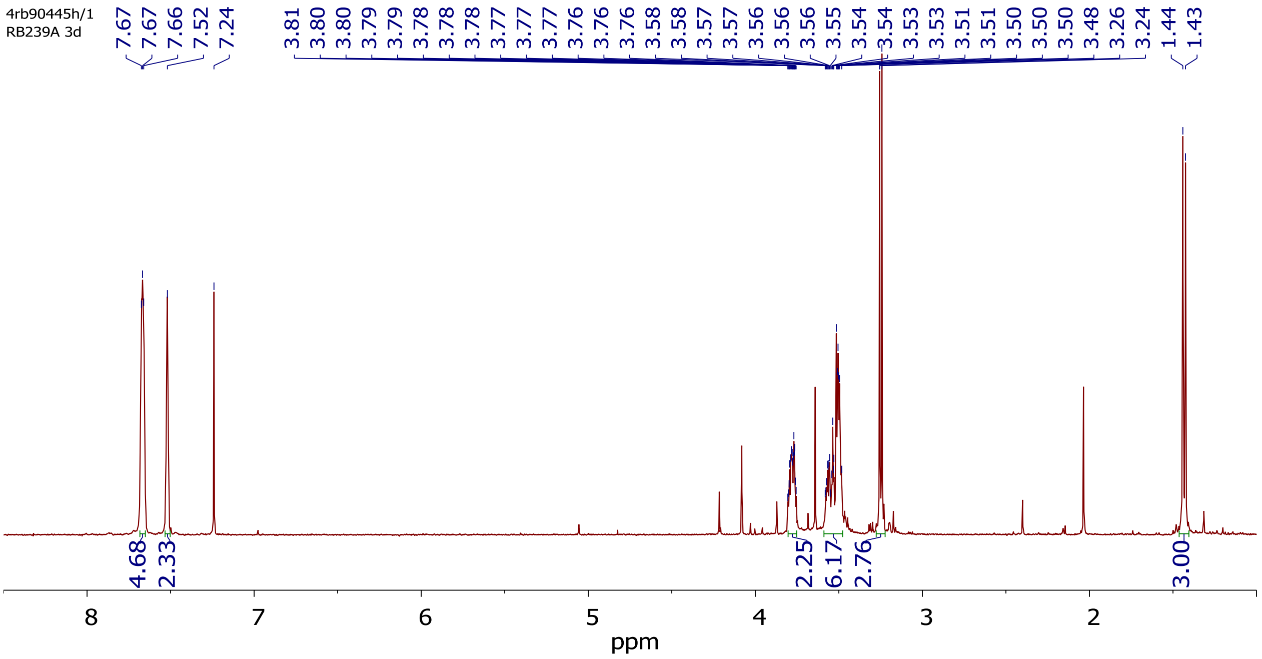

Supplementary Figure 38 | ^{1}H NMR spectrum of [Na^{+}⊂*o*-Me$_2$-(OMe)$_2$-16-crown-6]BArF^{-} (*syn/anti*). (400 MHz, $CDCl_3$, 298 K)**.**

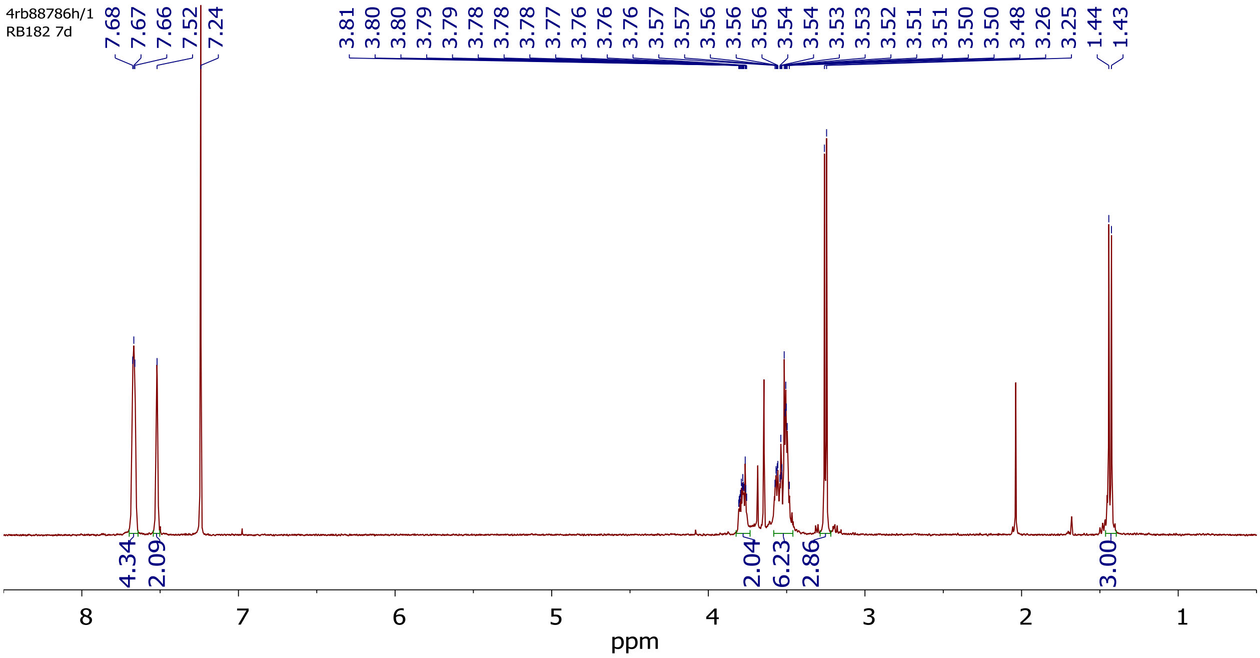

Supplementary Figure 39 | ^{1}H NMR spectrum of [M$^+$⊂*o*-Me$_2$-(OMe)$_2$-16-crown-6]BArF$^-$ (*syn/anti*). (400 MHz, $CDCl_3$, 298 K)**.**

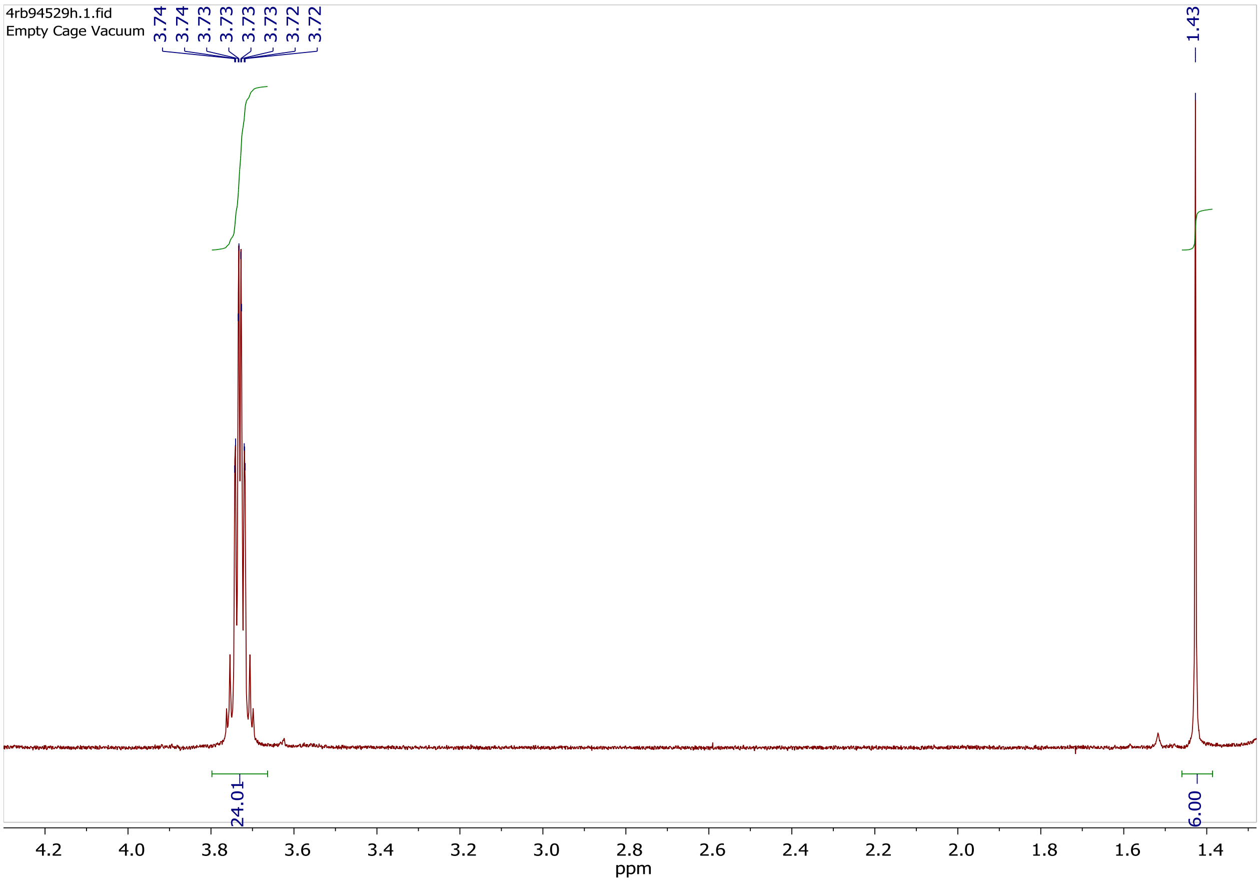

Supplementary Figure 40 | ^{1}H NMR spectrum of *o*-Me$_2$-1.1.1. (400 MHz, $CDCl_3$, 298 K)**.**

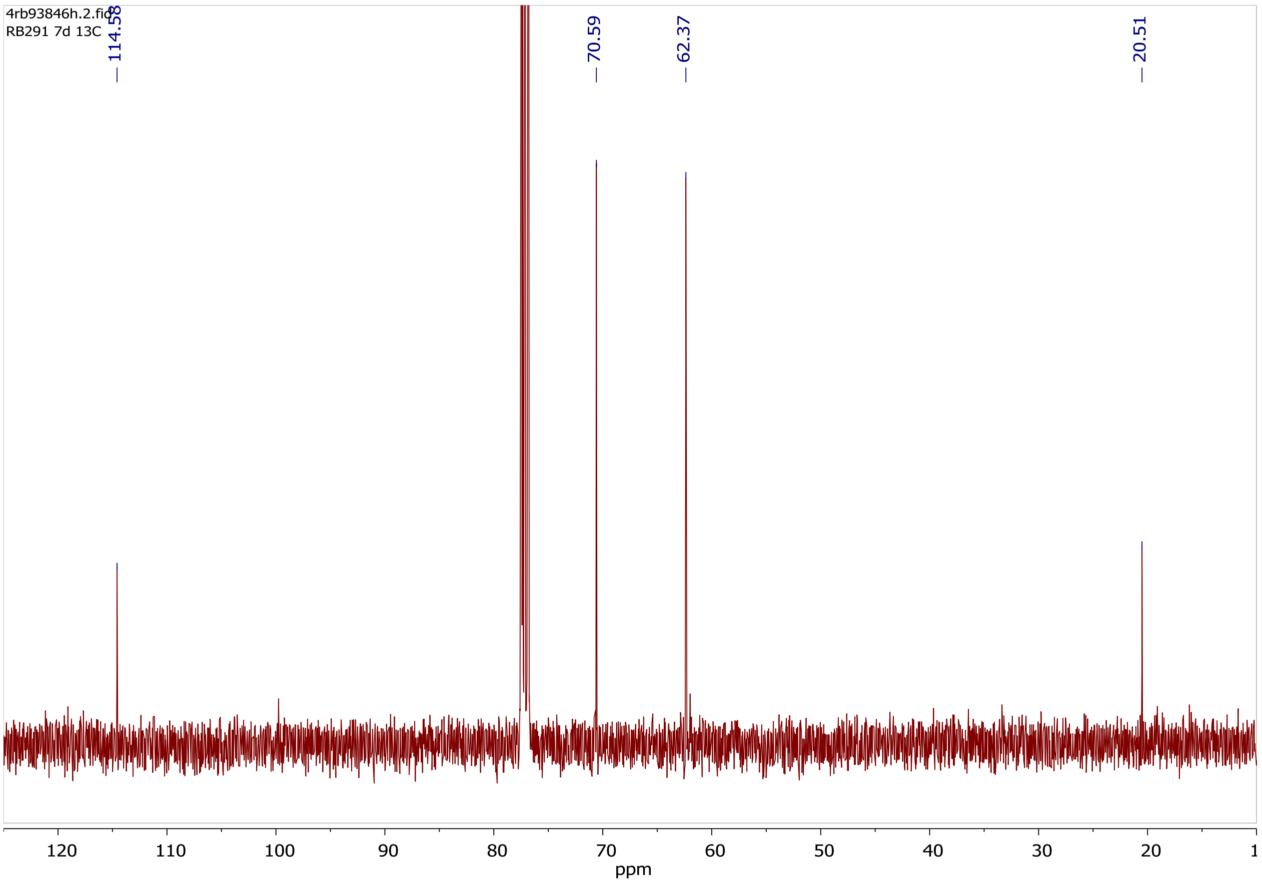

Supplementary Figure 41 | ^{13}C NMR spectrum of *o*-Me$_2$-1.1.1. (100 MHz, $CDCl_3$, 298 K)**.**

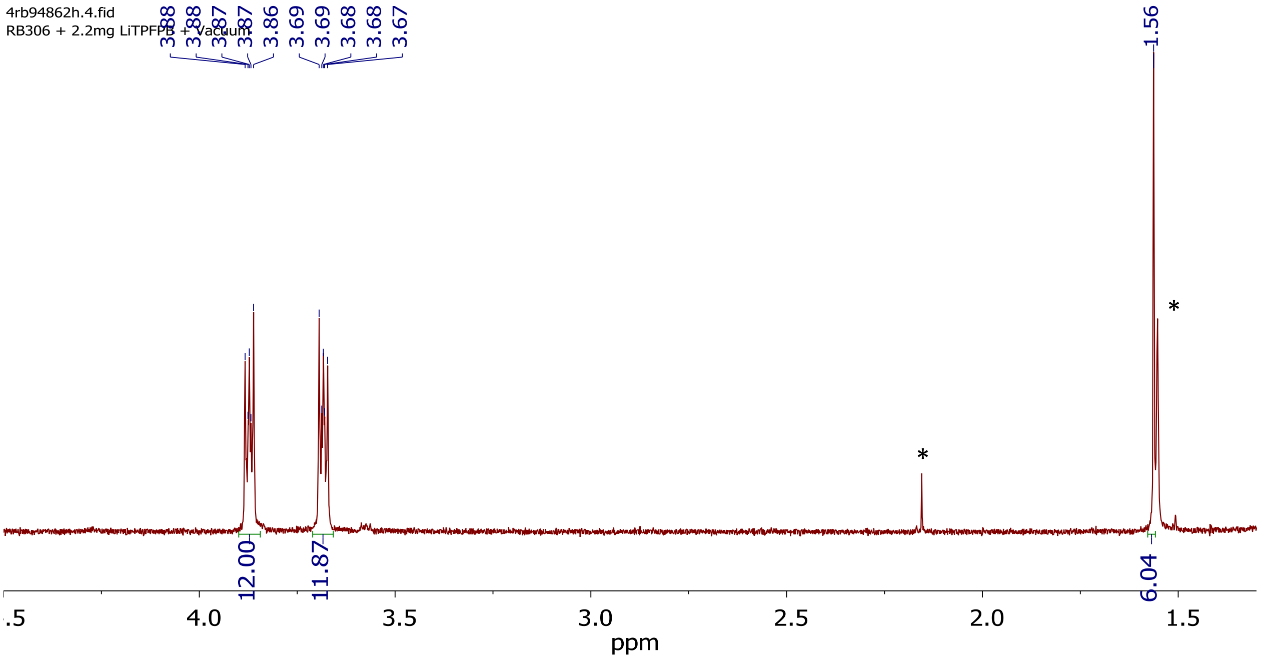

Supplementary Figure 42 | ^{1}H NMR spectrum of [Li^{+}⊂*o*-Me$_2$-1.1.1]TPFPB^{-}. (400 MHz, $CDCl_3$, 298 K)**.** *: Acetone and water

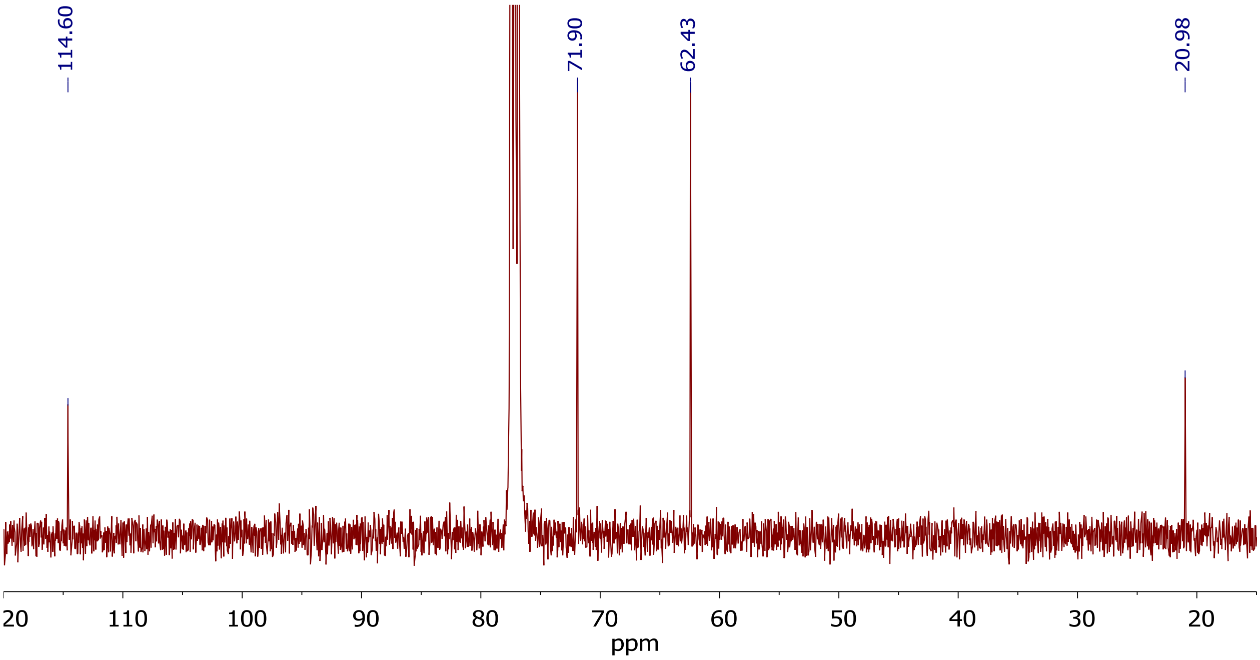

Supplementary Figure 43 | ^{13}C NMR spectrum of [Li^{+}⊂*o*-Me$_2$-1.1.1]TPFPB^{-}. (400 MHz, CDCl$_3$, 298 K)**.** Note: the ^{13}C NMR signals corresponding to the counteranion are omitted due to extensive coupling with the ^{19}F and ^{11}B nuclei.

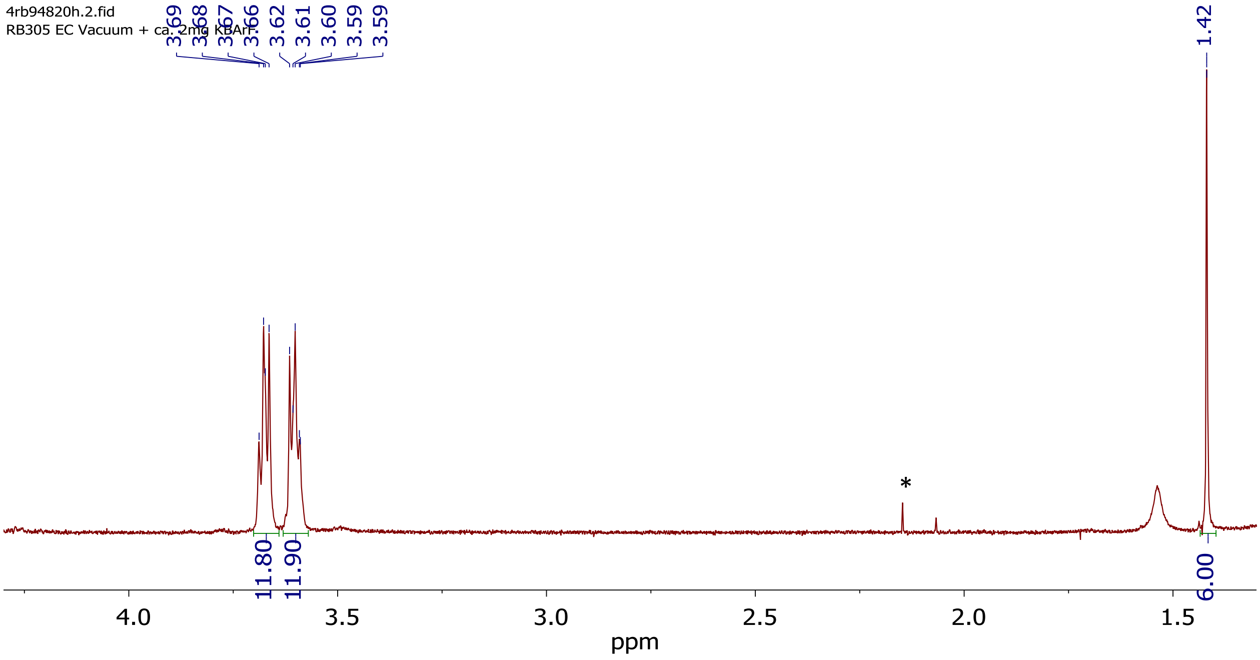

Supplementary Figure 44 | ^{1}H NMR spectrum of [K^{+}•*o*-Me$_2$-1.1.1]BArF^{-}. (400 MHz, CDCl$_3$, 298 K)**.**
*: acetone (impurity in KBArF salt)

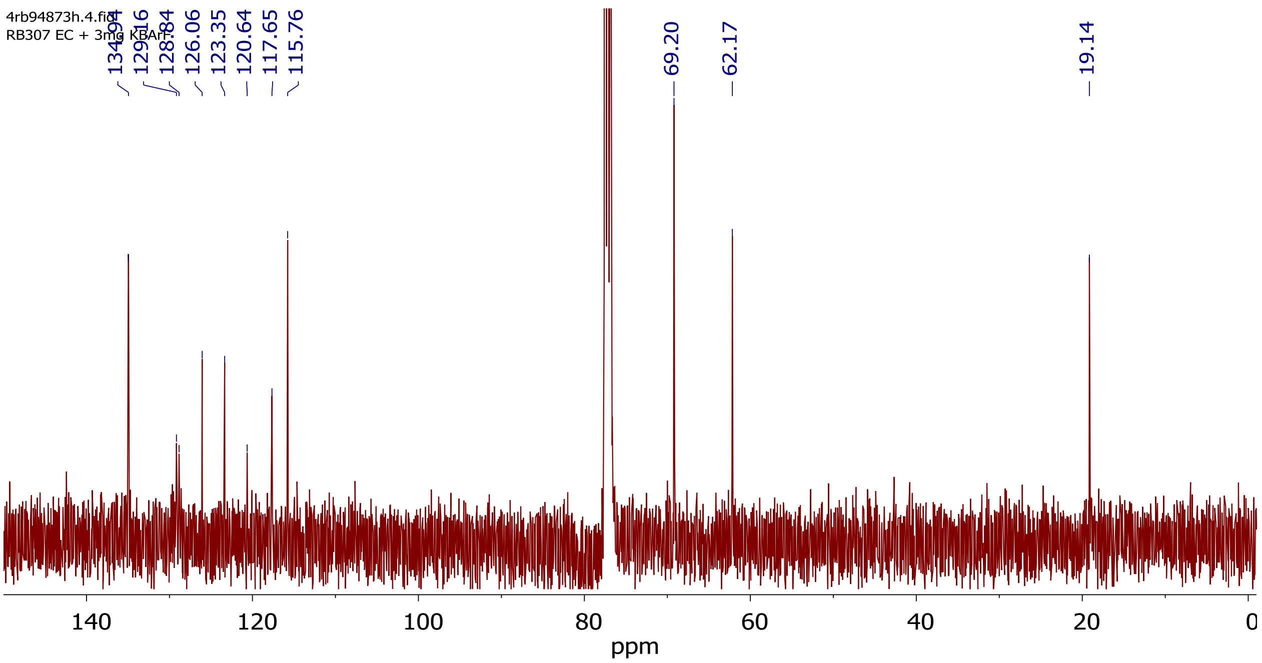

Supplementary Figure 45 | ^{13}C NMR spectrum of [K^{+}•*o*-Me$_2$-1.1.1]BArF^{-}. (100 MHz, $CDCl_3$, 298 K)**.**

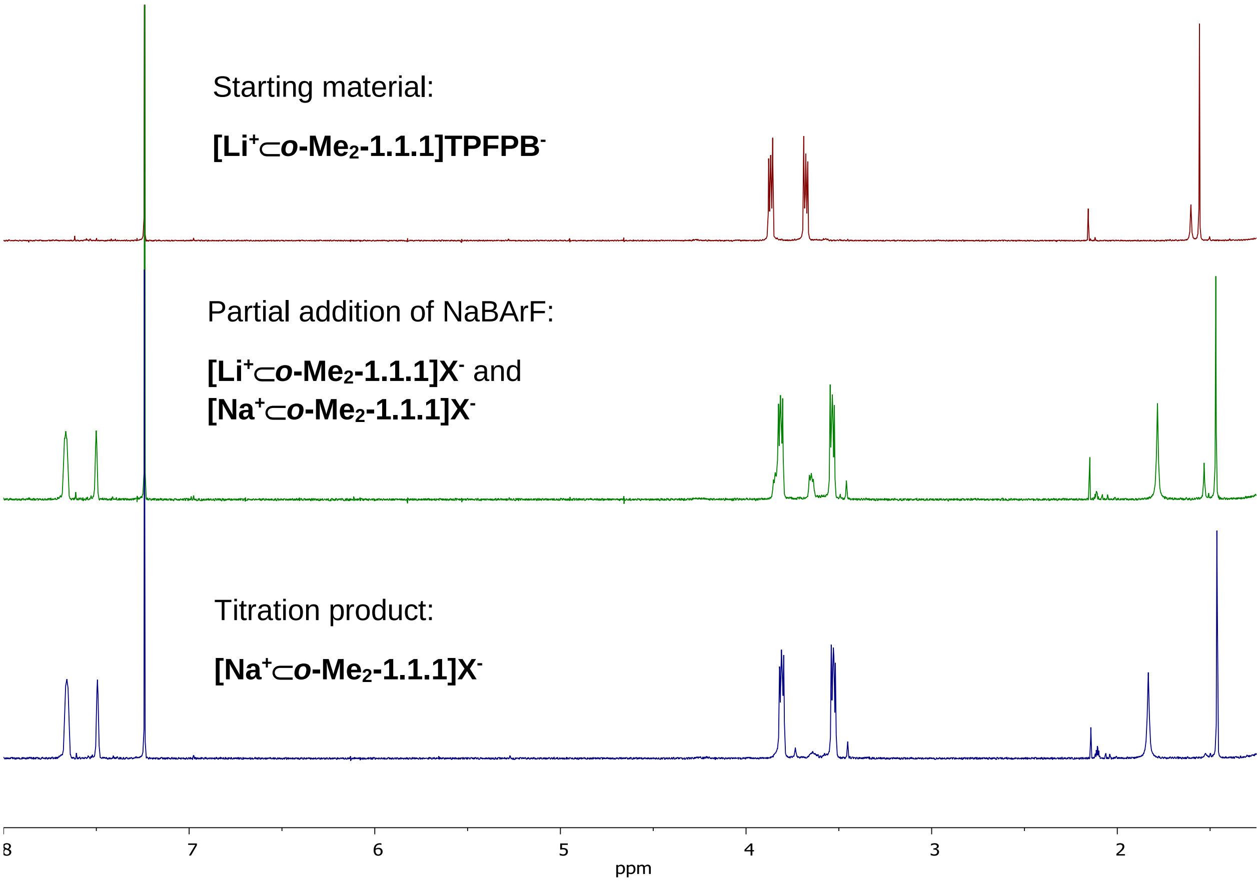

Supplementary Figure 46 | Synthesis of [Na$^+$⊂*o*-Me$_2$-1.1.1]X$^-$ from [Li$^+$⊂*o*-Me$_2$-1.1.1]TPFPB$^-$. (^{1}H NMR, 400 MHz, CDCl$_3$, 298 K)**.** A highly concentrated stock solution of sodium tetrakis[3,5-bis(trifluoromethyl)phenyl]borate (NaBArF) was prepared in acetone-d$_6$. This stock solution was titrated to a solution of **[Li$^+$⊂*o*-Me$_2$-1.1.1]TPFPB$^-$** in CDCl$_3$. For preparation of Cryptate **[Li$^+$⊂*o*-Me$_2$-1.1.1]TPFPB$^-$** see Supplementary Methods. X$^-$ indicates mixture of counteranions

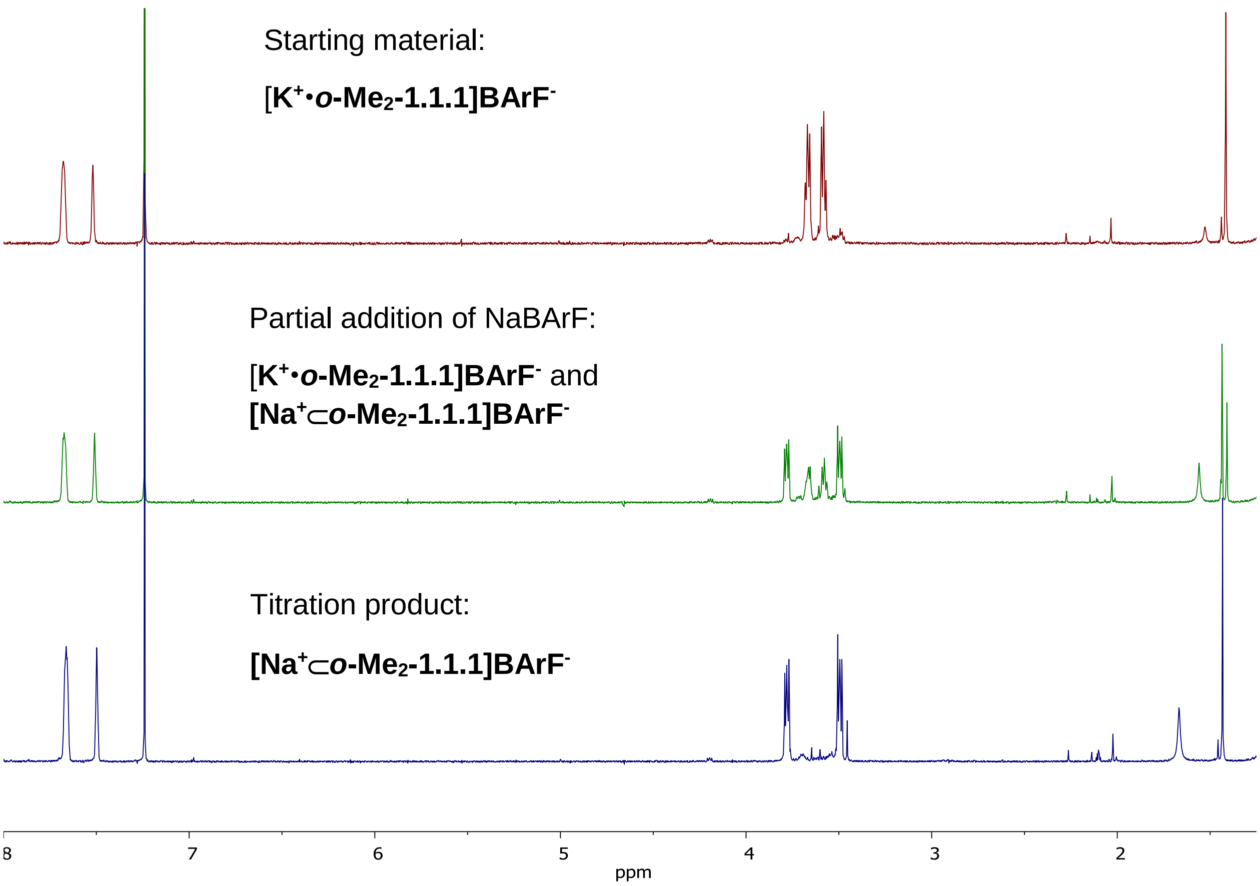

Supplementary Figure 47 | Synthesis of [Na^+⊂*o*-Me_2-1.1.1]$BArF^-$ from [K^+•*o*-Me_2-1.1.1]$BArF^-$. (^{1}H NMR, 400 MHz, $CDCl_3$, 298 K)**.** A highly concentrated stock solution of sodium tetrakis[3,5-bis(trifluoromethyl)phenyl]borate (NaBArF) was prepared in acetone-d_6. This stock solution was titrated to a solution of [**K^+•*o*-Me_2-1.1.1]$BArF^-$** in $CDCl_3$. For preparation of Cryptate [**K^+•*o*-Me_2-1.1.1]$BArF^-$** see Supplementary Methods.

Supplementary Tables

Goniometer type:	Agilent SuperNova with Atlas detector
Structure solution :	SHELXS-2013
Structure refinement :	SHELXL-2013
Hydrogen treatment:	riding model
**Disorder**:	**Cage: 50:50 % disorder of the three aliphatic chains. For the structure presented in the manuscript we removed duplicate atoms (assuming reasonable bond lengths, dihedral angles and symmetry) for reasons of clarity.**<br>CF_3-Groups also show some slight disorder
Comments:	Asym. unit contains some disordered solvent molecule (DCM?), which could not be resolved, and therefore was masked during refinement

Supplementary Table 1 | Notes on structure solution and refinement for [Na^+⊂*o*-Me_2-1.1.1]$BArF^-$

Identification code	rb01_4
Empirical formula	$C_{48}H_{42}BF_{24}NaO_9Cl_{0.25}$
Formula weight	1261.48
Temperature/K	107.00(4)
Crystal system	monoclinic
Space group	C2/m
a/Å	18.3769(4)
b/Å	16.7983(4)
c/Å	18.7409(5)
α/°	90
β/°	107.372(3)
γ/°	90
Volume/Å^3	5521.4(2)
Z	4
ρ_{calc}mg/mm^3	1.518
μ/mm^{-1}	1.577
F(000)	2553.0
Crystal size/mm^3	0.2637 × 0.1303 × 0.0996
2Θ range for data collection	7.28 to 122.82°
Index ranges	$-17 \leq h \leq 20$, $-18 \leq k \leq 11$, $-16 \leq l \leq 21$
Reflections collected	6750
Independent reflections	4259[R(int) = 0.0346]
Data/restraints/parameters	4259/16/432
Goodness-of-fit on F^2	1.075
Final R indexes [I>=2σ (I)]	R_1 = 0.0667, wR_2 = 0.1749
Final R indexes [all data]	R_1 = 0.0881, wR_2 = 0.1936
Largest diff. peak/hole / e Å^{-3}	0.48/-0.41

Supplementary Table 2 | Crystal data for [$Na^+ \subset$*o*-Me_2-1.1.1]$BArF^-$.

Supplementary Notes

Supplementary Note 1

Calculation of k_{obs} based on EXSY data:

Using the equations shown below, where I_{AA}, I_{BB} (½) and I_{CC} are the diagonal peak integrals and I_{AB}, I_{BA}, I_{BC} and I_{CB} are the cross-peak integrals, we obtain the magnetization rate r, from which we calculate k which is the *sum* of the forward, k_1, and backward, k_{-1}, pseudo-first order rate constants for the cation exchange process. As both hosts are identical k_1 and k_{-1} are equal they correspond to the observed pseudo-first order rate constant k_{obs} (see previous page for equilibrium under study). This calculation can be performed for both CH_2 groups of the cryptate (A and C) and the reported value for k_{obs} (0.6 s^{-1}; estimated error ±0.2 s^{-1}) corresponds to the average value (k_{obsA} = 0.68 s^{-1}; k_{obsC} = 0.61 s^{-1}).

Equation 1 $$r = \frac{I_{AA}+I_{BB}}{I_{AB}+I_{BA}}$$

Equation 2 $$k = \frac{1}{\tau_m} \times ln\frac{r+1}{r-1}$$

Equation 3 $$k = k_1 + k_{-1}$$

Equation 4 $$k_{obs} = k_1 = k_{-1}$$

See also Supplementary Figure 23.

Supplementary Methods

General Experimental Section

Reagents and instruments

All commercially purchased reagents were used without further purification. Molecular sieves were dried for 3 days at 150 °C under reduced pressure (10^{-2} mbar) before use. $CDCl_3$, DMSO-d_6, MeCN-d_3 and benzene-d_6 were stored over molecular sieves. NMR spectra were recorded on Bruker Avance 300 (^{1}H: 300 MHz), Bruker Avance 400 (^{1}H: 400 MHz), Jeol EX 400 (^{1}H: 400 MHz) and Jeol Alpha 500 (^{1}H: 500 MHz) spectrometers at 298 K and referenced to the residual solvent peak (^{1}H: $CDCl_3$, 7.24 ppm; ^{13}C: $CDCl_3$, 77.16 ppm). Coupling constants (*J*) are denoted in Hz and chemical shifts (δ) in ppm. Mass spectra were obtained on Bruker micro TOF II and Bruker Maxis 4G (ESI^+, Toluene/Acetonitrile) instruments. HPLC-MS analysis was performed on a Shimadzu LCMS 2020 instrument (column: Kinetex C18, 2.6 µm, 100 x 4.6 mm; mobile phase: H_2O/MeOH 80% → 100 % MeOH; flow rate: 0.3 mL/min). IR spectra were recorded on a Bruker Tensor 27- IR spectrometer as solids in ATR-mode. Atomic absorption and atomic emission spectra were recorded on a Shimadzu AA-7000F instrument.

Preparation of stock solutions

To achieve a high level of stoichiometric accuracy and adequate exclusion of moisture, metal salts and diethylene glycol were added from stock solutions that were dried over molecular sieves prior to addition of orthoester and acid catalyst.

In a typical example for the **salt stock solution**, sodium tetrakis[3,5-bis(trifluoromethyl)phenyl]borate (NaBArF, 0.14 mmol, 127.8 mg) and diethylene glycol (DEG, 0.42 mmol, 39.9 µL) were dissolved in $CDCl_3$ (14 mL) and dried over 4 Å molecular sieves (ca. 1 g) for 3 days.

To obtain the **acid stock solution**, trifluoroacetic acid (TFA, 240 µmol, 18.4 µL) was topped up with $CDCl_3$ to obtain a total volume of 2 mL.

General procedure

Molecular sieves 4 Å (1 g) were added to 6.0 mL of the **salt stock solution** and the reaction mixture was left to stand at room temperature for 16 h. The orthoester (0.12 mmol) and 10 µL of the **acid stock solution** (1 mol%) was added and the reaction mixture was shaken. Every 24 h, 10 µL of the **acid stock solution** (1 mol%) was added to keep the exchange reaction active (molecular sieves slowly transform the acid catalyst into inactive anhydride and/or esters) and the reaction progress was monitored regularly by ^{1}H NMR spectroscopy.

Exclusion of moisture

Molecular sieves were dried by heating for 3 days at 150 °C under reduced pressure (10^{-2} mbar). All solvents were dried over molecular sieves for at least 24 hours. All orthoester exchange reactions (catalyzed by TFA) were carried out under nitrogen. After the acid is quenched (e.g., by addition of triethylamine or basic aluminum oxide), many of the orthoesters described herein were found to be unusually stable against water (see Supplementary Figure 2).

Synthesis of [Na$^+$⊂*o*-Me$_2$-1.1.1]BArF$^-$

[Na$^+$⊂*o*-Me$_2$-1.1.1]BArF$^-$ was prepared according to the general procedure from NaBArF, trimethyl orthoacetate and diethylene glycol. After 5 days, the solvent was removed under reduced pressure and the title compound was obtained as a colourless solid in 67% yield. Further purification could be achieved by passing a solution of the crude mixture in chloroform through a short plug of silica gel or by crystallization (e.g. slow diffusion of cyclopentane).

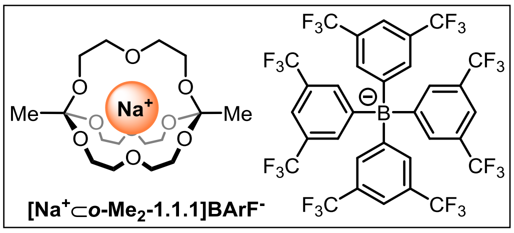

M. p.: 124-128 °C.

^{1}H NMR (400 MHz, CDCl$_3$): δ = 7.68 (t, *J* = 2.8 Hz, 8H), 7.51 (s, 4H), 3.79 – 3.77 (m, 12H), 3.50 – 3.48 (m, 12H), 1.43 ppm (s, 6H).

^{13}C NMR (100 MHz, CDCl$_3$): δ = 162.8, 162.3, 161.8, 161.3, 135.1, 129.7, 129.4, 129.1, 128.9, 128.8, 126.2, 123.5, 120.8, 117.7, 113.0, 69.2, 62.0, 17.7 ppm.

^{11}B NMR (128 MHz, CDCl$_3$): δ = -6.7 ppm.

^{19}F NMR (282 MHz, CDCl$_3$): δ = -62.1 ppm.

^{23}Na NMR (132 MHz, CDCl$_3$): δ = -5.9 ppm.

HRMS (ESI$^+$): *m*/*z* = 389.1794 [M+Na]$^+$ (calcd. 389.1782 for $C_{16}H_{30}O_9Na$).

IR (ATR, RT): ṽ = 668, 681, 714, 745, 839, 884, 896, 932, 970, 1064, 1112, 1160, 1271, 1353, 1610, 2911 cm^{-1}.

Synthesis of [Na$^+$⊂*o*-Et$_2$-1.1.1]BArF$^-$

[Na$^+$⊂*o*-Et$_2$-1.1.1]BArF$^-$ was prepared according to the general procedure from NaBArF, trimethyl orthopropionate and diethylene glycol. After 5 days, the solvent was removed under reduced pressure and the title compound was obtained as a colourless solid in 52% yield. Further purification could be achieved by passing a solution of the crude mixture through a short plug of aluminium oxide or silica gel.

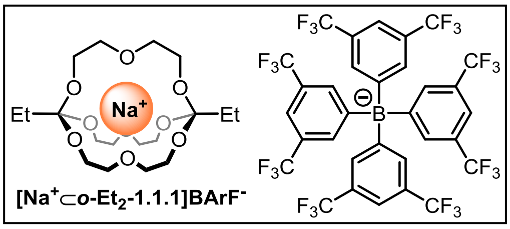

^{1}H NMR (400 MHz, CDCl$_3$): δ = 7.68 (t, *J* = 2.8 Hz, 8H), 7.51 (s, 4H), 3.99 – 3.68 (m, 12H), 3.64 – 3.35 (m, 12H), 1.85 (q, *J* = 7.5 Hz, 4H), 0.94 (t, *J* = 7.5 Hz, 6H).

^{13}C NMR (100 MHz, CDCl$_3$): δ = 162.8, 162.3, 161.8, 161.3, 135.1, 129.7, 129.4, 129.1, 128.9, 128.8, 126.2, 123.5, 120.8, 117.7, 114.3, 69.7, 62.6, 27.2, 7.7.

HRMS (ESI$^+$): *m*/*z* = 417.2091 [M+Na]$^+$ (calcd. 417.2095 for $C_{18}H_{34}O_9Na$).

Synthesis of [Na$^+$⊂*o*-Me$_2$-1.1.1]TCPB$^-$

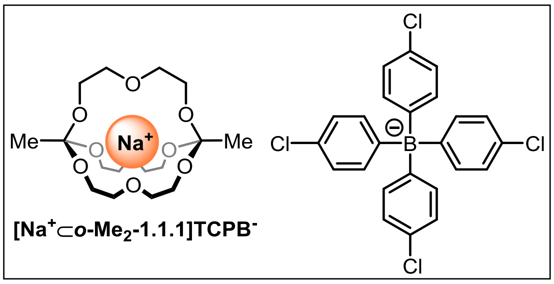

Potassium[i] tetrakis(4-chlorophenyl)borate (**K**TCPB) and 4 Å molecular sieves (1 g) were added to a solution of diethylene glycol (0.18 mmol, 17.1 µL) in $CDCl_3$ and the reaction mixture was left to stand at room temperature. After 16 h, 1 mol% TFA (10 µL) was added from a stock solution, the mixture was shaken and trimethyl orthoacetate (0.12 mmol, 15.4 µL) was added. Every 24 hours, 1 mol% TFA was added to keep the exchange reaction active. The reaction progress was monitored regularly by ^{1}H NMR spectroscopy. After 3 days, the solvent was removed under reduced pressure and the title compound was obtained in 69% yield (yield was determined by ^{1}H NMR with 1,4-dinitrobenzene as internal standard).

^{1}H NMR (400 MHz, $CDCl_3$): δ = 7.54 (d, *J* = 8.4 Hz, 4H), 7.46 (d, *J* = 8.4 Hz , 4H), 7.00 (d, *J* = 8.2 Hz , 8H), 3.78 – 3.76 (m, 12H), 3.49 – 3.47 (m, 12H), 1.44 (s, 6H).

^{13}C NMR (100 MHz, $CDCl_3$): δ = 161.8, 161.3, 160.9, 160.4, 137.3, 128.1, 125.9, 112.9, 69.2, 62.0, 18.0.

HRMS (ESI$^+$): *m/z* = 389.1809 [M+Na]$^+$ (calcd. 389.1782 for $C_{16}H_{30}O_9Na$).

Synthesis of [Na$^+$⊂*o*-H$_2$-(OMe)$_2$-16-crown-6]BArF$^-$ (*syn/anti*)

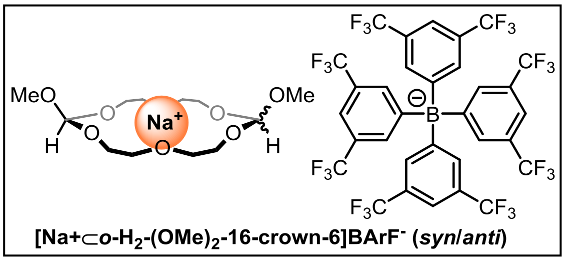

[Na$^+$⊂*o*-H$_2$-(OMe)$_2$-16-crown-6]BArF$^-$ was prepared according to the general procedure from NaBArF (1 equiv.), trimethyl orthoformate (2 equiv.) and diethylene glycol (2 equiv.). The reaction progress was monitored regularly by ^{1}H NMR spectroscopy. After 2 days, the solvent was removed under reduced pressure and the title compound was obtained as a mixture of *syn* and *anti* isomers in 69% yield (yield was determined by ^{1}H NMR with 1,4-dinitrobenzene as internal standard).

^{1}H NMR (400 MHz, $CDCl_3$): δ = 7.67 (t, *J* = 2.8 Hz, 8H), 7.52 (s, 4H), 5.09 (s, 2H), 5.06 (s, 2H), 3.87 – 3.81 (m, 4H), 3.62 – 3.52 (m, 12H), 3.31 (s, 6H), 3.30 (s, 6H).

^{13}C NMR (100 MHz, $CDCl_3$): δ = 162.6, 162.1, 161.6, 161.1, 135.0, 129.6, 129.3, 129.0, 128.8, 128.7, 126.1, 123.4, 120.7, 117.8, 113.6, 113.0, 77.4, 70.7, 69.2, 69.1, 64.5, 64,4, 63.6, 61.3, 52.7.

HRMS (ESI): *m/z* = 319.1366 [M+Na]$^+$ (calcd. 319.1363 for $C_{12}H_{24}O_8Na$).

Synthesis of [M$^+$⊂*o*-H$_2$-(OMe)$_2$-16-crown-6]TPFPB$^-$ (*syn*/*anti*)

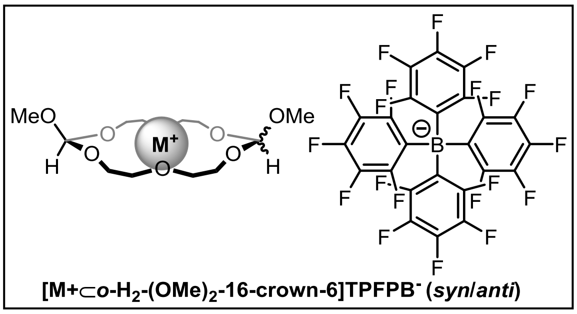

[M$^+$⊂*o*-H$_2$-(OMe)$_2$-16-crown-6]TPFPB$^-$ was prepared according to the general procedure from **K**TPFPB[i] (1 equiv.), trimethyl orthoformate (2 equiv.) and diethylene glycol (2 equiv.). The reaction progress was monitored regularly by ^{1}H NMR spectroscopy. After 8 days, the solvent was removed under reduced pressure and the title compound was obtained as a mixture of *syn* and *anti* isomers in 46% yield (yield was determined by ^{1}H NMR with 1,4-dinitrobenzene as internal standard). M$^+$: mixture of some K$^+$ and mostly Na$^+$ generated via cation exchange with MS 4Å (see section 11).

^{1}H NMR (400 MHz, CDCl$_3$): δ = 5.15 (s, 2H), 5.14 (s, 2H), 3.93 – 3.88 (m, 4H), 3.66 – 3.59 (m, 12H), 3.36 (s, 6H), 3.35 (s, 6H).

HRMS (ESI): *m/z* = 335.1097 [M+K]$^+$ (calcd. 335.1103 for $C_{12}H_{24}O_8K$).

Synthesis of [Na$^+$⊂*o*-Me$_2$-(OMe)$_2$-16-crown-6]BArF$^-$ (*syn*/*anti*)

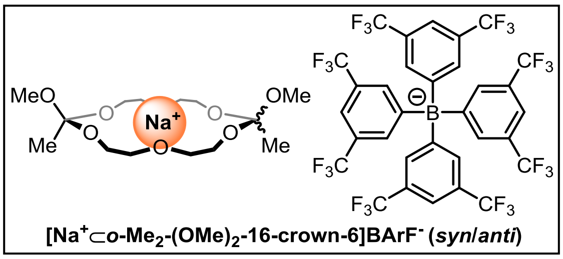

[Na$^+$⊂*o*-Me$_2$-(OMe)$_2$-16-crown-6]BArF$^-$ was prepared according to the general procedure from **NaBArF** (1 equiv.), trimethyl orthoacetate (2 equiv.) and diethylene glycol (2 equiv.). The reaction progress was monitored regularly by ^{1}H NMR spectroscopy. After 3 days, the title compound was obtained as a mixture of *syn* and *anti* isomers in (65% yield) (yield was determined by ^{1}H NMR with 1,4-dinitrobenzene as internal standard).

^{1}H NMR (400 MHz, CDCl$_3$): δ = 7.67 (t, *J* = 2.8 Hz, 8H), 7.52 (s, 4H), 3.81 – 3.76 (m, 4H), 3.58 – 3.48 (m, 12H), 3.26 (s, 3H), 3.24 (s, 3H), 1.44 (s, 3H), 1.43 (s, 3H).

HRMS (ESI): *m/z* = 347.1670 [M+Na]$^+$ (calcd. 347.1676 for $C_{14}H_{28}O_8Na$).

Synthesis of [M^+⊂*o*-Me$_2$-(OMe)$_2$-16-crown-6]BArF$^-$ (*syn*/*anti*)

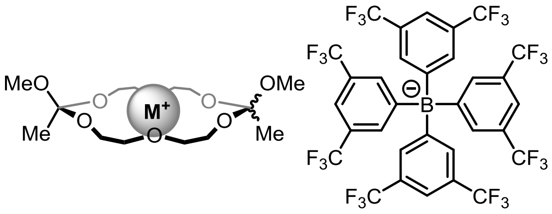

[M+⊂*o*-Me$_2$-(OMe)$_2$-16-crown-6]BArF$^-$ (*syn*/*anti*)

[M^+⊂*o*-Me$_2$-(OMe)$_2$-16-crown-6]BArF$^-$ was prepared according to the general procedure from **K**BArF[i] (1 equiv.), trimethyl orthoacetate (2 equiv.) and diethylene glycol (2 equiv.). The reaction progress was monitored regularly by ^{1}H NMR spectroscopy. After 7 days, the solvent was removed under reduced pressure and the title compound was obtained as a mixture of *syn* and *anti* isomers in 57% yield (yield was determined by ^{1}H NMR with 1,4-dinitrobenzene as internal standard). M^+: mixture of some K^+ and mostly Na^+ generated via cation exchange with MS 4Å (see section 11).

^{1}H NMR (400 MHz, $CDCl_3$): δ = 7.67 (t, J = 2.8 Hz, 8H), 7.52 (s, 4H), 3.81 – 3.76 (m, 4H), 3.57 – 3.48 (m, 12H), 3.26 (s, 3H), 3.25 (s, 3H), 1.44 (s, 3H), 1.43 (s, 3H).

Synthesis of *o*-Me$_2$-1.1.1

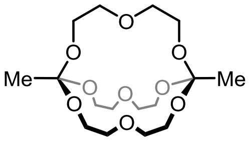

o-Me$_2$-1.1.1

[Na^+⊂*o*-Me$_2$-1.1.1]BArF$^-$ (~2 mg) was stirred in $CDCl_3$ with chloride exchange resin (Lewatite MP-64, 300 mg) for 6 h at RT. The reaction progress was monitored by ^{1}H NMR spectroscopy and the anion exchange resin was removed by filtration through a syringe filter once the reaction was complete. After removal of the solvent under reduced pressure, the title compound was obtained quantitatively as a colourless oil.

^{1}H NMR (400 MHz, $CDCl_3$): δ = 3.73 (m, 24H), 1.43 (s, 6H).

^{13}C NMR (100 MHz, $CDCl_3$): δ = 114.6, 70.6, 62.4, 20.5 ppm.

HRMS (ESI$^+$): m/z = 389.1780 $[M+Na]^+$ (calcd. 389.1782 for $C_{16}H_{30}O_9Na$).

Note: Due to its high affinity for Na^+, we were not able to observe compound ***o*-Me$_2$-1.1.1** as $[M+H]^+$, $[M+Li]^+$ or $[M+K]^+$ ion, even after an excess of the corresponding salts was added to the sample.

Synthesis of [Li^+⊂*o*-Me_2-1.1.1]$TPFPB^-$

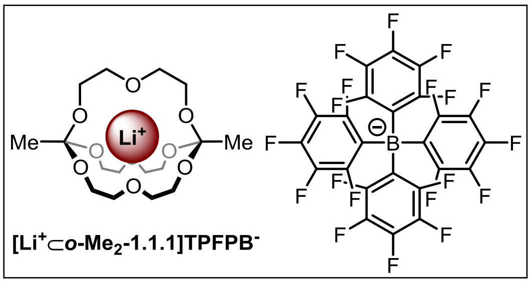

[Li^+⊂*o*-Me_2-1.1.1]$TPFPB^-$

Cryptand ***o*-Me_2-1.1.1** was prepared as described above. A stock solution of lithium tetrakis(pentafluorophenyl) borate ethyl etherate (LiTPFPB) was prepared and titrated to a solution of ***o*-Me_2-1.1.1** in $CDCl_3$.

^1H NMR (400 MHz, $CDCl_3$): δ = 3.88-3.86 (m, 12H), 3.69-3.67 (m, 12H), 1.56 (s, 6H).

^{13}C NMR (100 MHz, $CDCl_3$): δ = 114.6, 71.9, 62.4, 21.0 ppm.

^7Li NMR (194 MHz, $CDCl_3$): δ = -1.86 ppm.

HRMS (ESI^+): *m/z* = 389.1785 $[M+Na]^+$ (calcd. 389.1782 for $C_{16}H_{30}O_9Na$). Note: Under ESI conditions Li^+ is replaced with ubiquitous Na^+ (suggesting that the cage binds Na^+ preferably).

Synthesis of [K^+•*o*-Me_2-1.1.1]$BArF^-$

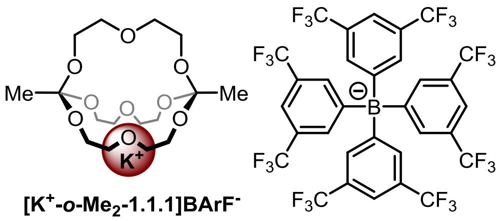

[K^+-*o*-Me_2-1.1.1]$BArF^-$

Cryptand ***o*-Me_2-1.1.1** was prepared as described above. A stock solution of potassium tetrakis[3,5-bis(trifluoromethyl)phenyl]borate (KBArF) was prepared and dried for one night over 3 Å molecular sieves (150 mg). This stock solution was titrated to a solution of ***o*-Me_2-1.1.1** in $CDCl_3$.

^1H NMR (400 MHz, $CDCl_3$): δ = 3.69 -3.66 (m, 12H), 3.62 -3.59 (m, 12H), 1.42 (s, 6H).

^{13}C NMR (100 MHz, $CDCl_3$): δ = 162.8, 162.3, 161.8, 161.3, 134.9, 129.5, 129.2, 129.1, 128.8, 128.6, 126.1, 123.5, 120.6, 117.6, 115.8, 69.2, 62.2, 19.1 ppm.

HRMS (ESI^+): *m/z* = 389.1802 $[M+Na]^+$ (calcd. 389.1782 for $C_{16}H_{30}O_9Na$). Note: Under ESI conditions K^+ is replaced with ubiquitous Na^+ (suggesting that the cage binds Na^+ preferably).

Synthesis of 3

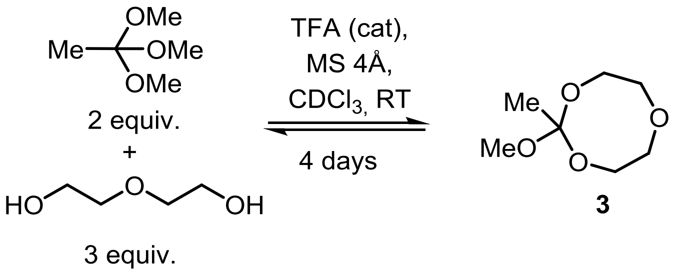

Diethylene glycol (DEG, 0.42 mmol, 39.9 µL) and 4 Å molecular sieves (500 mg) was added to 14 mL $CDCl_3$. After 24h, trimethyl orthoacetate (0.28 mmol, 36.0 µL) and 10 µL of a TFA stock solution (36.8 µL TFA in 1.96 mL $CDCl_3$) was added to this reaction mixture. The reaction progress was monitored regularly by ^{1}H NMR spectroscopy. After 4 days **3** was obtained as major product (see Supplementary Figure 21).

Synthesis of [Na$^+$⊂*o*-Me$_2$-1.1.1]BArF$^-$ via compound 3

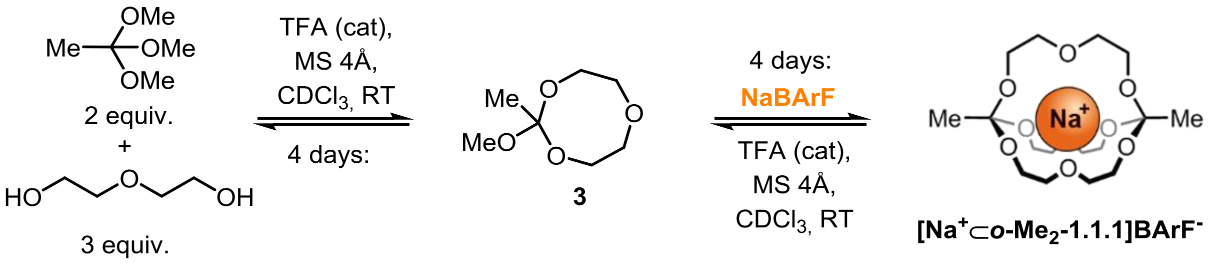

NaBArF (0.12 mmol) was dissolved in 2 mL of 1:1 mixture of acetonitrile and acetone and the mixture was dried for 3 days over 4 Å molecular sieves (ca. 200 mg). The solution was filtered and the solvent was removed under a stream of dry nitrogen to obtain anhydrous NaBArF. 6 mL of the reaction mixture containing **3** as major product (procedure see before) and 4 Å molecular sieves (ca. 500 mg) soaked with $CDCl_3$ was added to the dried NaBArF. The reaction progress was monitored regularly by ^{1}H NMR spectroscopy. After only 2 hours, **[Na$^+$⊂*o*-Me$_2$-1.1.1]BArF$^-$** was formed as predominant orthoester product and after 4 days, **[Na$^+$⊂*o*-Me$_2$-1.1.1]BArF$^-$** was obtained as exclusive orthoester product (see Supplementary Figure 22).

Sample preparation for Atom absorption and emission spectroscopy (AAS/AES)

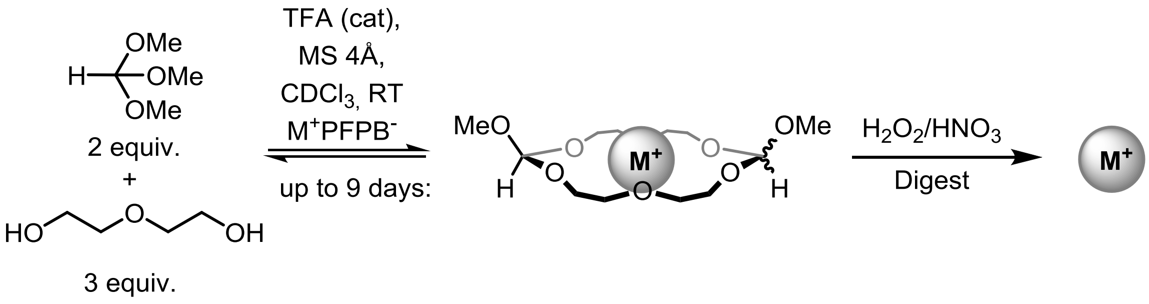

Lithium tetrakis(pentafluorophenyl)borate ethyl etherate (LiTPFPB, 0.06 mmol, 53.9 mg) or potassium tetrakis(pentafluorophenyl)borate (KTPFPB, 0.06 mmol, 44.4 mg) was added to a mixture of diethylene glycol (0.18 mmol, 17.1 µL) in $CDCl_3$ (6 mL). Molecular sieves (4 Å, ca. 1 g) were added and the reaction mixture was left to stand at room temperature. After two days 1 mol% TFA was added from a stock solution, the mixture was shaken and trimethyl orthoformate (0.12 mmol, 13.1 µL) was added. Aliquots (50 µL) for AAS/AES analysis were taken before addition of MS, after 2 days with MS, after 2 days of orthoester exchange and after nine days of orthoester exchange.

The aliquots were treated as follows: solvent was evaporated under a stream of nitrogen. 0.5 mL of a 4:1 mixture of concentrated HNO_3 and 30% H_2O_2 was added to the solid residue. The mixture was heated for 30 min at 100°C (digest of organic material). The samples were further diluted with water (p.a.) for the measurements.

Supplementary References

[1] Dr. J. M. Sanderson, www.dur.ac.uk/j.m.sanderson/science/downloads.html

[2] Cation exchange occurs with molecular sieves 4 Å, see: Breck, D. W., Eversole, W. G., Milton, R. M., Reed, T. B. & Thomas, T. L. Crystalline Zeolites. I. The Properties of a New Synthetic Zeolite, Type A. *J. Am. Chem. Soc.* **78**, 5963-5972 (1956).
